# Oxidative Additions of C−F Bonds to the Silanide Anion [Si(C_2_F_5_)_3_]^−^

**DOI:** 10.1002/anie.202116468

**Published:** 2022-02-28

**Authors:** Natalia Tiessen, Mira Keßler, Beate Neumann, Hans‐Georg Stammler, Berthold Hoge

**Affiliations:** ^1^ Universität Bielefeld Fakultät für Chemie Centrum für Molekulare Materialien Universitätsstraße 25 33615 Bielefeld Germany

**Keywords:** Fluorine, Oxidative Addition, Perfluorosilicates, Silanide Anion, Silicon

## Abstract

Compounds exhibiting main group elements in low oxidation states were found to mimic the reactivity of transition metal complexes. Like the latter, such main group species show a proclivity of changing their oxidation state as well as their coordination number by +2, therefore fulfilling the requirements for oxidative additions. Prominent examples of such main group compounds that undergo oxidative additions with organohalides R−X (R=alkyl, aryl, X=F, Cl, Br, I) are carbenes and their higher congeners. Aluminyl anions, which like carbenes and silylenes oxidatively add to strong σ‐bonds in R−X species, have been recently discovered. We present the first anion based upon a Group 14 element, namely the tris(pentafluoroethyl)silanide anion, [Si(C_2_F_5_)_3_]^−^, which is capable of oxidative additions towards C−F bonds. This enables the isolation of non‐chelated tetraorganofluorosilicate salts, which to the best of our knowledge had only been observed as reactive intermediates before.

## Graphical abstract

The tris(pentafluoroethyl)silanide anion, [Si(C_2_F_5_)_3_]^−^, digresses from the archetypical proclivity of nucleophilic substitutions, being capable of oxidative additions to aryl‐ and alkenylfluorides. Thus, tetraorganofluorosilicate salts devoid of chelating ligands, which had only been observed as reactive intermediates before, were isolated and fully characterized.

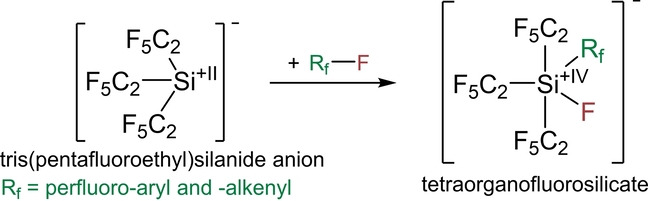

## Introduction

**Figure 1:**
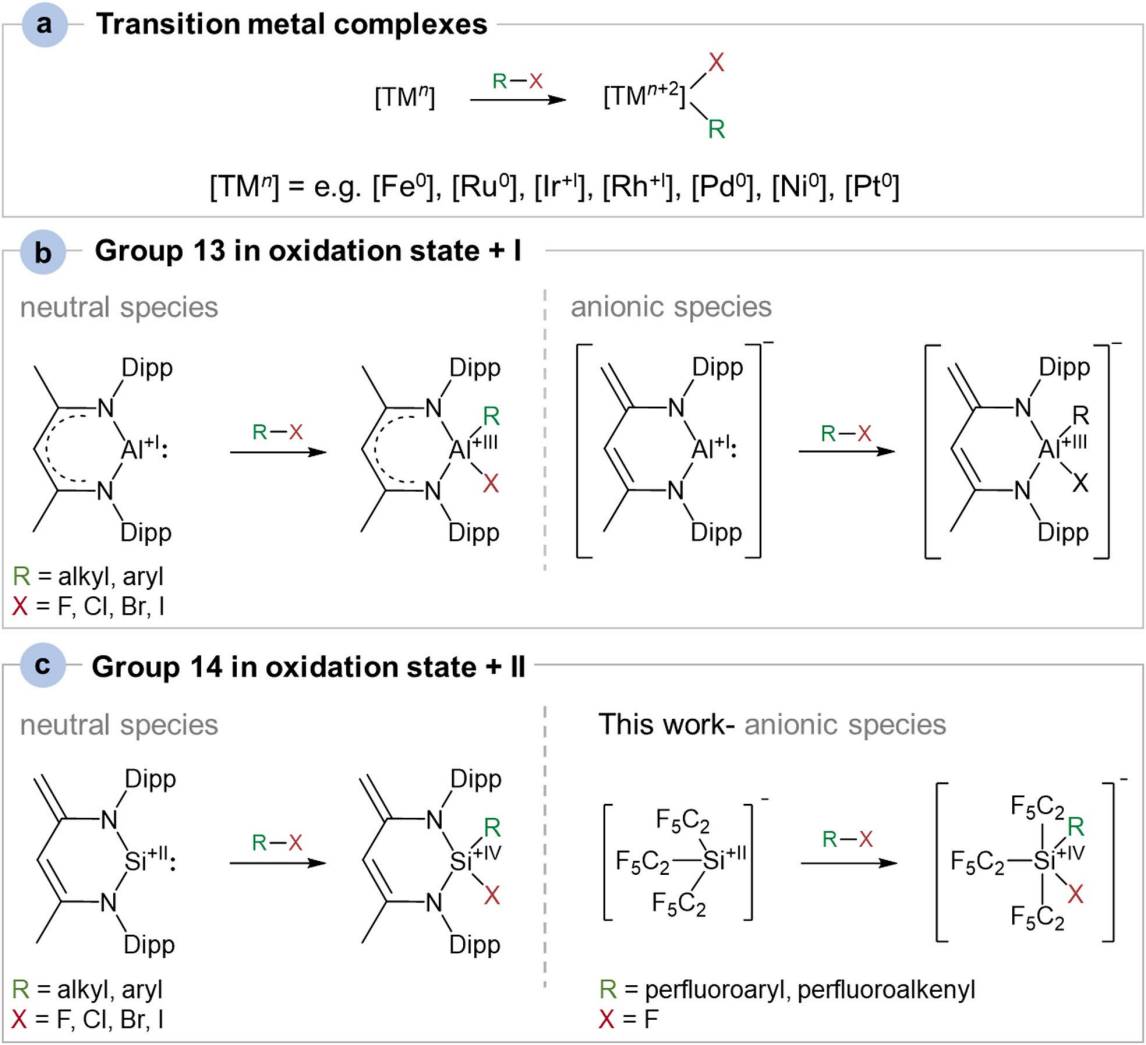
Examples for oxidative addition reactions of a) transition metal complexes.^[22]^ b) Group 13 in oxidation state +I [^1^, ^14^, ^16^] and c) Group 14 in oxidation state +II.[^1^, ^19^, ^23^]

Oxidative additions (OA) are key steps in many catalytic reactions including the activation of small molecules or C−C bond formations, and were traditionally considered as a domain of transition metal complex chemistry.[^1^, ^2^] Transition metals feature various stable oxidation states and are predestinated to expand the coordination number in their complexes which are indispensable prerequisites for OA reactions.^[3]^ In contrast, it takes great effort to lure main group elements out of their archetypical oxidation states. Over the past few decades tremendous progress has been made in the stabilization of compounds with low‐valent main group elements in low coordination such as B^+I^,^[4]^ Al^+I^,[^5^, ^6^, ^7^] Si^+II^,[^7^, ^8^] Ge^+II^,[^7^, ^9^] Si^0 [10]^ and Ge^0^.^[11]^ Such species are formal analogues of alkenes, alkynes, carbenes and carbenoids.^[12]^ Owing to their energetically high lying HOMOs (highest occupied molecular orbitals) and low lying, easily accessible LUMOs (lowest unoccupied molecular orbitals) these molecules are highly reactive and mimic the reactivity of transition metal systems in activating strong σ‐bonds and small molecules such as CO_2_, CO and H_2_.^[13]^ In addition to systems featuring multiple bonds with heavy main group elements, the heavy congeners of carbenes from Groups 14 (tetrelenes) and 13 (trielenes) have recently attracted great attention. Within Group 13 considerable progress has been made in isolating aluminum(+I) anions (“aluminyls”), which similar to their neutral Al^+I^ carbene congeners^[14]^ undergo OA reactions to a range of σ‐bonds including the C−F bond[^1^, ^15^, ^16^] as the strongest carbon–element σ‐bond (Figure 1). On the other hand, silylenes are probably the most prominent carbene homologues from Group 14 as they are versatile and valuable synthons in organosilicon chemistry, where they for example participate in a range of side‐on additions to multiple bonds present in alkenes, alkynes, ketones, imines, azides as well as heteroallenes X=C=Y (X, Y=O, S, NR with R=alkyl).[^5^, ^7^, ^17^] They are also proficient at adding oxidatively to strong σ‐bonds such as C−X bonds (X=I, Br, Cl, F) affording neutral tetra‐ and pentacoordinated silicon(+IV) compounds (Figure 1).[^1^, ^18^, ^19^] A zwitterionic silanide was stated to show Lewis amphoteric behavior and to support both oxidation states +II and +IV at the silicon center therefore exhibiting an unusual reactivity.^[20]^ We recently reported on the tris(pentafluoroethyl)silanide anion, [Si^+II^(C_2_F_5_)_3_]^−^, which due to its strong electron withdrawing C_2_F_5_ groups reacts not only as a typical Lewis base but also as a Lewis acid.^[21]^

This Lewis amphoteric character and the ability of pentafluoroethylated silanes to expand their coordination number makes the [Si(C_2_F_5_)_3_]^−^ anion a promising candidate for OA reactions. Thus, the [Si(C_2_F_5_)_3_]^−^ anion mirrors the reactivity of silylenes in adding side‐on to C=E (E=O, S) double bonds.^[21]^ On top of this, studies on the proficiency of the [Si(C_2_F_5_)_3_]^−^ anion to undergo OA reactions with C−X σ‐bonds are intriguing, as it is well known that anions predominantly serve as nucleophiles in substitution processes. To the best of our knowledge, to date there are no silanide anions involved in oxidative additions to C−X bonds. Even the so‐called silylenoids, which are analogues of carbenoids and often display the behavior of silylenes, react with organohalides R−X to produce substitution products.^[24]^

Consequently, we would like to add an essential chapter to our knowledge of fundamental silicon chemistry in presenting the tris(pentafluoroethyl)silanide anion, [Si(C_2_F_5_)_3_]^−^, which represents a potent candidate for oxidative additions to aryl‐ and alkenylfluorides.

## Results and Discussion

**Scheme 1:**
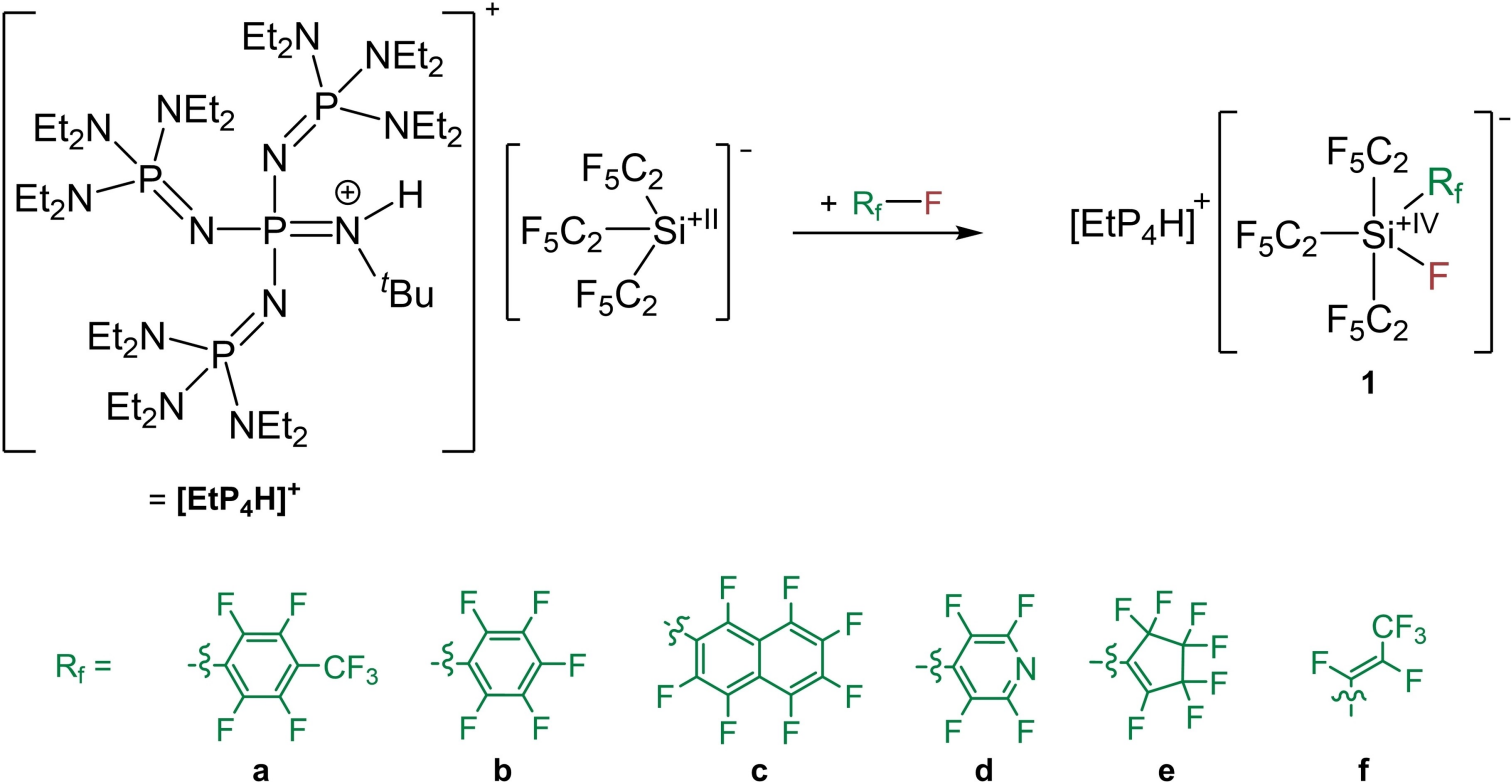
Oxidative addition of [EtP_4_H][Si(C_2_F_5_)_3_] to perfluoro‐aryls and ‐olefins.

Stable phosphazenium salts of the tris(pentafluoroethyl)silanide anion, [Si(C_2_F_3_)_3_]^−^, are easily accessible by deprotonation of the corresponding tris(pentafluoroethyl)silane, Si(C_2_F_5_)_3_H, and serve as ideal precursors for our purpose.^[21]^ Thus we investigated the reaction of [EtP_4_H][Si(C_2_F_5_)_3_] ([EtP_4_H]^+^=[{(Et_2_N)_3_P=N}_3_P=N(H)^*t*^Bu]^+^) with perfluorinated pyridine (**d**‐F), toluene (**a**‐F), benzene (**b**‐F), naphthalene (**c**‐F), cyclopentene (**e**‐F) and propene (**f**‐F), which indeed afforded the respective addition products. The reactions, when performed in diethyl ether at room temperature, smoothly lead to fluorosilicate salts [EtP_4_H]**1a**–**f** in quantitative yields (Scheme 1). Notably, the tris(pentafluoroethyl)silanide anion reacts immediately with perfluorinated compounds R_f_−F (R_f_=**a**, **c**–**f**), while the reaction with hexafluorobenzene (R_f_=**b**) needs several days to reach completion.

**Figure 2:**
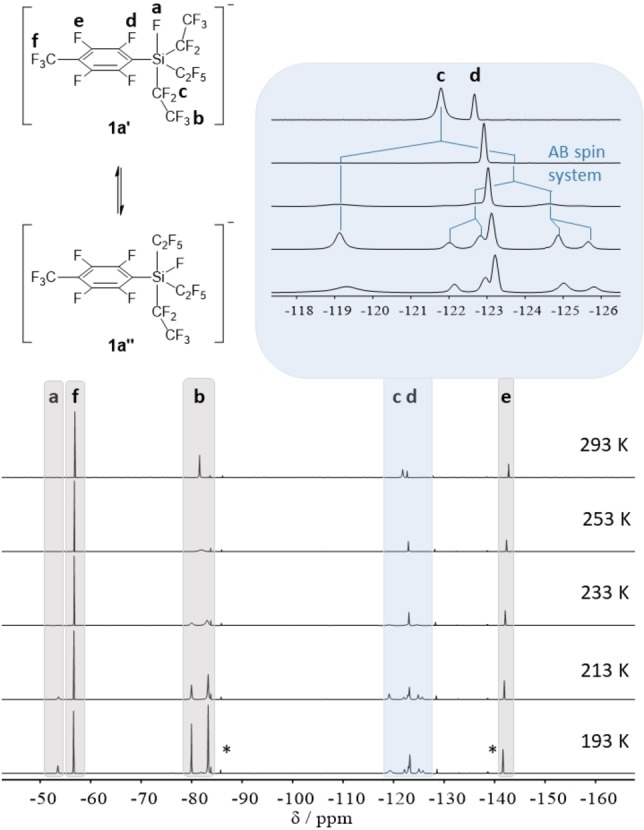
Temperature dependence of the ^19^F NMR spectrum of **1 a** (solvent: CD_2_Cl_2_, −50 ppm to −160 ppm). Two possible isomers **1 a′** and **1 a′′** are depicted. The changes within the spectra are completely reversible upon heating or cooling. *Signals for HC_2_F_5_.

All perfluoroorganofluorosilicate salts [EtP_4_H]**1a**–**f** were characterized by multinuclear NMR spectroscopy. Relevant ^29^Si and ^19^F NMR data are compiled in Table 1. The obtained ^29^Si NMR chemical shifts of the products are in agreement with pentacoordinated silicates which typically resonate from −95 to −150 ppm.^[25]^ In line with the so‐called Berry pseudorotation^[26]^ ^19^F NMR spectra of **1 a**–**f** generally show one set of broadened resonances for the C_2_F_5_ groups pointing to a rapid exchange of the axial and equatorial ligands in solution, which renders the C_2_F_5_ groups equal on the NMR time scale. The ^19^F NMR spectra of [EtP_4_H][Si(C_2_F_5_)_3_(C_7_F_7_)F] (**1 a**) were recorded at different temperatures (Figure 2).

**Table 1:** NMR spectroscopic data of [EtP_4_H]**1 a**–**f**.

	δ(CF_2_C**F**_3_)/ [ppm]	δ(C**F**_2_CF_3_)/ [ppm]	δ(Si)/ [ppm]	δ(Si**F**)/ [ppm]	^1^ *J*_Si,F_/ [Hz]
**1 a**	−81.6	−121.8	−104.8	−53.5^[a]^	296
**1 b**	−81.4	−121.9	−104.6	–	294
**1 c**	−81.4	−121.7	−104.4	–	295
**1 d**	−81.7	−121.7	−105.0	–	294
**1 e**	−81.5	−121.6	−105.6	−64.9	203
**1 f**	−82.0	−123.1	−107.3	–	309

[a] observed at 193 K.

While only one signal for the CF_3_ units of the C_2_F_5_ groups in **1 a** is detected at 293 K, this signal broadens at 253 K and splits into two initially broad, then sharp singlets at lower temperatures with a 1 : 2 integral ratio. Accordingly, the CF_2_ resonance also splits into a singlet and an AB spin system with the same integral ratio of 1 : 2. The AB spin system results from the diastereotopic fluorine atoms of two equatorial or axial CF_2_ units for example like in the isomers **1 a′** or **1 a′′**. The ^2^ *J*_F,F_ coupling constant is 385 Hz. The fluorine atom directly attached to the silicon center cannot be detected at room temperature but emerges at lower temperatures at −53.5 ppm as a broad signal. To further elucidate the mechanism of the Berry pseudorotation, quantum mechanical calculations were performed on the B3LYP/6‐31+G(3d,p) level of theory.^[27]^ To reduce computational cost, the C_2_F_5_ groups were substituted by the electronically similar CF_3_ groups. Geometry optimization of structures with the substituent **a** in axial position inevitably yielded structures with **a** in equatorial position. Two optimized structures with no significant difference in energy, corresponding to **1 a′** and **1 a′′**_,_ were found. Thus, it is conceivable, that mainly the fluorine atom and the C_2_F_5_ substituents exchange by means of Berry pseudorotation. Depending on the nature of the substituents R_f_ and the activation barrier of the Berry pseudorotation all effects are more or less pronounced in the NMR spectra. Thus, for example the signals of derivative **1 f** are less broadened and the fluorine atom attached to the silicon center is detectable even at room temperature.

The negative ESI mass spectra of the silicate salts [EtP_4_H]**1 a**–**f** reveal the respective molecular ion peaks and elemental analyses confirm the purity of all salts. All silicates [EtP_4_H]**1 a**–**f** are extremely moisture sensitive releasing HC_2_F_5_. The salts decompose slowly when stored under nitrogen for several days at room temperature.

**Figure 3:**
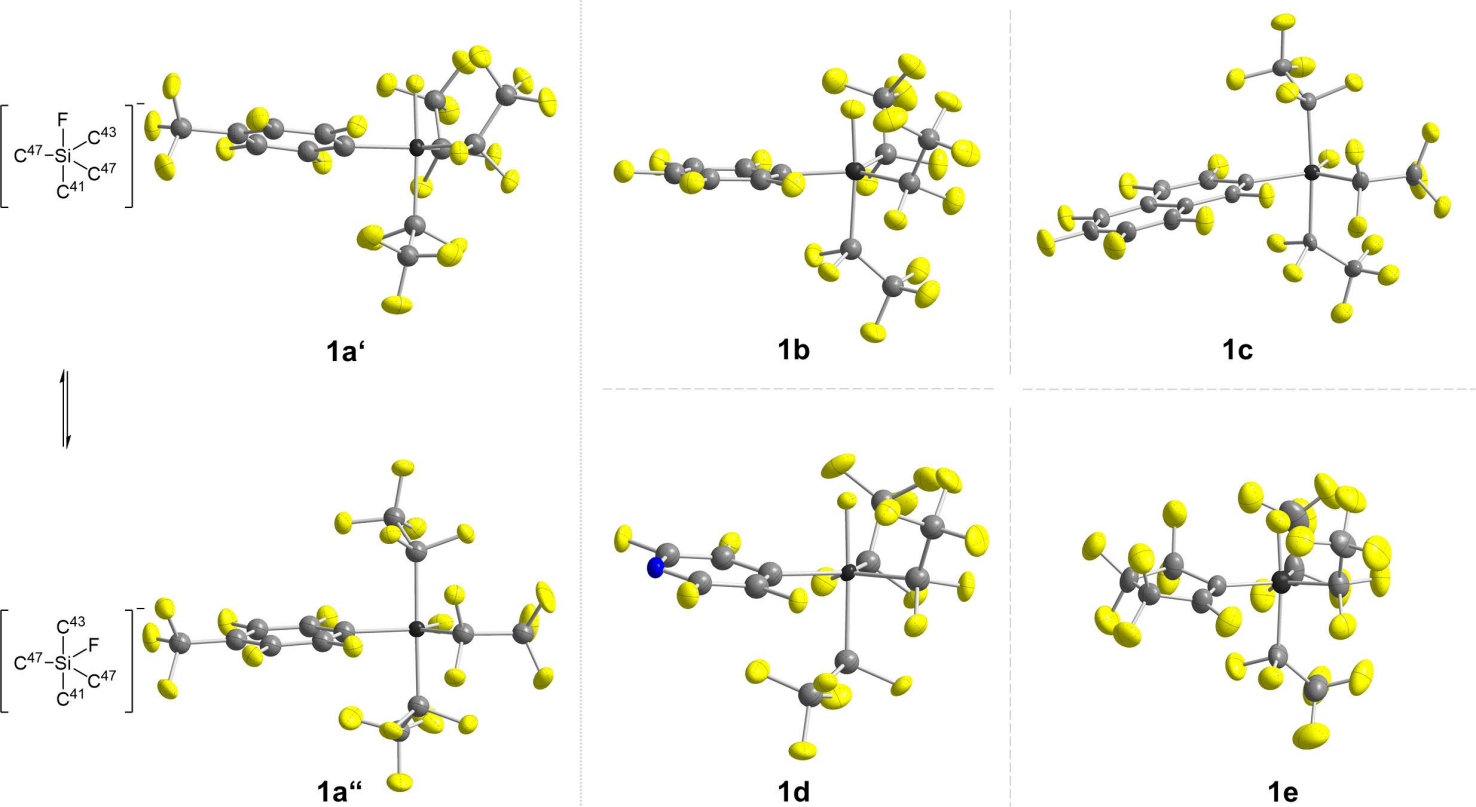
Molecular structures of the fluorosilicates [EtP_4_H]**1 a**–**e**.^[31]^ Thermal ellipsoids are represented with a probability of 50 %. The cation, minor occupied disordered atoms and the second ion have been omitted for clarity. Both isomers **1 a′** and **1 a′′** of **1 a** are depicted. Selected bond lengths and angles are compiled in Table 2.

Single crystals suitable for X‐ray diffraction analyses were obtained of all compounds, except for [EtP_4_H]**1 f**. [EtP_4_H]**1 a** crystallizes in the triclinic space group *P* $\bar{1}$ with 4 molecules per unit cell (Figure 3). The asymmetric unit contains the two different conformational isomers **1 a′** and **1 a′′**. Both isomers display the geometry of slightly distorted trigonal bipyramids (τ_5_=0.77 for **1 a′** and τ_5_=0.87 for **1 a′′**). According to the VSEPR model^[28]^ **1 a′** shows the expected geometry with the fluorine atom at the axial position. However, in **1 a′′** the fluorine atom is found in equatorial and two C_2_F_5_ groups in axial position. As expected, all axial bonds are elongated when compared to the equatorial bonds. While the axial Si−F bond in **1 a′** amounts to 167.0(1) pm, the equatorial Si−F bond length in **1 a′′** is 160.6(1) pm. The same effect is observed for the axial and equatorial Si−C‐bond lengths (205.0(2) pm vs. 197.4(2) and 197.9(2) pm in **1 a′** and 203.1(2) pm and 203.3(2) pm vs. 199.6(2) pm in **1 a′′**). All other silicate salts [EtP_4_H]**1 b**–**e** either crystallize similar to **1 a′** with one C_2_F_5_ group and the fluorine atom in axial position (**1 b**, **1 d** and **1 e**) or similar to **1 a′′** with two C_2_F_5_ groups in axial position (**1 c**). Consistent with the quantum chemical calculations, the perfluoroaryl substituents always occupy equatorial positions. Selected bond lengths and angles as well as calculated τ_5_ values are summarized in Table 2.

**Table 2:** Selected bond lengths [ppm] and angles [°] of the molecular structures of **1 a′**, **1 a′′**, **1 b**. **1 c**, **1 d** and **1 e**.

	Space group	Axial bond lengths			Axial angle	Equatorial bond lengths				τ value
		Si−F	Si−C41	Si−C43	CSiC/FSiC	Si−F	Si−C43	Si−C45	Si−C47	
**1 a′**	*P* $\bar{1}$	167.0(1)	205.0(2)	–	176.1(1)	–	197.9(2)	197.3(2)	193.4(2)	0.77
**1 a′′**	*P* $\bar{1}$	–	203.1(2)	203.3(2)	176.3(1)	160.6(1)	–	199.6(2)	193.0(2)	0.87
**1 b**	*P*2_1_2_1_2_1_	166.3(3)	206.5(4)	–	173.4(2)	–	199.1(5)	196.3(5)	193.7(4)	0.76
**1 c**	*P* $\bar{1}$	–	204.2(3)	201.5(2)	172.2(1)	160.8(1)	–	199.1(2)	193.0(2)	0.80
**1 d**	*P* $\bar{1}$	166.5(1)	205.6(1)	–	177.2(1)	–	198.0(2)	197.1(2)	193.2(1)	0.87
**1 e**	*P*2_1_	167.5(4)	204.1(7)	–	174.6(3)	–	197.4(7)	197.3(7)	190.7(5)	0.85

The fluorosilicate salts synthesized herein represent the first examples of isolated and fully characterized tetraorganofluorosilicate salts without the support of chelating ligands. In contrast to pentaorganosilicates, which have been recently studied in detail,^[26b]^ tetraorganofluorosilicates in most cases have merely been observed as reactive intermediates.[^26b^, ^29^] As briefly indicated, the only other isolated and structurally characterized tetraorganofluorosilicate salts contain chelating ligands.^[30]^

## Computational Studies

To support our experimental findings, some computational studies were performed (proton affinities: B3LYP/6‐311+G(3d,2p) reaction pathways: B3LYP/6‐31+G(3d,p); for further information see Supporting Information.^[27]^ As we described in a previous article, perfluoroalkyl‐substituted silanides exhibit energetically lower‐lying frontier orbital in comparison to their non‐fluorinated derivatives. This results in a lower Lewis basicity and a higher Lewis acidity of perfluoroalkyl‐substituted silanides.^[21]^ In order to further support these findings, we additionally calculated the proton affinities (PAs) of [Si(CF_3_)_3_]^−^, [Si(C_2_F_5_)_3_]^−^, and [Si(CH_3_)_3_]^−^, [Si(C_2_H_5_)_3_]^−^. The introduction of fluorine decreases the proton affinity (PA) as well as the Lewis basicity of the silanides [Si(CF_3_)_3_]^−^ (PA=322.6 kcal mol^−1^) and [Si(C_2_F_5_)_3_]^−^ (PA=320.1 kcal mol^−1^) compared to their non‐fluorinated analogues [Si(CH_3_)_3_]^−^ (PA=385.5 kcal mol^−1^) and [Si(C_2_H_5_)_3_]^−^ (PA=382.2 kcal mol^−1^).

Again, for further calculations the C_2_F_5_ groups were substituted by CF_3_ groups to reduce computational cost. Table 3 shows the calculated kinetic and thermodynamic parameters for the addition of [Si(CF_3_)_3_]^−^ to R_f_−F. The overall energy release for the reactions is virtually identical for R_f_=**a**–**e**. In the case of R_f_=**f** it is slightly lower.

**Table 3:** Change in energy (Δ*E*), enthalpy (Δ*H*) and Gibbs free energy (Δ*G*) in kcal mol^−1^ by the addition of [Si(CF_3_)_3_]^−^ to R_f_−F.

	Transition state^[a]^			Product		
R_f_−F	Δ*E*	Δ*H*	Δ*G*	Δ*E*	Δ*H*	Δ*G*
**a**‐F	10.2	10.6	22.4	−63.8	−63.6	−50.2
**b**‐F	15.6	16.0	27.9	−63.6	−63.4	−50.5
**c**‐F				−62.4	−62.2	−49.3
**d**‐F				−63.4	−63.2	−50.4
**e**‐F				−64.7	−64.5	−51.4
**f**‐F				−55.8	−55.5	−42.9

[a] Exemplarily calculated for **a**‐F and **b**‐F.

**Figure 4:**
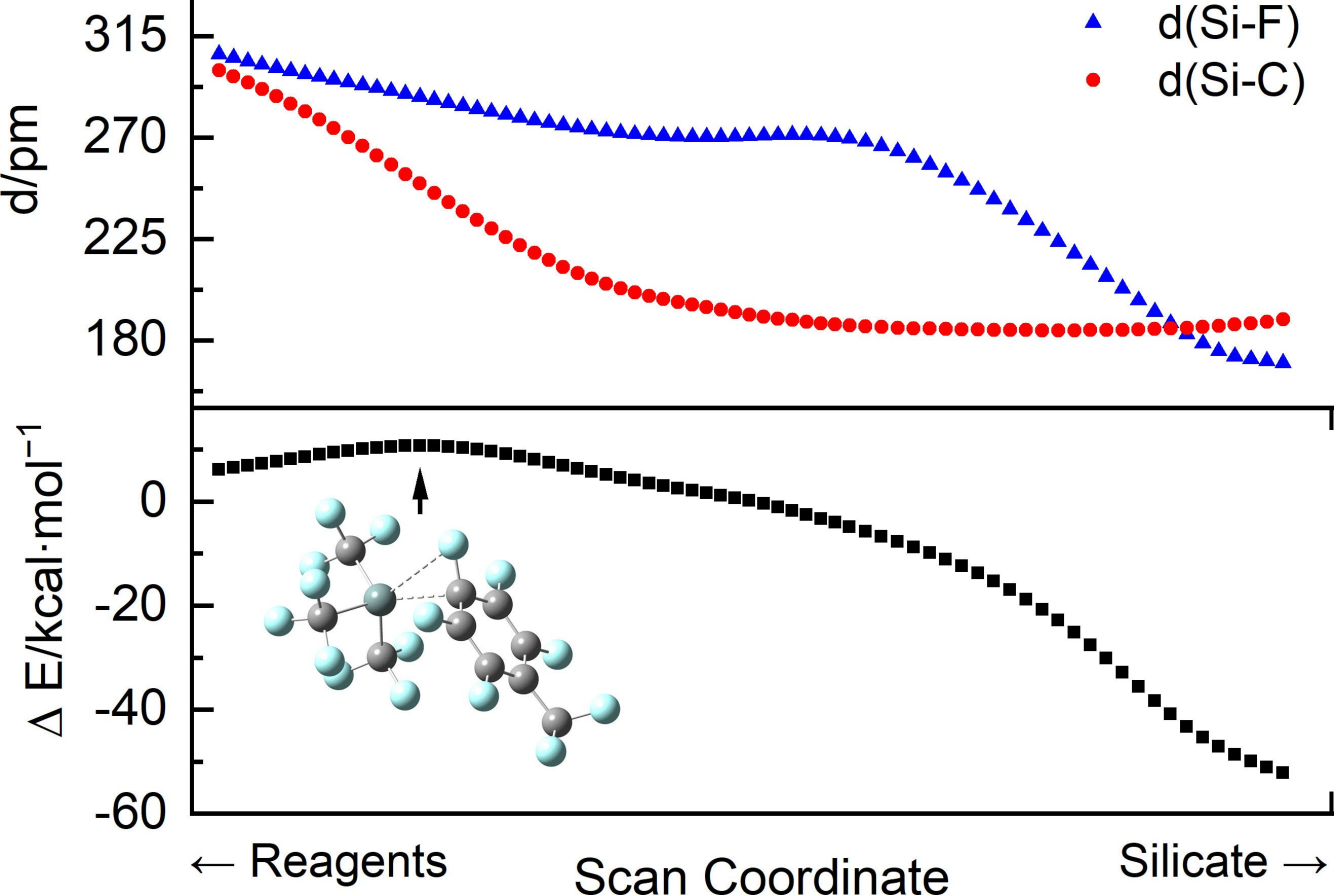
Si−F and Si−C_Ar_ distance as well as the change in electronic energy relative to the reagents along the reaction path of the oxidative addition of **a**‐F to [Si(CF_3_)_3_]^−^ obtained by an IRC scan. The transition state is shown and marked with an arrow.

**Figure 5:**
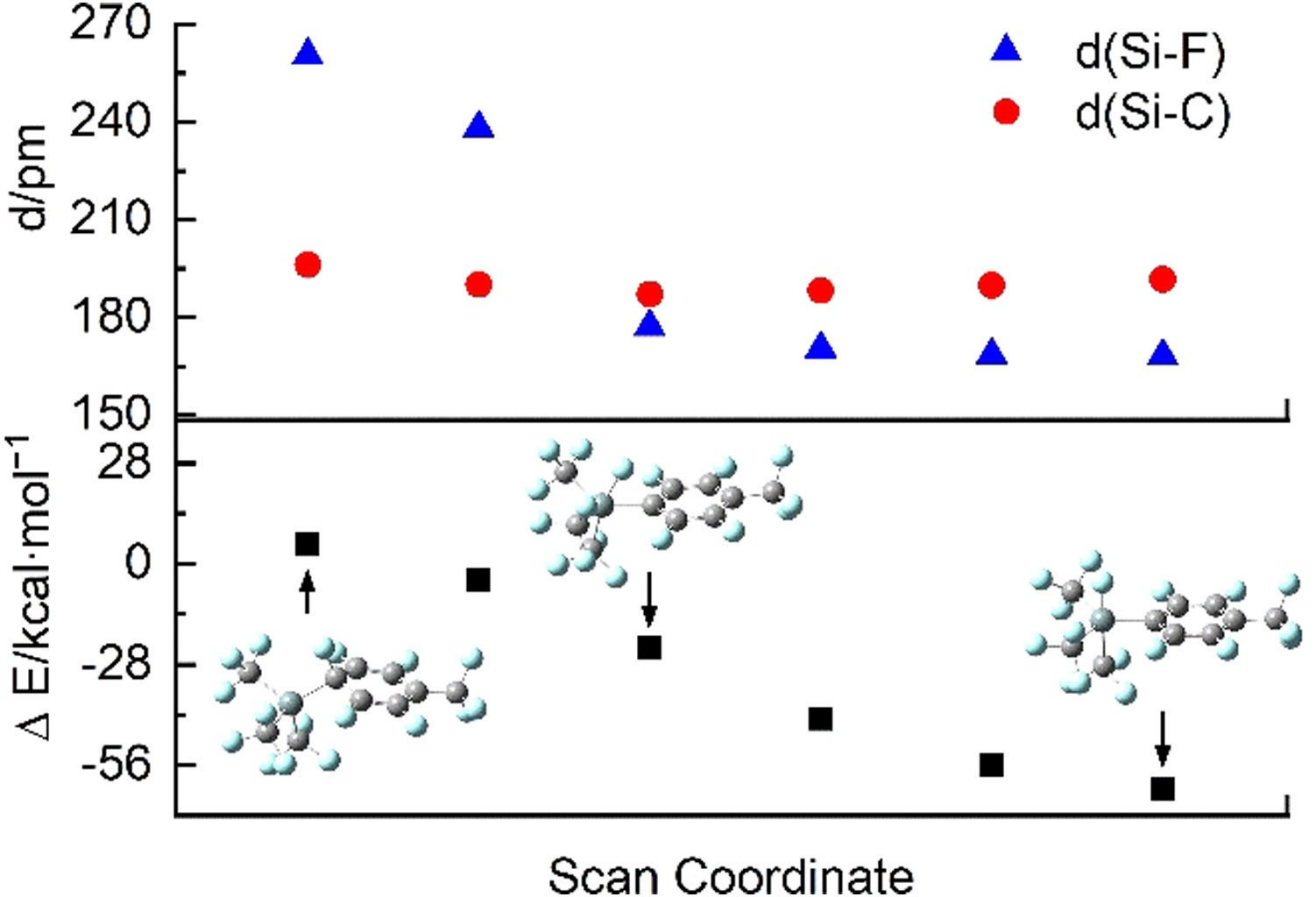
Si−F and Si−C_Ar_ distance as well as the change in electronic energy relative to the reagents of the oxidative addition of **a**‐F to [Si(CF_3_)_3_]^−^ obtained by a relaxed potential energy scan along the C−F bond that is cleaved upon reaction.

**Figure 6:**
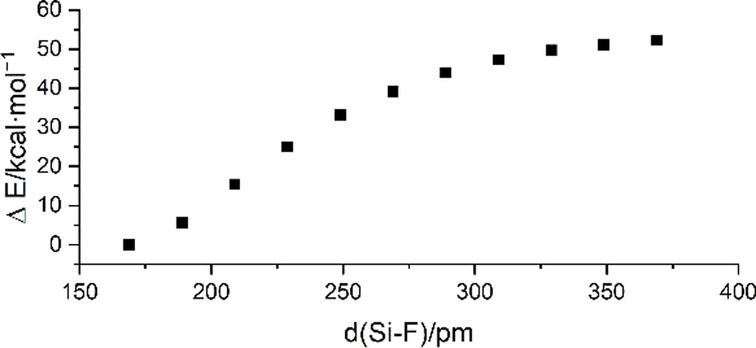
Change in electronic energy relative to the electronic energy of the silicate [Si(CF_3_)_3_{C_6_F_4_‐4‐(CF_3_)}F]^−^ obtained by a relaxed potential energy scan along the Si−F bond.

With regard to the reaction pathway, the question arises if the oxidative addition proceeds in a concerted way or if it is a stepwise process involving the initial substitution of a fluoride ion by the silanide. Exemplarily, the reaction of [Si(CF_3_)_3_]^−^ with **a**‐F was examined in greater detail. Only one transition state and no further intermediates were found along the pathway, indicating that the oxidative addition is a concerted process leading to the product [Si(CF_3_)_3_{C_6_F_4_(CF_3_)}F]^−^ (**1 a**). Figure 4 shows the results of an intrinsic reaction coordinate (IRC) scan around the transition state. Although the respective Si−C distance decreases more rapidly than the Si−F distance, the fluorine atom is not dissociated along the process for the following reasons: Upon subsequent elongation of the respective C−F bond the energy decreases and at a certain point the Si−F distance comes below the Si−C distance (Figure 4 and 5). Furthermore, the energy rapidly and smoothly decreases by subsequent nearing of a fluoride ion on the hypothetically by fluoride abstraction produced silane Si(CF_3_)_3_[C_6_F_4_‐4‐(CF_3_)] (Figure 6).

The activation barrier of the addition of [Si(CF_3_)_3_]^−^ to hexafluorobenzene (**b**‐F) is higher than of the addition to (trifluoromethyl)pentafluorobenzene (**a**‐F). Since the reaction of [Si(C_2_F_5_)_3_]^−^ with **b**‐F proceeded slower than the reaction with **a**‐F, this is consistent with the experimental results. The different reactivity might be rationalized by the different electron donating and withdrawing abilities of a fluorine and a trifluoromethyl substituent in an aromatic system. As illustrated by the Hammett parameters, the π donation of a fluorine atom has a large influence in *para* position (σ_*m*_=0.34, σ_p_=0.06) whereas the trifluormethyl group displays π acidity (σ_*m*_=0.43, σ_p_=0.54).^[32]^ Thus, the carbon atom attacked by the nucleophilic part of the silanide anion is significantly more electron rich in **b**‐F than in **a**‐F.

## Conclusion

We highlighted the potential of the tris(pentafluoroethyl)silanide anion to easily cleave C−F bonds in aryl‐ and alkenylfluorides by oxidative addition. Thus, it digresses from the archetypical proclivity of anions to undergo nucleophilic substitutions. Anions from Group 14 elements, which are capable of OA reactions, open the door to the syntheses of unusual, novel compounds. Hence, tetraorganofluorosilicate salts [EtP_4_H]**1 a**–**f** devoid of chelating ligands, which had only been observed as reactive intermediates before, were now isolated and fully characterized. NMR spectroscopy of fluorosilicate [EtP_4_H]**1 a** at various temperatures shows a rapid exchange of the C_2_F_5_ groups and the fluorine atom in solution. Different isomers with either two C_2_F_5_ groups or one C_2_F_5_ group and the fluorine atom in axial position are present in the solid state. Investigations concerning the ability of the [Si(C_2_F_5_)_3_]^−^ anion to oxidatively add to other σ‐bonds are in progress.

## Conflict of interest

The authors declare no conflict of interest.

1

## Supporting information

As a service to our authors and readers, this journal provides supporting information supplied by the authors. Such materials are peer reviewed and may be re‐organized for online delivery, but are not copy‐edited or typeset. Technical support issues arising from supporting information (other than missing files) should be addressed to the authors.

Supporting Information

Supporting Information

Supporting Information

Supporting Information

Supporting Information

Supporting Information

Supporting Information

Supporting Information

Supporting Information

Supporting Information

Supporting Information

## Data Availability

The data that support the findings of this study are available in the Supporting Information of this article.
