# Supplementary material for: Oxidative Additions of C−F Bonds to the Silanide Anion [Si(C2F5)3]−
Source: Angew Chem Int Ed Engl. 2022 Feb 28;61(17):e202116468. doi: 10.1002/anie.202116468 (PMC9310575; doi:10.1002/anie.202116468)

checkCIF/PLATON report

You have not supplied any structure factors. As a result the full set of tests cannot be run.

THIS REPORT IS FOR GUIDANCE ONLY. IF USED AS PART OF A REVIEW PROCEDURE FOR PUBLICATION, IT SHOULD NOT REPLACE THE EXPERTISE OF AN EXPERIENCED CRYSTALLOGRAPHIC REFEREE.

No syntax errors found. CIF dictionary Interpreting this report

Datablock: compound1a

Bond precision: C-C = 0.0022 A Wavelength=1.54184

Cell:	a=13.5000(2)	b=22.1410(3)	c=23.8194(4)
	alpha=89.9961(12)	beta=87.2836(12)	gamma=89.4024(11)
Temperature:	100 K		

	Calculated	Reported
Volume	7111.32(19)	7111.34(18)
Space group	P -1	P -1
Hall group	-P 1	-P 1
Moiety formula	2(C40 H100 N13 P4), C13 F23 Si, C13 F22.87 Si, 0.132(F)	C40 H100 N13 P4, C13 F23 Si
Sum formula	C106 H200 F46 N26 P8 Si2	C53 H100 F23 N13 P4 Si
Mr	3016.86	1508.42
Dx,g cm-3	1.409	1.409
Z	2	4
Mu (mm-1)	2.082	2.082
F000	3160.0	3160.0
F000'	3177.37	
h,k,lmax	16,27,29	16,27,29
Nref	28986	28494
Tmin,Tmax	0.678,0.747	0.905,1.000
Tmin'	0.615	

Correction method= # Reported T Limits: Tmin=0.905 Tmax=1.000
AbsCorr = MULTI-SCAN

Data completeness= 0.983 Theta(max)= 74.238

R(reflections)= 0.0332(26204) wR2(reflections)= 0.0892(28494)

S = 1.013 Npar= 1817

The following ALERTS were generated. Each ALERT has the format
test-name_ALERT_alert-type_alert-level.
Click on the hyperlinks for more details of the test.

Alert level C

```
PLAT230_ALERT_2_C Hirshfeld Test Diff for    Si2      --C94      .        5.9 s.u.
PLAT230_ALERT_2_C Hirshfeld Test Diff for    Si2      --C96      .        6.0 s.u.
PLAT230_ALERT_2_C Hirshfeld Test Diff for    Si1      --C45      .        5.9 s.u.
PLAT230_ALERT_2_C Hirshfeld Test Diff for    Si1      --C47      .        5.1 s.u.
PLAT420_ALERT_2_C D-H Bond Without Acceptor  N1       --H1       .     Please Check
PLAT420_ALERT_2_C D-H Bond Without Acceptor  N14      --H14      .     Please Check
PLAT790_ALERT_4_C Centre of Gravity not Within Unit Cell: Resd.  #          1 Note
              C40 H100 N13 P4
```

Alert level G

```
PLAT003_ALERT_2_G Number of Uiso or Uij Restrained non-H Atoms ...         16 Report
PLAT042_ALERT_1_G Calc. and Reported Moiety Formula Strings Differ     Please Check
PLAT045_ALERT_1_G Calculated and Reported Z Differ by a Factor ...       0.50 Check
PLAT178_ALERT_4_G The CIF-Embedded .res File Contains SIMU Records          2 Report
PLAT230_ALERT_2_G Hirshfeld Test Diff for    C98      --C99A     .        8.0 s.u.
PLAT230_ALERT_2_G Hirshfeld Test Diff for    C50      --C53A     .        7.3 s.u.
PLAT242_ALERT_2_G Low    'MainMol' Ueq as Compared to Neighbors of        C95 Check
PLAT242_ALERT_2_G Low    'MainMol' Ueq as Compared to Neighbors of        C97 Check
PLAT242_ALERT_2_G Low    'MainMol' Ueq as Compared to Neighbors of       C106 Check
PLAT242_ALERT_2_G Low    'MainMol' Ueq as Compared to Neighbors of        C42 Check
PLAT242_ALERT_2_G Low    'MainMol' Ueq as Compared to Neighbors of        C46 Check
PLAT302_ALERT_4_G Anion/Solvent/Minor-Residue Disorder (Resd  3  )        11% Note
PLAT302_ALERT_4_G Anion/Solvent/Minor-Residue Disorder (Resd  4  )        10% Note
PLAT302_ALERT_4_G Anion/Solvent/Minor-Residue Disorder (Resd  5  )       100% Note
PLAT304_ALERT_4_G Non-Integer Number of Atoms in ..... (Resd  4  )      36.87 Check
PLAT304_ALERT_4_G Non-Integer Number of Atoms in ..... (Resd  5  )       0.13 Check
PLAT432_ALERT_2_G Short Inter X...Y Contact  F23B     ..C50              2.40 Ang.
                                                      x,y,z  =      1_555 Check
PLAT432_ALERT_2_G Short Inter X...Y Contact  F23B     ..C51              2.67 Ang.
                                                      x,y,z  =      1_555 Check
PLAT434_ALERT_2_G Short Inter HL..HL Contact F19      ..F23B             2.24 Ang.
                                                      x,y,z  =      1_555 Check
PLAT790_ALERT_4_G Centre of Gravity not Within Unit Cell: Resd.  #          4 Note
              C13 F22.87 Si
PLAT790_ALERT_4_G Centre of Gravity not Within Unit Cell: Resd.  #          5 Note
              F
PLAT860_ALERT_3_G Number of Least-Squares Restraints ............         150 Note
PLAT941_ALERT_3_G Average HKL Measurement Multiplicity ..........         4.7 Low
```

0 **ALERT level A** = Most likely a serious problem - resolve or explain
0 **ALERT level B** = A potentially serious problem, consider carefully
7 **ALERT level C** = Check. Ensure it is not caused by an omission or oversight
23 **ALERT level G** = General information/check it is not something unexpected

2 ALERT type 1 CIF construction/syntax error, inconsistent or missing data

```
 17 ALERT type 2 Indicator that the structure model may be wrong or deficient
  2 ALERT type 3 Indicator that the structure quality may be low
  9 ALERT type 4 Improvement, methodology, query or suggestion
  0 ALERT type 5 Informative message, check
```

Validation response form

Please find below a validation response form (VRF) that can be filled in and pasted into your CIF.

```
# start Validation Reply Form
_vrf_PLAT230_compound1a
;
PROBLEM: Hirshfeld Test Diff for    Si2      --C94      .        5.9 s.u.
RESPONSE: ...
;
_vrf_PLAT420_compound1a
;
PROBLEM: D-H Bond Without Acceptor  N1       --H1       .     Please Check
RESPONSE: ...
;
_vrf_PLAT790_compound1a
;
PROBLEM: Centre of Gravity not Within Unit Cell: Resd.  #          1 Note
RESPONSE: ...
;
# end Validation Reply Form
```

It is advisable to attempt to resolve as many as possible of the alerts in all categories. Often the minor alerts point to easily fixed oversights, errors and omissions in your CIF or refinement strategy, so attention to these fine details can be worthwhile. In order to resolve some of the more serious problems it may be necessary to carry out additional measurements or structure refinements. However, the purpose of your study may justify the reported deviations and the more serious of these should normally be commented upon in the discussion or experimental section of a paper or in the "special_details" fields of the CIF. checkCIF was carefully designed to identify outliers and unusual parameters, but every test has its limitations and alerts that are not important in a particular case may appear. Conversely, the absence of alerts does not guarantee there are no aspects of the results needing attention. It is up to the individual to critically assess their own results and, if necessary, seek expert advice.

Publication of your CIF in IUCr journals

A basic structural check has been run on your CIF. These basic checks will be run on all CIFs submitted for publication in IUCr journals (*Acta Crystallographica*, *Journal of Applied Crystallography*, *Journal of Synchrotron Radiation*); however, if you intend to submit to *Acta Crystallographica Section C* or *E* or *IUCrData*, you should make sure that full publication checks are run on the final version of your CIF prior to submission.

Publication of your CIF in other journals

Please refer to the *Notes for Authors* of the relevant journal for any special instructions relating to CIF submission.

PLATON version of 13/07/2021; check.def file version of 13/07/2021

Datablock compound1a - ellipsoid plot

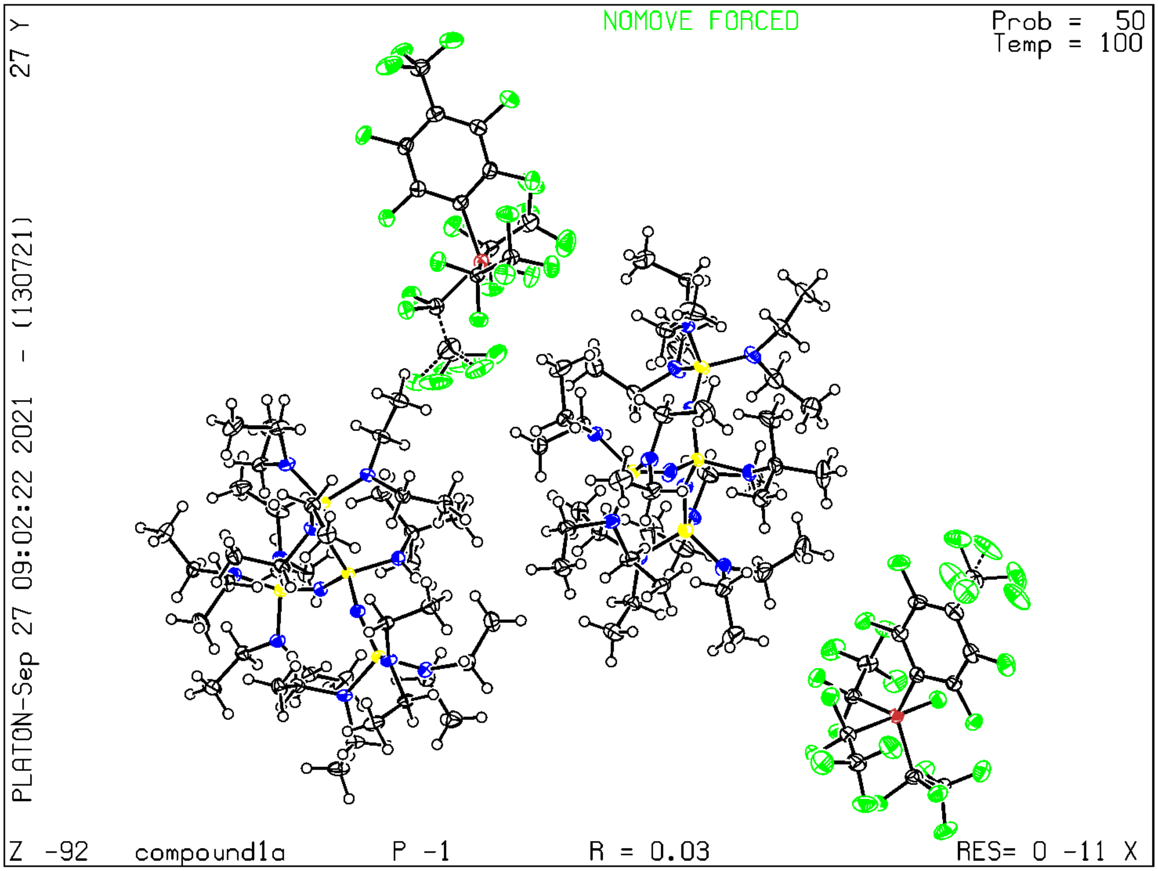

Supplement: Supplementary file 1 — Supporting Information [file ANIE-61-0-s003.pdf]
