# Supplementary material for: Oxidative Additions of C−F Bonds to the Silanide Anion [Si(C2F5)3]−
Source: Angew Chem Int Ed Engl. 2022 Feb 28;61(17):e202116468. doi: 10.1002/anie.202116468 (PMC9310575; doi:10.1002/anie.202116468)

checkCIF/PLATON report

You have not supplied any structure factors. As a result the full set of tests cannot be run.

THIS REPORT IS FOR GUIDANCE ONLY. IF USED AS PART OF A REVIEW PROCEDURE FOR PUBLICATION, IT SHOULD NOT REPLACE THE EXPERTISE OF AN EXPERIENCED CRYSTALLOGRAPHIC REFEREE.

No syntax errors found. CIF dictionary Interpreting this report

Datablock: compound1b

```
Bond precision:   C-C = 0.0067 A          Wavelength=1.54184

Cell:              a=11.8798(2)      b=16.3834(3)      c=35.4911(7)
                   alpha=90          beta=90           gamma=90
Temperature:       100 K

                  Calculated                       Reported
Volume            6907.7(2)                        6907.7(2)
Space group       P 21 21 21                       P 21 21 21
Hall group        P 2ac 2ab                        P 2ac 2ab
Moiety formula    C40 H100 N13 P4, C12 F21 Si      C40 H100 N13 P4, C12 F21 Si
Sum formula       C52 H100 F21 N13 P4 Si           C52 H100 F21 N13 P4 Si
Mr                1458.42                          1458.41
Dx,g cm-3         1.402                            1.402
Z                 4                                4
Mu (mm-1)         2.081                            2.081
F000              3064.0                           3064.0
F000'             3080.73
h,k,lmax          14,20,44                         14,20,43
Nref              14442[ 7945]                     14038
Tmin,Tmax         0.781,0.855                      0.977,1.000
Tmin'             0.718

Correction method= # Reported T Limits: Tmin=0.977 Tmax=1.000
AbsCorr = MULTI-SCAN

Data completeness= 1.77/0.97        Theta(max)= 76.058

R(reflections)= 0.0428( 12094)                 wR2(reflections)=
                                               0.0993( 14038)
S = 1.021                   Npar= 875
```

```
The following ALERTS were generated. Each ALERT has the format
       test-name_ALERT_alert-type_alert-level.
Click on the hyperlinks for more details of the test.
```

Alert level C

```
PLAT213_ALERT_2_C Atom C34             has ADP max/min Ratio .....         3.2 prolat
PLAT222_ALERT_3_C NonSolvent Resd 1  H   Uiso(max)/Uiso(min) Range         6.4 Ratio
PLAT230_ALERT_2_C Hirshfeld Test Diff for    Si1      --C43      .         5.5 s.u.
PLAT230_ALERT_2_C Hirshfeld Test Diff for    F10      --C44      .         6.3 s.u.
PLAT245_ALERT_2_C U(iso) H28A      Smaller than U(eq) C28       by       0.022 Ang**2
PLAT340_ALERT_3_C Low Bond Precision on  C-C Bonds ...............      0.0067 Ang.
PLAT413_ALERT_2_C Short Inter XH3 .. XHn     H34A      ..H37A      .        2.08 Ang.
                                           -1/2+x,3/2-y,1-z  =      4_466 Check
PLAT420_ALERT_2_C D-H Bond Without Acceptor  N1       --H1       .      Please Check
```

Alert level G

```
PLAT242_ALERT_2_G Low    'MainMol' Ueq as Compared to Neighbors of         C42 Check
PLAT242_ALERT_2_G Low    'MainMol' Ueq as Compared to Neighbors of         C44 Check
PLAT242_ALERT_2_G Low    'MainMol' Ueq as Compared to Neighbors of         C46 Check
PLAT343_ALERT_2_G Unusual sp3      Angle Range in Main Residue for         C41 Check
PLAT434_ALERT_2_G Short Inter HL..HL Contact F4       ..F14               2.77 Ang.
                                           1-x,-1/2+y,1/2-z  =      3_645 Check
PLAT941_ALERT_3_G Average HKL Measurement Multiplicity ...........         3.4 Low
```

```
   0 ALERT level A = Most likely a serious problem - resolve or explain
   0 ALERT level B = A potentially serious problem, consider carefully
   8 ALERT level C = Check. Ensure it is not caused by an omission or oversight
   6 ALERT level G = General information/check it is not something unexpected

   0 ALERT type 1 CIF construction/syntax error, inconsistent or missing data
  11 ALERT type 2 Indicator that the structure model may be wrong or deficient
   3 ALERT type 3 Indicator that the structure quality may be low
   0 ALERT type 4 Improvement, methodology, query or suggestion
   0 ALERT type 5 Informative message, check
```

Validation response form

Please find below a validation response form (VRF) that can be filled in and pasted into your CIF.

```
# start Validation Reply Form
_vrf_PLAT213_compound1b
;
PROBLEM: Atom C34             has ADP max/min Ratio .....         3.2 prolat
RESPONSE: ...
;
_vrf_PLAT222_compound1b
;
PROBLEM: NonSolvent Resd 1  H   Uiso(max)/Uiso(min) Range         6.4 Ratio
RESPONSE: ...
;
_vrf_PLAT230_compound1b
```

```
;
PROBLEM: Hirshfeld Test Diff for    Si1      --C43      .        5.5 s.u.
RESPONSE: ...
;
_vrf_PLAT245_compound1b
;
PROBLEM: U(iso) H28A      Smaller than U(eq) C28       by      0.022 Ang**2
RESPONSE: ...
;
_vrf_PLAT340_compound1b
;
PROBLEM: Low Bond Precision on  C-C Bonds ..............     0.0067 Ang.
RESPONSE: ...
;
_vrf_PLAT413_compound1b
;
PROBLEM: Short Inter XH3 .. XHn     H34A     ..H37A     .       2.08 Ang.
RESPONSE: ...
;
_vrf_PLAT420_compound1b
;
PROBLEM: D-H Bond Without Acceptor  N1       --H1       .     Please Check
RESPONSE: ...
;
# end Validation Reply Form
```

It is advisable to attempt to resolve as many as possible of the alerts in all categories. Often the minor alerts point to easily fixed oversights, errors and omissions in your CIF or refinement strategy, so attention to these fine details can be worthwhile. In order to resolve some of the more serious problems it may be necessary to carry out additional measurements or structure refinements. However, the purpose of your study may justify the reported deviations and the more serious of these should normally be commented upon in the discussion or experimental section of a paper or in the "special_details" fields of the CIF. checkCIF was carefully designed to identify outliers and unusual parameters, but every test has its limitations and alerts that are not important in a particular case may appear. Conversely, the absence of alerts does not guarantee there are no aspects of the results needing attention. It is up to the individual to critically assess their own results and, if necessary, seek expert advice.

Publication of your CIF in IUCr journals

A basic structural check has been run on your CIF. These basic checks will be run on all CIFs submitted for publication in IUCr journals (*Acta Crystallographica*, *Journal of Applied Crystallography*, *Journal of Synchrotron Radiation*); however, if you intend to submit to *Acta Crystallographica Section C* or *E* or *IUCrData*, you should make sure that full publication checks are run on the final version of your CIF prior to submission.

Publication of your CIF in other journals

Please refer to the *Notes for Authors* of the relevant journal for any special instructions relating to CIF submission.

PLATON version of 13/07/2021; check.def file version of 13/07/2021

Datablock compound1b - ellipsoid plot

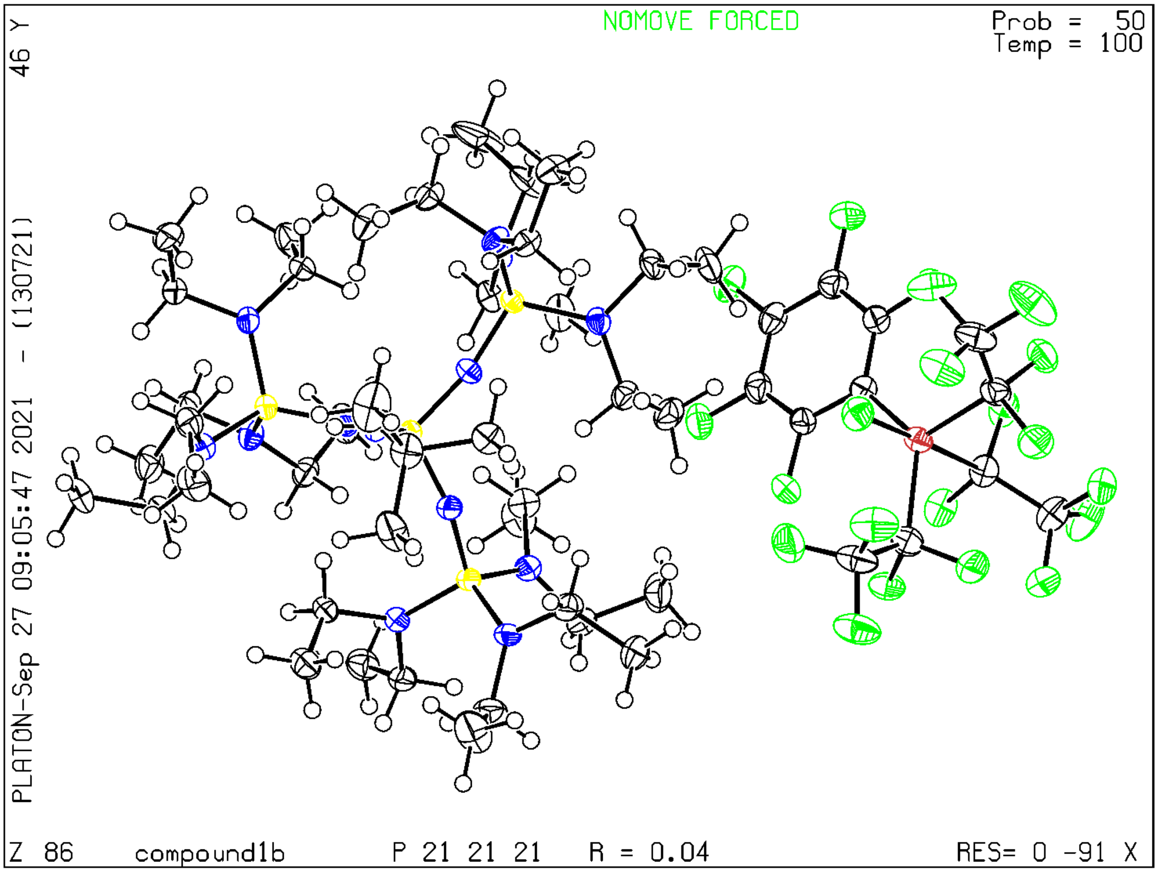

Supplement: Supplementary file 2 — Supporting Information [file ANIE-61-0-s005.pdf]
