# Supplementary material for: Oxidative Additions of C−F Bonds to the Silanide Anion [Si(C2F5)3]−
Source: Angew Chem Int Ed Engl. 2022 Feb 28;61(17):e202116468. doi: 10.1002/anie.202116468 (PMC9310575; doi:10.1002/anie.202116468)

checkCIF/PLATON report

You have not supplied any structure factors. As a result the full set of tests cannot be run.

THIS REPORT IS FOR GUIDANCE ONLY. IF USED AS PART OF A REVIEW PROCEDURE FOR PUBLICATION, IT SHOULD NOT REPLACE THE EXPERTISE OF AN EXPERIENCED CRYSTALLOGRAPHIC REFEREE.

No syntax errors found. CIF dictionary Interpreting this report

Datablock: compound1d

Bond precision: C-C = 0.0021 A Wavelength=1.54184

Cell:	a=11.7988(3)	b=16.5318(5)	c=17.9047(5)
	alpha=88.296(2)	beta=83.625(2)	gamma=86.104(2)
Temperature:	100 K		

	Calculated	Reported
Volume	3461.93(17)	3461.91(16)
Space group	P -1	P -1
Hall group	-P 1	-P 1
Moiety formula	C40 H100 N13 P4, C11 F20 N Si	C40 H100 N13 P4, C11 F20 N Si
Sum formula	C51 H100 F20 N14 P4 Si	C51 H100 F20 N14 P4 Si
Mr	1441.42	1441.41
Dx,g cm-3	1.383	1.383
Z	2	2
Mu (mm-1)	2.052	2.052
F000	1516.0	1516.0
F000'	1524.24	
h,k,lmax	14,20,22	14,20,22
Nref	14562	14216
Tmin,Tmax	0.692,0.750	0.710,1.000
Tmin'	0.628	

Correction method= # Reported T Limits: Tmin=0.710 Tmax=1.000
AbsCorr = GAUSSIAN

Data completeness= 0.976 Theta(max)= 76.548

R(reflections)= 0.0324(12558) wR2(reflections)= 0.0834(14216)

S = 1.019 Npar= 872

The following ALERTS were generated. Each ALERT has the format
test-name_ALERT_alert-type_alert-level.
Click on the hyperlinks for more details of the test.

Alert level C

```
PLAT230_ALERT_2_C Hirshfeld Test Diff for    Si1      --C47      .        5.7 s.u.
PLAT420_ALERT_2_C D-H Bond Without Acceptor  N1       --H1       .    Please Check
PLAT601_ALERT_2_C Unit Cell Contains Solvent Accessible VOIDS of .         37 Ang**3
```

Alert level G

```
PLAT003_ALERT_2_G Number of Uiso or Uij Restrained non-H Atoms ...          2 Report
PLAT154_ALERT_1_G The s.u.'s on the Cell Angles are Equal ..(Note)      0.002 Degree
PLAT171_ALERT_4_G The CIF-Embedded .res File Contains EADP Records          4 Report
PLAT178_ALERT_4_G The CIF-Embedded .res File Contains SIMU Records          1 Report
PLAT242_ALERT_2_G Low    'MainMol' Ueq as Compared to Neighbors of        C44 Check
PLAT242_ALERT_2_G Low    'MainMol' Ueq as Compared to Neighbors of        C46 Check
PLAT301_ALERT_3_G Main Residue  Disorder ..............(Resd  1  )        11% Note
PLAT410_ALERT_2_G Short Intra H...H Contact  H5B      ..H31B     .       2.13 Ang.
                                                      x,y,z  =      1_555 Check
PLAT412_ALERT_2_G Short Intra XH3 .. XHn     H21B     ..H24F     .       2.12 Ang.
                                                      x,y,z  =      1_555 Check
PLAT412_ALERT_2_G Short Intra XH3 .. XHn     H26A     ..H23D     .       2.10 Ang.
                                                      x,y,z  =      1_555 Check
PLAT412_ALERT_2_G Short Intra XH3 .. XHn     H30A     ..H32C     .       2.05 Ang.
                                                      x,y,z  =      1_555 Check
PLAT412_ALERT_2_G Short Intra XH3 .. XHn     H37A     ..H24D     .       2.01 Ang.
                                                      x,y,z  =      1_555 Check
PLAT412_ALERT_2_G Short Intra XH3 .. XHn     H38A     ..H39B     .       2.10 Ang.
                                                      x,y,z  =      1_555 Check
PLAT860_ALERT_3_G Number of Least-Squares Restraints ............           6 Note
PLAT941_ALERT_3_G Average HKL Measurement Multiplicity ..........         2.2 Low
```

```
   0 ALERT level A = Most likely a serious problem - resolve or explain
   0 ALERT level B = A potentially serious problem, consider carefully
   3 ALERT level C = Check. Ensure it is not caused by an omission or oversight
  15 ALERT level G = General information/check it is not something unexpected

   1 ALERT type 1 CIF construction/syntax error, inconsistent or missing data
  12 ALERT type 2 Indicator that the structure model may be wrong or deficient
   3 ALERT type 3 Indicator that the structure quality may be low
   2 ALERT type 4 Improvement, methodology, query or suggestion
   0 ALERT type 5 Informative message, check
```

Validation response form

Please find below a validation response form (VRF) that can be filled in and pasted into your CIF.

```
# start Validation Reply Form
_vrf_PLAT230_compound1d
;
PROBLEM: Hirshfeld Test Diff for    Si1      --C47      .        5.7 s.u.
```

```
RESPONSE: ...
;
_vrf_PLAT420_compound1d
;
PROBLEM: D-H Bond Without Acceptor  N1       --H1       .     Please Check
RESPONSE: ...
;
_vrf_PLAT601_compound1d
;
PROBLEM: Unit Cell Contains Solvent Accessible VOIDS of .         37 Ang**3
RESPONSE: ...
;
# end Validation Reply Form
```

It is advisable to attempt to resolve as many as possible of the alerts in all categories. Often the minor alerts point to easily fixed oversights, errors and omissions in your CIF or refinement strategy, so attention to these fine details can be worthwhile. In order to resolve some of the more serious problems it may be necessary to carry out additional measurements or structure refinements. However, the purpose of your study may justify the reported deviations and the more serious of these should normally be commented upon in the discussion or experimental section of a paper or in the "special_details" fields of the CIF. checkCIF was carefully designed to identify outliers and unusual parameters, but every test has its limitations and alerts that are not important in a particular case may appear. Conversely, the absence of alerts does not guarantee there are no aspects of the results needing attention. It is up to the individual to critically assess their own results and, if necessary, seek expert advice.

Publication of your CIF in IUCr journals

A basic structural check has been run on your CIF. These basic checks will be run on all CIFs submitted for publication in IUCr journals (*Acta Crystallographica*, *Journal of Applied Crystallography*, *Journal of Synchrotron Radiation*); however, if you intend to submit to *Acta Crystallographica Section C* or *E* or *IUCrData*, you should make sure that full publication checks are run on the final version of your CIF prior to submission.

Publication of your CIF in other journals

Please refer to the *Notes for Authors* of the relevant journal for any special instructions relating to CIF submission.

PLATON version of 13/07/2021; check.def file version of 13/07/2021

Datablock compound1d - ellipsoid plot

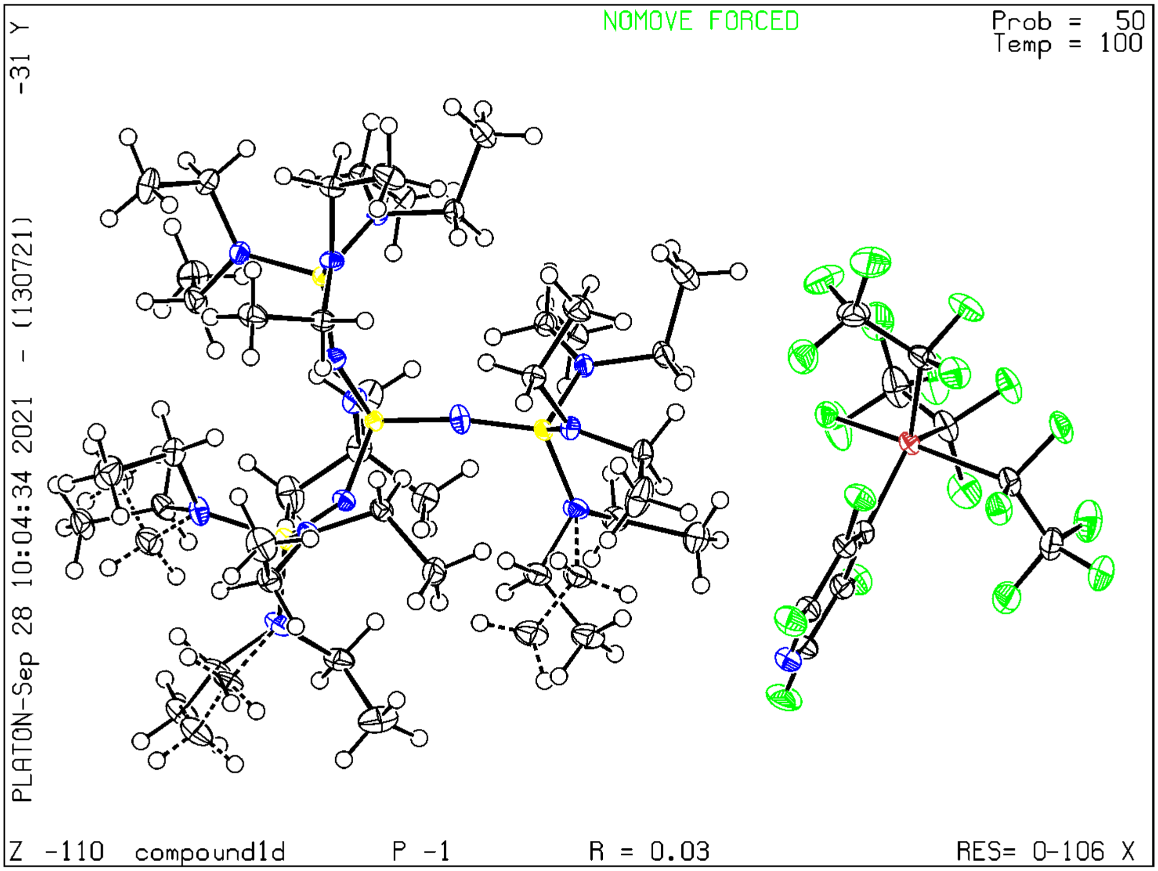

Supplement: Supplementary file 4 — Supporting Information [file ANIE-61-0-s009.pdf]
