# Supplementary material for: Oxidative Additions of C−F Bonds to the Silanide Anion [Si(C2F5)3]−
Source: Angew Chem Int Ed Engl. 2022 Feb 28;61(17):e202116468. doi: 10.1002/anie.202116468 (PMC9310575; doi:10.1002/anie.202116468)

checkCIF/PLATON report

You have not supplied any structure factors. As a result the full set of tests cannot be run.

THIS REPORT IS FOR GUIDANCE ONLY. IF USED AS PART OF A REVIEW PROCEDURE FOR PUBLICATION, IT SHOULD NOT REPLACE THE EXPERTISE OF AN EXPERIENCED CRYSTALLOGRAPHIC REFEREE.

No syntax errors found. CIF dictionary Interpreting this report

Datablock: compound1e

```
Bond precision:   C-C = 0.0109 A          Wavelength=1.54184

Cell:             a=13.6150(5)     b=15.8534(7)       c=16.3232(7)
                  alpha=90         beta=94.701(4)     gamma=90
Temperature:      100 K

                 Calculated                       Reported
Volume           3511.4(3)                        3511.4(3)
Space group      P 21                             P 1 21 1
Hall group       P 2yb                            P 2yb
Moiety formula   C11 F23 Si, C40 H100 N13 P4      C40 H100 N13 P4, C11 F23 Si
Sum formula      C51 H100 F23 N13 P4 Si           C51 H100 F23 N13 P4 Si
Mr               1484.41                          1484.40
Dx,g cm-3        1.404                            1.404
Z                2                                2
Mu (mm-1)        2.099                            2.098
F000             1556.0                           1556.0
F000'            1564.62
h,k,lmax         17,20,20                         16,19,20
Nref             14780[ 7667]                     14367
Tmin,Tmax        0.777,0.925                      0.742,1.000
Tmin'            0.694

Correction method= # Reported T Limits: Tmin=0.742 Tmax=1.000
AbsCorr = GAUSSIAN

Data completeness= 1.87/0.97       Theta(max)= 76.620

R(reflections)= 0.0543( 12206)                         wR2(reflections)=
                                                       0.1585( 14367)
S = 1.072                  Npar= 1152
```

The following ALERTS were generated. Each ALERT has the format
test-name_ALERT_alert-type_alert-level.
Click on the hyperlinks for more details of the test.

Alert level B

```
PLAT340_ALERT_3_B Low Bond Precision on  C-C Bonds ..............    0.01088 Ang.
```

Author Response: Caused by many disordered carbon atoms and acentric space group.

Alert level C

```
STRVA01_ALERT_4_C          Flack test results are ambiguous.
           From the CIF: _refine_ls_abs_structure_Flack    0.380
           From the CIF: _refine_ls_abs_structure_Flack_su    0.030
PLAT089_ALERT_3_C Poor Data / Parameter Ratio (Zmax < 18) ........       6.66 Note
PLAT420_ALERT_2_C D-H Bond Without Acceptor  N1       --H1       .     Please Check
```

Alert level G

```
PLAT002_ALERT_2_G Number of Distance or Angle Restraints on AtSite         27 Note
PLAT003_ALERT_2_G Number of Uiso or Uij Restrained non-H Atoms ...         39 Report
PLAT033_ALERT_4_G Flack x Value Deviates > 3.0 * sigma from Zero .      0.380 Note
PLAT042_ALERT_1_G Calc. and Reported Moiety Formula Strings Differ     Please Check
PLAT171_ALERT_4_G The CIF-Embedded .res File Contains EADP Records          8 Report
PLAT172_ALERT_4_G The CIF-Embedded .res File Contains DFIX Records          1 Report
PLAT176_ALERT_4_G The CIF-Embedded .res File Contains SADI Records         11 Report
PLAT178_ALERT_4_G The CIF-Embedded .res File Contains SIMU Records          2 Report
PLAT187_ALERT_4_G The CIF-Embedded .res File Contains RIGU Records          2 Report
PLAT230_ALERT_2_G Hirshfeld Test Diff for    F16A     --C46      .        8.0 s.u.
PLAT242_ALERT_2_G Low    'MainMol' Ueq as Compared to Neighbors of       C42A Check
PLAT301_ALERT_3_G Main Residue  Disorder .............(Resd  1  )         89% Note
PLAT301_ALERT_3_G Main Residue  Disorder .............(Resd  2  )         14% Note
PLAT412_ALERT_2_G Short Intra XH3 .. XHn     H25B     ..H28F     .       2.07 Ang.
                                                      x,y,z  =     1_555 Check
PLAT412_ALERT_2_G Short Intra XH3 .. XHn     H26C     ..H27D     .       2.03 Ang.
                                                      x,y,z  =     1_555 Check
PLAT412_ALERT_2_G Short Intra XH3 .. XHn     H36B     ..H28A     .       2.01 Ang.
                                                      x,y,z  =     1_555 Check
PLAT811_ALERT_5_G No ADDSYM Analysis: Too Many Excluded Atoms ....          ! Info
PLAT860_ALERT_3_G Number of Least-Squares Restraints ............         561 Note
PLAT933_ALERT_2_G Number of OMIT Records in Embedded .res File ...          4 Note
PLAT941_ALERT_3_G Average HKL Measurement Multiplicity ..........         4.3 Low
```

---

   0 **ALERT level A** = Most likely a serious problem - resolve or explain
   1 **ALERT level B** = A potentially serious problem, consider carefully
   3 **ALERT level C** = Check. Ensure it is not caused by an omission or oversight
  20 **ALERT level G** = General information/check it is not something unexpected

   1 ALERT type 1 CIF construction/syntax error, inconsistent or missing data

```
   9 ALERT type 2 Indicator that the structure model may be wrong or deficient
   6 ALERT type 3 Indicator that the structure quality may be low
   7 ALERT type 4 Improvement, methodology, query or suggestion
   1 ALERT type 5 Informative message, check
```

Validation response form

Please find below a validation response form (VRF) that can be filled in and pasted into your CIF.

```
# start Validation Reply Form
_vrf_STRVA01_compound1e
;
PROBLEM: Flack test results are ambiguous.
RESPONSE: ...
;
_vrf_PLAT089_compound1e
;
PROBLEM: Poor Data / Parameter Ratio (Zmax < 18) ........       6.66 Note
RESPONSE: ...
;
_vrf_PLAT420_compound1e
;
PROBLEM: D-H Bond Without Acceptor  N1       --H1       .     Please Check
RESPONSE: ...
;
# end Validation Reply Form
```

It is advisable to attempt to resolve as many as possible of the alerts in all categories. Often the minor alerts point to easily fixed oversights, errors and omissions in your CIF or refinement strategy, so attention to these fine details can be worthwhile. In order to resolve some of the more serious problems it may be necessary to carry out additional measurements or structure refinements. However, the purpose of your study may justify the reported deviations and the more serious of these should normally be commented upon in the discussion or experimental section of a paper or in the "special_details" fields of the CIF. checkCIF was carefully designed to identify outliers and unusual parameters, but every test has its limitations and alerts that are not important in a particular case may appear. Conversely, the absence of alerts does not guarantee there are no aspects of the results needing attention. It is up to the individual to critically assess their own results and, if necessary, seek expert advice.

Publication of your CIF in IUCr journals

A basic structural check has been run on your CIF. These basic checks will be run on all CIFs submitted for publication in IUCr journals (*Acta Crystallographica*, *Journal of Applied Crystallography*, *Journal of Synchrotron Radiation*); however, if you intend to submit to *Acta Crystallographica Section C* or *E* or *IUCrData*, you should make sure that full publication checks are run on the final version of your CIF prior to submission.

Publication of your CIF in other journals

Please refer to the *Notes for Authors* of the relevant journal for any special instructions relating to CIF submission.

PLATON version of 13/07/2021; check.def file version of 13/07/2021

Datablock compound1e - ellipsoid plot

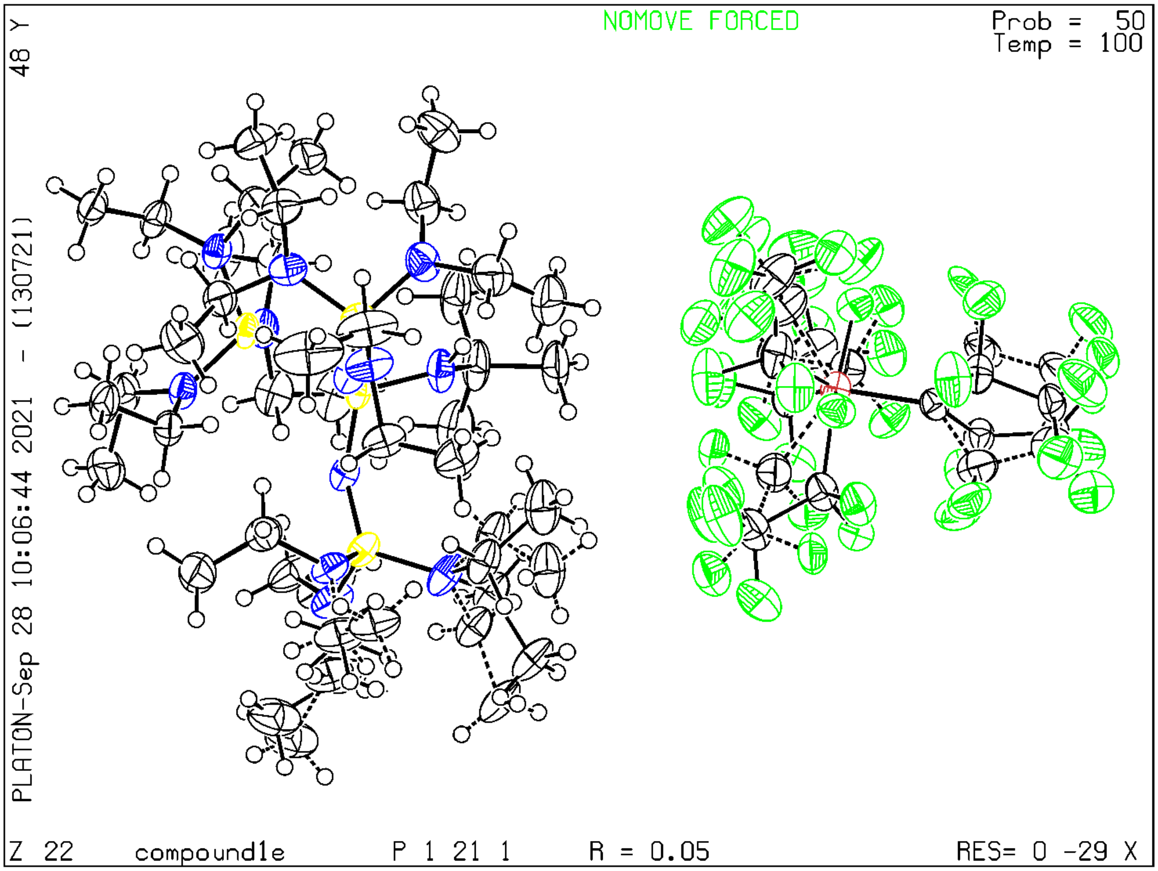

Supplement: Supplementary file 5 — Supporting Information [file ANIE-61-0-s006.pdf]
