# Supplementary material for: Oxidative Additions of C−F Bonds to the Silanide Anion [Si(C2F5)3]−
Source: Angew Chem Int Ed Engl. 2022 Feb 28;61(17):e202116468. doi: 10.1002/anie.202116468 (PMC9310575; doi:10.1002/anie.202116468)
Supplement: Supplementary file 11 — Supporting Information [file ANIE-61-0-s002.pdf]

Angewandte Chemie
Eine Zeitschrift der Gesellschaft Deutscher Chemiker

Supporting Information

Oxidative Additions of C–F Bonds to the Silanide Anion $[Si(C_2F_5)_3]^-$

*N. Tiessen, M. Keßler, B. Neumann, H.-G. Stammler, B. Hoge**

Table of Contents

1 **Experimental Procedures**
  1.1 General Part
  1.2 Syntheses
2 **NMR Spectra**
  2.1 NMR spectra of $[EtP_4H][Si(C_2F_5)_3(C_7F_7)F]$ (**1a**)
  2.2 NMR spectra of $[EtP_4H][Si(C_2F_5)_3(C_6F_5)F]$ (**1b**)
  2.3 NMR spectra of $[EtP_4H][Si(C_2F_5)_3(C_{10}F_7)F]$ (**1c**)
  2.4 NMR spectra of $[EtP_4H][Si(C_2F_5)_3(C_5F_4N)F]$ (**1d**)
  2.5 NMR spectra of $[EtP_4H][Si(C_2F_5)_3(C_5F_7)F]$ (**1e**)
  2.6 NMR spectra of $[EtP_4H][Si(C_2F_5)_3(C_3F_5)F]$ (**1f**)
3 **X-Ray Data**
4 **DFT calculations**

1 Experimental Procedures

1.1 General Part

All reactions were performed in the absence of water and air by use of standard *Schlenk* techniques. Solvents were dried according to known procedures. $Si(C_2F_5)_3H$ and $[EtP_4H][Si(C_2F_5)_3]$ were synthesized as described in the literature.[1]
NMR spectra were either recorded on a *Bruker Avance III 300* or *Bruker Avance III 500 HD* in the indicated solvent. Positive shifts are downfield from the external standards (TMS for ^{1}H, ^{13}C and ^{29}Si, H_3PO_4 for ^{31}P, CCl_3F for ^{19}F). IR spectroscopic measurements were performed on a *Bruker Alpha-FT-IR* spectrometer with a diamond crystal.
ESI mass spectra were recorded on a ZQ2000 single quadrupole mass spectrometer (Waters, Manchester, UK) equipped with an ESI source, operating with a spray voltage of 3.5 kV. Nitrogen served both as the nebulizer gas and the dry gas and was generated by a nitrogen generator NGM 11. The mass axis was externally calibrated with ESI-L Tuning Mix (Agilent Technologies, Santa Clara, CA, USA) as calibration standard. Melting points were measured on a *Mettler Toledo Mp70* Melting Point System. Elemental analyses were performed by Mikroanalytisches Laboratorium Kolbe (Oberhausen, Germany). SCXRD was performed on a Rigaku Supernova diffractometer.

1.2 Syntheses

General procedure for the synthesis of Tris(pentafluoroethyl)perfluoroorganylfluorosilicates

A solution of $[EtP_4H][Si(C_2F_5)_3]$ in Et_2O was treated with the respective perfluoroorganyl compound at room temperature. After the conversion was completed, all volatile compounds were removed in vacuo and the residue was washed with *n*-pentane (3 x 10 mL). The corresponding fluorosilicates were isolated as colorless solids.

$[EtP_4H][Si(C_2F_5)_3(C_7F_7)F]$ (1a): $[EtP_4H][Si(C_2F_5)_3]$ (0.46 g, 0.36 mmol) and octafluorotoluene (85 mg, 0.36 mmol) afforded $[EtP_4H][Si(C_2F_5)_3(C_7F_7)F]$ (0.52 mg, 0.34 mmol) in a 93 % yield.

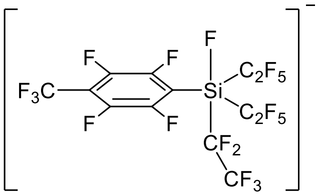

the cation is not depicted and only one possible isomer is shown

^1H NMR (300.1 MHz, CD_2Cl_2, rt): δ = 3.14 (dq, $^3J_{P,H}$ = 10 Hz, $^3J_{H,H}$ = 7 Hz, 36 H, $[(CH_3C\mathbf{H}_2)_2N]_3P{=}N$-), 2.04 (d, $^2J_{P,H}$ = 7 Hz, 1 H, -P=N(**H**)^{t}Bu), 1.29 (s, 9 H, $C(C\mathbf{H}_3)_3$), 1.09 ppm (t, $^3J_{H,H}$ = 7 Hz, 54 H, $[(C\mathbf{H}_3CH_2)_2N]_3P{=}N$-);

$^{13}C\{^1H\}$ NMR (125.8 MHz, CD_2Cl_2, rt): δ = 51.0 (d, $^2J_{C,P}$ = 4 Hz, -P=N(H)**C**$(CH_3)_3$), 39.5 (d, $^2J_{C,P}$ = 6 Hz, $[(CH_3\mathbf{C}H_2)_2N]_3P{=}N$-), 31.7 (d, $^3J_{C,P}$ = 5 Hz, -P=N(H)C$(\mathbf{C}H_3)_3$), 13.7 ppm (d, $^3J_{C,P}$ = 4 Hz, $[(\mathbf{C}H_3CH_2)_2N]_3P{=}N$-);

$^{13}C\{^{19}F\}$ NMR (75.5 MHz, CD_2Cl_2, rt): δ = 147.0 (s, *ortho*-C to Si), 144.6 (s, *meta*-C to Si), 142.9 (s, $F_3C\mathbf{C}_{Ar}$), 125.8 (s, -$CF_2\mathbf{C}F_3$), 121.8 (s, $F_3\mathbf{C}C_{Ar}$), 108.7 ppm (s, *ipso*-C_{Ar} to Si); *the signal for* $\mathbf{C}F_2CF_3$ *is overlapped with the signal at 121.8 ppm. It was detected by a* $^{13}C\{^{19}F\}$*DEPT135 NMR spectrum*;

^{19}F NMR (470.7 MHz, CD_2Cl_2 rt): δ = −56.9 (t, $^4J_{F,F}$ = 21 Hz, 3 F, $\mathbf{F}_3CC_{Ar}$), −81.6 (s, 9 F, -$CF_2C\mathbf{F}_3$), −121.8 (s, br, 6 F, -$C\mathbf{F}_2CF_3$), −122.7 (s, br, 2 F, *ortho*-CF to Si), −142.8 (m, 2 F, *meta*-CF to Si);

^{19}F NMR (470.7 MHz, CD_2Cl_2, 193 K): δ = −53.5 (s, br, 1 F, Si**F**), −56.6 (t, $^4J_{F,F}$ = 21 Hz, 3 F, $C_{Ar}C\mathbf{F}_3$), −80.0 (s, 3 F, $CF_2C\mathbf{F}_3$); −83.3 (s, 6 F, $CF_2C\mathbf{F}_3$), −119.5 (s, br, 2 F, $C\mathbf{F}_2CF_3$), −122.1 to −125.9 (AB system, 4 F, $C\mathbf{F}_2CF_3$), −123.2 (s, br, 2 F, *ortho*-CF to Si), −141.7 (m, 2 F, *meta*-CF to Si);

$^{29}Si\{^1H\}$ IG (99.4 MHz, CD_2Cl_2, rt): δ = −104.8 ppm (dm, $^1J_{Si,F}$ = 296 Hz, Si);

^{31}P NMR (202.5 MHz, CD_2Cl_2, rt): δ = 7.6 (dm, $^2J_{P,P}$ = 70, $^3J_{P,H}$ = 10 Hz, 3 P, $[(Et_2N)_3\mathbf{P}{=}N]_3PN(H)^tBu$), −33.8 ppm (qd, $^2J_{P,P}$ = 70, $^2J_{P,H}$ = 7 Hz, 1 P, $[(Et_2N)_3P{=}N]_3\mathbf{P}N(H)^tBu$));

IR (ATR): $\tilde{\nu}$ = 2973 (w), 2938 (w), 2874 (w), 1455 (m), 1418 (w), 1379 (m), 1353 (m), 1317 (m), 1275 (s, br), 1200 (s), 1172 (vs), 1138 (s), 1107 (m), 1094 (m), 1056 (m), 1018 (vs), 966 (m), 942 (vs), 924 (s), 843 (m), 793 (s), 741 (m), 696 (s), 610 (m), 592 (m), 510 (s), 476 (s), 451 (s), 403 (s) cm^{-1} (m);

MS (ESI, pos., ACN): *m/z* (%): 887 (100) $[EtP_4H]^+$; MS (ESI, neg., ACN): *m/z* (%): 621 (100) $[Si(C_2F_5)_3(C_7F_7)F]^-$, 423 (2) $[Si(C_2F_5)_3F_2]^-$, 301 (3) $[Si(C_2F_5)_2FO]^-$;

Elemental analysis calcd. (%) for $C_{53}H_{100}F_{23}N_{13}P_4Si$: C 42.20, H 6.68, N 12.07, Si 1.86, F 28.97; found: C 42.02, H 6.59, N 11.89, Si 2.03, F 28.81.

$[EtP_4H][Si(C_2F_5)_3(C_6F_5)F]$ (1b): $[EtP_4H][Si(C_2F_5)_3]$ (0.44 g, 0.35 mmol) and hexafluorobenzene (65 mg, 0.35 mmol) afforded $[EtP_4H][Si(C_2F_5)_3(C_6F_5)F]$ (0.45 mg, 0.31 mmol) in an 89 % yield. The reaction mixture was stirred for 11 days at room temperature until full conversion.

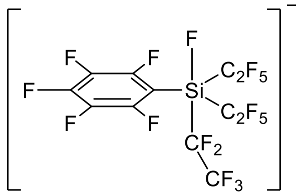

the cation is not depicted and only one possible isomer is shown

^{1}H NMR (300.1 MHz, CD_2Cl_2, rt): δ = 3.14 (dq, $^{3}J_{P,H}$ = 10 Hz, $^{3}J_{H,H}$ = 7 Hz, 36 H, $[(CH_3C\mathbf{H}_2)_2N]_3P{=}N$-), 2.04 (d, $^{2}J_{P,H}$ = 7 Hz, 1 H, -P=N(**H**)^{t}Bu), 1.29 (s, 9 H, $C(C\mathbf{H}_3)_3$), 1.10 ppm (t, $^{3}J_{H,H}$ = 7 Hz, 54 H, $[(C\mathbf{H}_3CH_2)_2N]_3P{=}N$-);

^{13}C{^{1}H} NMR (125.8 MHz, CD_2Cl_2, rt): δ = 51.1 (d, $^{2}J_{C,P}$ = 4 Hz, -P=N(H)**C**$(CH_3)_3$), 39.5 (d, $^{2}J_{C,P}$ = 6 Hz, $[(CH_3\mathbf{C}H_2)_2N]_3P{=}N$-), 31.7 (d, $^{3}J_{C,P}$ = 5 Hz, -P=N(H)C$(\mathbf{C}H_3)_3$), 13.7 ppm (d, $^{3}J_{C,P}$ = 4 Hz, $[(\mathbf{C}H_3CH_2)_2N]_3P{=}N$-);

^{13}C{^{19}F} NMR (75.5 MHz, CD_2Cl_2, rt): δ = 146.4 (s, *ortho*-C), 140.6 (s, *para*-C), 136.7 (s, *meta*-C), 121.4 (s, $-CF_2\mathbf{C}F_3$), 113.8 ppm (s, *ipso*-C); ***C**F_2 could not be detected;*

^{19}F NMR (470.7 MHz, CD_2Cl_2 rt): δ = −81.4 (s, 9 F, $-CF_2C\mathbf{F}_3$), −121.9 (s, br, 6 F, $-C\mathbf{F}_2CF_3$), −124.0 (s, br, 2 F, *ortho*-CF), −157.2 (t, $^{3}J_{F,F}$ = 20 Hz, 1 F, *para*-CF), −163.9 ppm (dm, $^{3}J_{F,F}$ = 20 Hz, 2 F, *meta*-CF);

^{29}Si{^{1}H} IG (99.4 MHz, CD_2Cl_2, rt): δ = −104.6 ppm (dm, $^{1}J_{Si,F}$ = 294 Hz, Si);

^{31}P NMR (202.5 MHz, CD_2Cl_2, rt): δ = 7.6 (dm, $^{2}J_{P,P}$ = 70, $^{3}J_{P,H}$ = 10 Hz, 3 P, $[(Et_2N)_3\mathbf{P}{=}N]_3PN(H)^tBu$), −33.8 ppm (qd, $^{2}J_{P,P}$ = 70, $^{2}J_{P,H}$ = 7 Hz, 1 P, $[(Et_2N)_3P{=}N]_3\mathbf{P}N(H)^tBu$));

IR (ATR): $\tilde{\nu}$ = 2975 (w), 2936 (w), 2872 (w), 1644 (vw), 1512 (w), 1465 (m), 1414 (w), 1379 (m), 1351 (m), 1283 (s, br), 1202 (s), 1174 (vs), 1129 (s), 1056 (m), 1017 (vs), 968 (s), 944 (vs), 848 (m), 793 (m), 740 (m), 695 (s), 609 (m), 591 (m), 509 (s), 496 (s), 469 (s), 427 cm^{-1} (s);

MS (ESI, pos., ACN): *m/z* (%): 887 (100) $[EtP_4H]^+$; MS (ESI, neg., ACN): *m/z* (%): 671 (6) $[Si(C_2F_5)_4(C_6F_5)]^-$, 571 (100) $[Si(C_2F_5)_3(C_6F_5)F]^-$, 201 (1) $[Si(C_2F_5)F_2O]^-$;

Elemental analysis calcd. (%) for $C_{52}H_{100}F_{21}N_{13}P_4Si$: C 42.83, H 6.91, N 12.49, Si 1.93, F 27.36; found: C 42.84, H 6.90, N 12.47, Si 1.92, F 27.32.

$[EtP_4H][Si(C_2F_5)_3(C_{10}F_7)F]$ (1c): $[EtP_4H][Si(C_2F_5)_3]$ (0.44 g, 0.35 mmol) and octafluoronaphthalene (106 mg, 0.39 mmol) afforded $[EtP_4H][Si(C_2F_5)_3(C_{10}F_7)F]$ (0.54 mg, 0.35 mmol) in a quantitative yield.

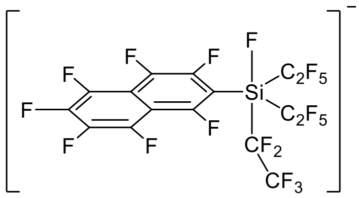

the cation is not depicted and only one possible isomer is shown

^{1}H NMR (300.1 MHz, CD_2Cl_2, rt): δ = 3.14 (dq, $^{3}J_{P,H}$ = 10 Hz, $^{3}J_{H,H}$ = 7 Hz, 36 H, $[(CH_3C\mathbf{H}_2)_2N]_3P{=}N$-), 2.04 (d, $^{2}J_{P,H}$ = 7 Hz, 1 H, -P=N(**H**)^{t}Bu), 1.28 (s, 9 H, $C(C\mathbf{H}_3)_3$), 1.09 ppm (t, $^{3}J_{H,H}$ = 7 Hz, 54 H, $[(C\mathbf{H}_3CH_2)_2N]_3P{=}N$-);

^{13}C{^{1}H} NMR (125.8 MHz, CD_2Cl_2, rt): δ = 51.1 (d, $^{2}J_{C,P}$ = 4 Hz, P=N(H)**C**$(CH_3)_3$), 39.5 (d, $^{2}J_{C,P}$ = 6 Hz, $[(CH_3\mathbf{C}H_2)_2N]_3P{=}N$-), 31.7 (d, $^{3}J_{C,P}$ = 5 Hz, -P=N(H)C$(\mathbf{C}H_3)_3$), 13.7 ppm (d, $^{3}J_{C,P}$ = 4 Hz, $[(\mathbf{C}H_3CH_2)_2N]_3P{=}N$-);

^{13}C{^{19}F} NMR (75.5 MHz, CD_2Cl_2, rt): δ = 153.0 (s), 149.3 (s), 142.0 (s), 141.3 (s), 140.1 (s), 139.7 (s), 122.0 (s), 121.9 (s), 120.0 (s), 112.2 (s), 108.4 ppm (s);

^{19}F NMR (470.7 MHz, CD_2Cl_2 rt): δ = −81.4 (s, 9 F, $CF_2C\mathbf{F}_3$), −100.4 (dm, $^{3}J_{F,F}$ = 71 Hz, 1 F, F_{Ar}), −119.3 (s, br, 1 F, F_{Ar}), −121.7 (s, br, 6 F, $C\mathbf{F}_2CF_3$), −144.9 (dt, 71 Hz, 17 Hz, 1 F, F_{Ar}), −148.7 (dt, 56 Hz, 17 Hz, 1 F, F_{Ar}), −153.1 (dt, 56 Hz, 20 Hz, 1 F, F_{Ar}), −156.7 (t, 19 Hz, 1 F, F_{Ar}), −159.4 ppm (t, 18 Hz, 1 F, F_{Ar});

^{29}Si{^{1}H} IG (99.4 MHz, CD_2Cl_2, rt): δ = −104.4 ppm (dm, $^{1}J_{Si,F}$ = 296 Hz, Si);

^{31}P NMR (202.5 MHz, CD_2Cl_2, rt): δ = 7.6 (dm, $^{2}J_{P,P}$ = 70, $^{3}J_{P,H}$ = 10 Hz, 3 P, $[(Et_2N)_3\mathbf{P}{=}N]_3PN(H)^tBu$), −33.8 ppm (qd, $^{2}J_{P,P}$ = 70, $^{2}J_{P,H}$ = 7 Hz, 1 P, $[(Et_2N)_3P{=}N]_3\mathbf{P}N(H)^tBu$));

IR (ATR): $\tilde{\nu}$ = 2970 (w), 2936 (w), 2872 (w), 1665 (w), 1637 (w), 1600 (w), 1519 (w), 1489 (w), 1466 (w), 1397 (m), 1378 (m), 1352 (m), 1283 (vs, br), 1201 (vs), 1171 (vs), 1146 (s), 1116 (s), 1103 (m), 1074 (s), 1056 (m), 1019 (m), 943 (vs), 891 (vs), 846 (s), 827 (m), 794 (m), 744 (s), 731 (m), 703 (m), 665 (s), 637 (w), 609 (w), 598 (m), 510 (m), 490 (vs), 473 (s), 453 (vs), 414 (s) cm^{-1};

MS (ESI, pos., ACN): *m/z* (%): 887 (100) $[EtP_4H]^+$; MS (ESI, neg., ACN): *m/z* (%): 757 (<1) $[Si(C_2F_5)_4(C_{10}F_7)]^-$, 657 (100) $[Si(C_2F_5)_3(C_{10}F_7)F]^-$, 323 (14) $[Si(C_2F_5)_2F_3]^-$, 423 (100) $[Si(C_2F_5)_3F_2]^-$, 123 (12) $[SiF_5]^-$;

Elemental analysis calcd. (%) for $C_{56}H_{100}F_{23}N_{13}P_4Si$: C 43.55, H 6.53, N 11.79, Si 1.82, F 28.29; found: C 43.32, H 6.37, N 11.54, Si 1.99, F 28.12.

[EtP_4H][$Si(C_2F_5)_3(C_5F_4N)F$] (1d): [EtP_4H][$Si(C_2F_5)_3$] (0.33 g, 0.26 mmol) and pentafluoropyridine (45 mg, 0.27 mmol) afforded [EtP_4H][$Si(C_2F_5)_3(C_5F_4N)F$] (0.38 mg, 0.26 mmol) in a quantitative yield.

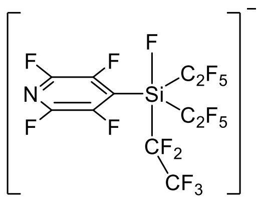

the cation is not depicted and only one possible isomer is shown

^{1}H NMR (300.1 MHz, CD_2Cl_2, rt): δ = 3.14 (dq, $^{3}J_{P,H}$ = 10 Hz, $^{3}J_{H,H}$ = 7 Hz, 36 H, [(CH_3**CH_2**$)_2N]_3P$=N-), 2.04 (d, $^{2}J_{P,H}$ = 7 Hz, 1 H, -P=N(**H**)^{t}Bu), 1.29 (s, 9 H, C(C**H_3**$)_3$), 1.09 ppm (t, $^{3}J_{H,H}$ = 7 Hz, 54 H, [(C**H_3**$CH_2)_2N]_3P$=N-);

^{13}C{^{1}H} NMR (125.8 MHz, CD_2Cl_2, rt): δ = 51.1 (d, $^{2}J_{C,P}$ = 4 Hz, P=N(H)**C**$(CH_3)_3$), 39.5 (d, $^{2}J_{C,P}$ = 6 Hz, [(CH_3**C**$H_2)_2N]_3P$=N-), 31.7 (d, $^{3}J_{C,P}$ = 5 Hz, -P=N(H)C(**C**$H_3)_3$), 13.7 ppm (d, $^{3}J_{C,P}$ = 4 Hz, [(**C**$H_3CH_2)_2N]_3P$=N-);

^{13}C{^{19}F} NMR (75.5 MHz, CD_2Cl_2, rt): δ = 143.5 (s, *meta*-C to Si), 142.4 (s, *ortho*-C to Si), 136.3 (*ipso*-C to Si), 121.7 ppm (CF_2**CF_3**), ***C**F_2CF_3 could not be detected*;

^{19}F NMR (470.7 MHz, CD_2Cl_2 rt): δ = −81.7 (s, br, 9 F, -CF_2C**F_3**), −95.6 (m, 2 F, *ortho*-CF to Si), −121.7 (s, br, 6 F, -C**F_2**CF_3), −127.5 ppm (m, 2 F, *meta*-CF to Si);

^{29}Si{^{1}H} IG (99.4 MHz, CD_2Cl_2, rt): δ = −105.0 ppm (dm, $^{1}J_{Si,F}$ = 294 Hz, Si);

^{31}P NMR (202.5 MHz, CD_2Cl_2, rt): δ = 7.6 (dm, $^{2}J_{P,P}$ = 70, $^{3}J_{P,H}$ = 10 Hz, 3 P, [$(Et_2N)_3$**P**=N$]_3$PN(H)^{t}Bu), −33.8 ppm (qd, $^{2}J_{P,P}$ = 70, $^{2}J_{P,H}$ = 7 Hz, 1 P, [$(Et_2N)_3$P=N$]_3$**P**N(H)^{t}Bu));

IR (ATR): $\tilde{\nu}$ = 2973 (w), 2935 (w), 2874 (w), 1634 (w), 1441 (m), 1424 (w), 1380 (m), 1351 (m), 1272 (s, br), 1202 (vs), 1172 (vs), 1132 (s), 1078 (m), 1067 (m), 1018 (vs), 942 (vs), 847 (w), 794 (s), 741 (m), 699 (vs), 615 (m), 593 (m), 509 (s), 496 (s), 485 (s), 461 (s), 440 (s) cm^{-1};

MS (ESI, pos., ACN): *m/z* (%): 887 (100) [EtP_4H]$^{+}$; MS (ESI, neg., ACN): *m/z* (%): 554 (100) [$Si(C_2F_5)_3(C_5F_4N)F$]$^{-}$;

Elemental analysis calcd. (%) for $C_{51}H_{100}F_{20}N_{14}P_4Si$: C 42.50, H 6.99, N 13.60, Si 1.95, F 26.36; found: C 42.44, H 7.01, N 13.58, Si 1.91, F 26.31.

[EtP_4H][$Si(C_2F_5)_3(C_5F_7)F$] (1e): [EtP_4H][$Si(C_2F_5)_3$] (0.47 g, 0.37 mmol) and octafluorocyclopentene (82 mg, 0.39 mmol) afforded [EtP_4H][$Si(C_2F_5)_3(C_5F_7)F$] (0.55 mg, 0.37 mmol) in a quantitative yield.

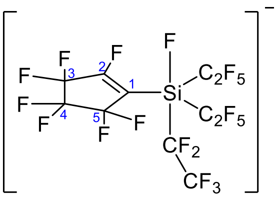

the cation is not depicted and only one possible isomer is shown

^{1}H NMR (300.1 MHz, CD_2Cl_2, rt): δ = 3.14 (dq, $^{3}J_{P,H}$ = 10 Hz, $^{3}J_{H,H}$ = 7 Hz, 36 H, [(CH_3**CH_2**$)_2N]_3P$=N-), 2.04 (d, $^{2}J_{P,H}$ = 7 Hz, 1 H, -P=N(**H**)^{t}Bu), 1.29 (s, 9 H, C(C**H_3**$)_3$), 1.09 ppm (t, $^{3}J_{H,H}$ = 7 Hz, 54 H, [(C**H_3**$CH_2)_2N]_3P$=N-);

^{13}C{^{1}H} NMR (125.8 MHz, CD_2Cl_2, rt): δ = 51.1 (d, $^{2}J_{C,P}$ = 4 Hz, P=N(H)**C**$(CH_3)_3$), 39.5 (d, $^{2}J_{C,P}$ = 6 Hz, [(CH_3**C**$H_2)_2N]_3P$=N-), 31.7 (d, $^{3}J_{C,P}$ = 5 Hz, -P=N(H)C(**C**$H_3)_3$), 13.7 ppm (d, $^{3}J_{C,P}$ = 4 Hz, [(**C**$H_3CH_2)_2N]_3P$=N-);

^{13}C{^{19}F} NMR (75.5 MHz, CD_2Cl_2, rt): δ = 157.5 (s, C_2), 125.7 (s, C_1), 121.5 (s, -CF_2**C**F_3), 121.2 (s, -**C**F_2CF_3), 118.3 (s, C_5), 112.0 (s, C_3), 111.7 ppm (s, C_4);

^{19}F NMR (470.7 MHz, CD_2Cl_2 rt): δ = −64.9 (m,1 F, Si**F**), − 81.5 (d, $^{4}J_{F,F}$ = 11 Hz, 9 F, -CF_2C**F_3**), −102.4 (s, br, 2 F, F_5), −117.1 (s, br, 1 F, F_2), −120.3 (dm, $^{3}J_{F,F}$ = 17 Hz, 2 F, F_3), −121.6 (s, br, 6 F, C**F_2**CF_3), −131.4 ppm (m, F_4);

^{29}Si{^{19}F}-DEPT30 (99.4 MHz, CD_2Cl_2, rt): δ = −105.6 ppm (d, $^{1}J_{Si,F}$ = 203 Hz, Si);

^{31}P NMR (202.5 MHz, CD_2Cl_2, rt): δ = 7.6 (dm, $^{2}J_{P,P}$ = 70, $^{3}J_{P,H}$ = 10 Hz, 3 P, [$(Et_2N)_3$**P**=N$]_3$PN(H)^{t}Bu), −33.8 ppm (qd, $^{2}J_{P,P}$ = 70, $^{2}J_{P,H}$ = 7 Hz, 1 P, [$(Et_2N)_3$P=N$]_3$**P**N(H)^{t}Bu));

IR (ATR): $\tilde{\nu}$ = 2975 (w), 2938 (w), 2874 (w), 1664 (w), 1464 (w, br), 1378 (m), 1352 (m), 1275 (s, br), 1202 (s), 1174 (vs), 1132 (s), 1018 (vs), 963 (s), 941 (vs), 849 (m), 792 (s), 741 (m), 701 (vs), 612 (m), 591 (m), 508 (s), 497 (s), 479 (s), 437 (s), 424 (s), 406 (s) cm^{-1};

MS (ESI, pos., ACN): *m/z* (%): 887 (100) [EtP_4H]$^{+}$; MS (ESI, neg., ACN): *m/z* (%): 597 (69) [$Si(C_2F_5)_3(C_5F_7)F$]$^{-}$, 497 (8) [$Si(C_2F_5)_2(C_5F_7)F_2$]$^{-}$, 423 (100) [$Si(C_2F_5)_3F_2$]$^{-}$, 301 (3) [$Si(C_2F_5)_2FO$]$^{-}$;

Elemental analysis calcd. (%) for $C_{51}H_{100}F_{23}N_{13}P_4Si$: C 41.27, H 6.79, N 12.27, Si 1.89, F 29.44; found: C 40.84, H 6.59, N 12.14, Si 2.01, F 29.12.

[EtP_4H][$Si(C_2F_5)_3(C_3F_5)F$] (1f): [EtP_4H][$Si(C_2F_5)_3$] (0.44 mg, 0.35 mmol) and hexafluoropropene (86 mg, 0.57 mmol) afforded [EtP_4H][$Si(C_2F_5)_3(C_3F_5)F$] (0.490 mg, 0.35 mmol) in a quantitative yield.

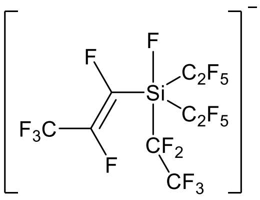

the cation is not depicted and only one possible isomer is shown

^{1}H NMR (300.1 MHz, CD_2Cl_2, rt): δ = 3.14 (dq, $^3J_{P,H}$ = 10 Hz, $^3J_{H,H}$ = 7 Hz, 36 H, [($CH_3C\mathbf{H}_2)_2N]_3P$=N-), 2.03 (d, $^2J_{P,H}$ = 7 Hz, 1 H, -P=N(**H**)^{t}Bu), 1.28 (s, 9 H, C($C\mathbf{H}_3)_3$), 1.09 ppm (t, $^3J_{H,H}$ = 7 Hz, 54 H, [($C\mathbf{H}_3CH_2)_2N]_3P$=N-);

^{13}C{^{1}H} NMR (125.8 MHz, CD_2Cl_2, rt): δ = 51.0 (d, $^2J_{C,P}$ = 4 Hz, P=N(H)**C**($CH_3)_3$), 39.5 (d, $^2J_{C,P}$ = 6 Hz, [($CH_3\mathbf{C}H_2)_2N]_3P$=N-), 31.7 (d, $^3J_{C,P}$ = 5 Hz, -P=N(H)C($\mathbf{C}H_3)_3$), 13.7 ppm (d, $^3J_{C,P}$ = 4 Hz, [($\mathbf{C}H_3CH_2)_2N]_3P$=N-);

^{13}C{^{19}F} NMR (75.5 MHz, CD_2Cl_2, rt): δ = 168.1 ppm (dq, CF=**C**F(CF_3), 147.0 (s, Si**C**F=CF(CF_3)), 121.3 (s, -$CF_2\mathbf{C}F_3$), 120.2 (s, -CF=CF(**C**F_3) and -**C**F_2CF_3 overlapped);

^{19}F NMR (470.7 MHz, CD_2Cl_2 rt): δ = −67.5 (dd, $^3J_{F,F}$ = 23 Hz, $^4J_{F,F}$ = 10 Hz, 3 F, CF=CF(C**F**$_3$)), −76.7 (m, 1 F, Si**F**), −82.0 (dm, $^4J_{F,F}$ = 14 Hz, 9 F, $CF_2C\mathbf{F}_3$), −123.1 (dd, $^3J_{F,F}$ = 21 Hz, $^4J_{F,F}$ = 11 Hz, 6 F, C**F**$_2CF_3$), −152.5 (dm, $^3J_{F,F}$ = 130 Hz, 1 F, CF=C**F**(CF_3)), −168.7 ppm (dm, $^3J_{F,F}$ = 130 Hz, 1 F, C**F**=CF(CF_3));

^{29}Si{^{19}F}-DEPT30 (99.4 MHz, CD_2Cl_2, rt): δ = −107.3 ppm (dm, $^1J_{Si,F}$ = 309 Hz, Si);

^{31}P NMR (202.5 MHz, CD_2Cl_2, rt): δ = 7.6 (dm, $^2J_{P,P}$ = 70, $^3J_{P,H}$ = 10 Hz, 3 P, [($Et_2N)_3$**P**=N]$_3$PN(H)^{t}Bu), −33.8 ppm (qd, $^2J_{P,P}$ = 70, $^2J_{P,H}$ = 7 Hz, 1 P, [($Et_2N)_3P$=N]$_3$**P**N(H)^{t}Bu));

IR (ATR): $\tilde{\nu}$ = 2970 (w), 2934 (w), 2873 (w), 1465 (w), 1379 (m), 1350 (m), 1272 (s, br), 1201 (s), 1172 (vs), 1129 (s), 1055 (m), 1017 (vs), 941 (vs), 847 (m), 794 (s), 735 (m), 700 (s), 663 (m), 614 (m), 592 (m), 508 (s), 484 (s), 441 (s), 421 (s) cm^{-1};

MS (ESI, pos., ACN): *m/z* (%): 887 (100) [EtP_4H]$^+$; MS (ESI, neg., ACN): *m/z* (%): 535 (19) [$Si(C_2F_5)_3(C_3F_5)F$]$^-$, 435 (100) [$Si(C_2F_5)_2(C_3F_5)F_2$]$^-$, 423 (10) [$Si(C_2F_5)_3F_2$]$^-$, 323 (11) [$Si(C_2F_5)_2F_3$]$^-$, 123 (10) [SiF_5]$^-$;

Elemental analysis calcd. (%) for $C_{49}H_{100}F_{21}N_{13}P_4Si$: C 41.38, H 7.09, N 12.80, Si 1.97, F 28.05; found: C 41.23, H 7.00, N 12.72, Si 1.95, F 27.99.

2 NMR Spectra

Since the ^{1}H, ^{13}C{^{1}H} and ^{31}P NMR spectra of silicates **1a-f** look virtually the same, these are only depicted for $[EtP_4H][Si(C_2F_5)_3(C_7F_7)F]$ (**1a**).

2.1 NMR spectra of $[EtP_4H][Si(C_2F_5)_3(C_7F_7)F]$ (1a)

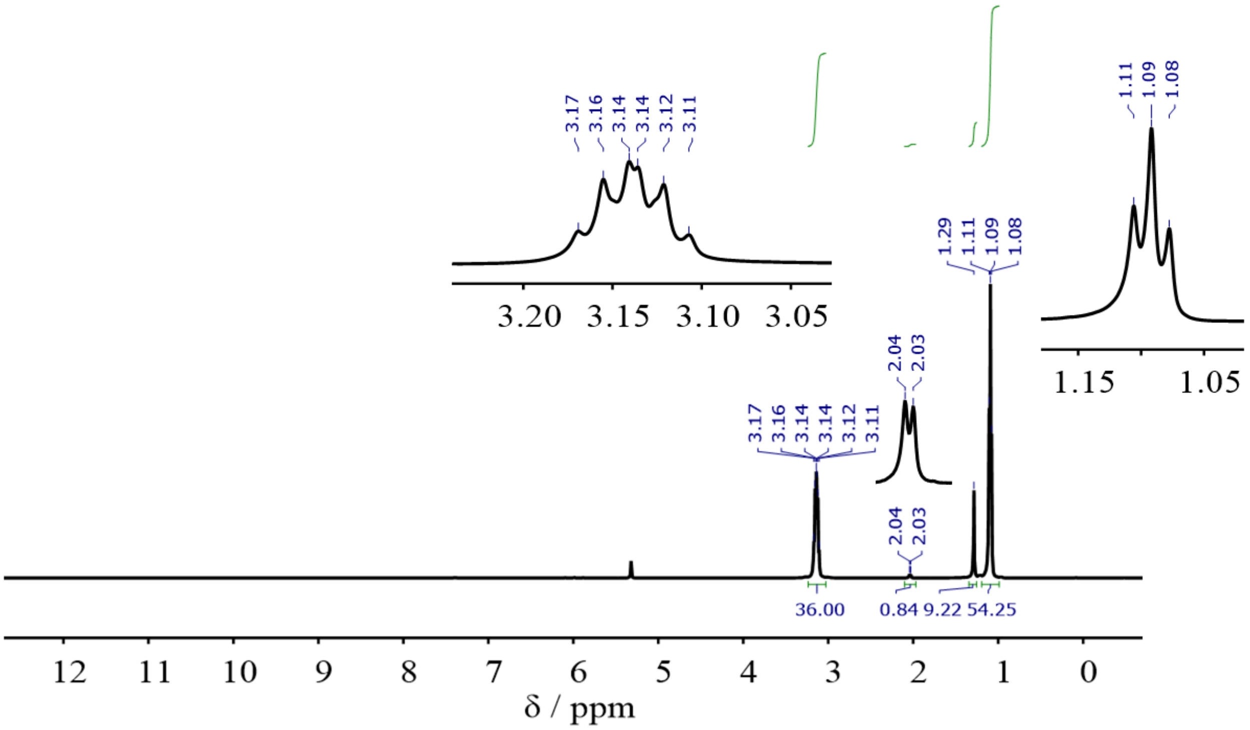

Figure S1. ^{1}H NMR spectrum of $[EtP_4H][Si(C_2F_5)_3(C_7F_7)F]$ (**1a**).

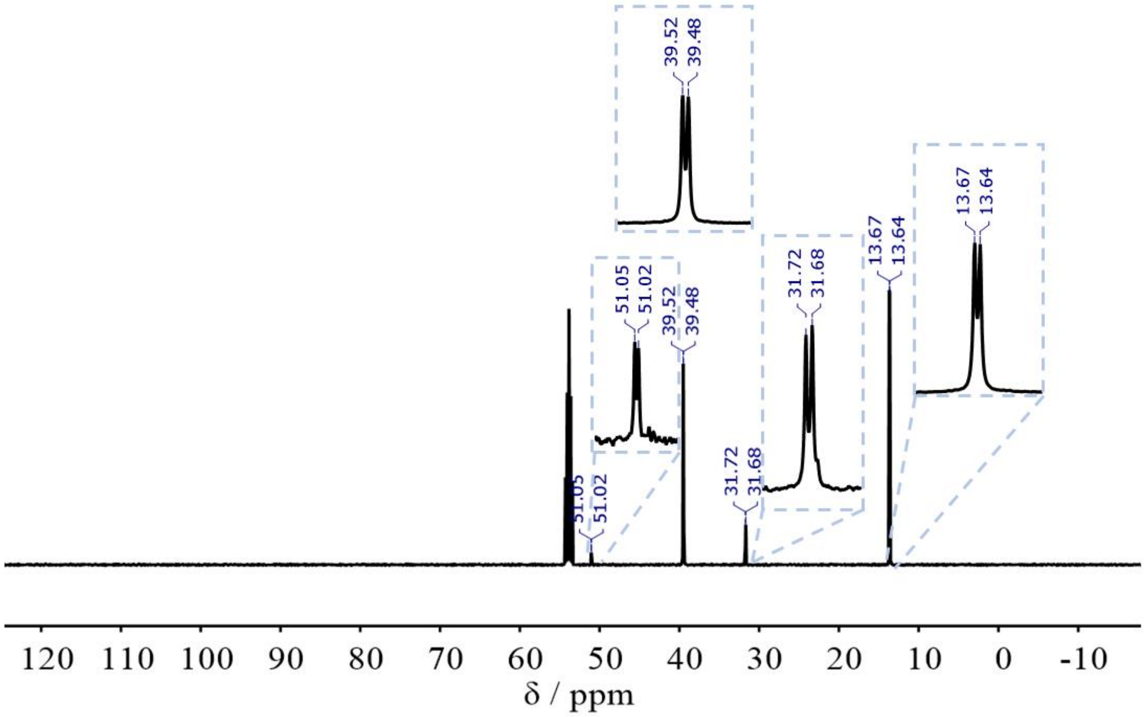

Figure S2. ^{13}C{^{1}H} NMR spectrum of $[EtP_4H][Si(C_2F_5)_3(C_7F_7)F]$ (**1a**).

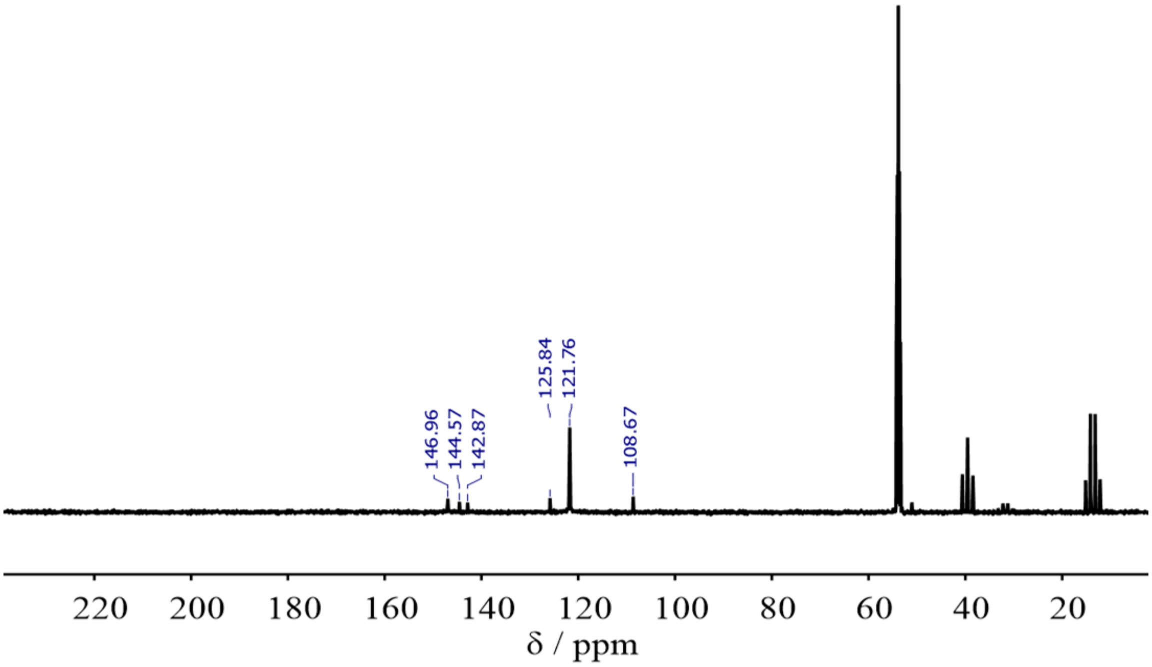

Figure S3. $^{13}C\{^{19}F\}$ NMR spectrum of $[EtP_4H][Si(C_2F_5)_3(C_7F_7)F]$ (**1a**).

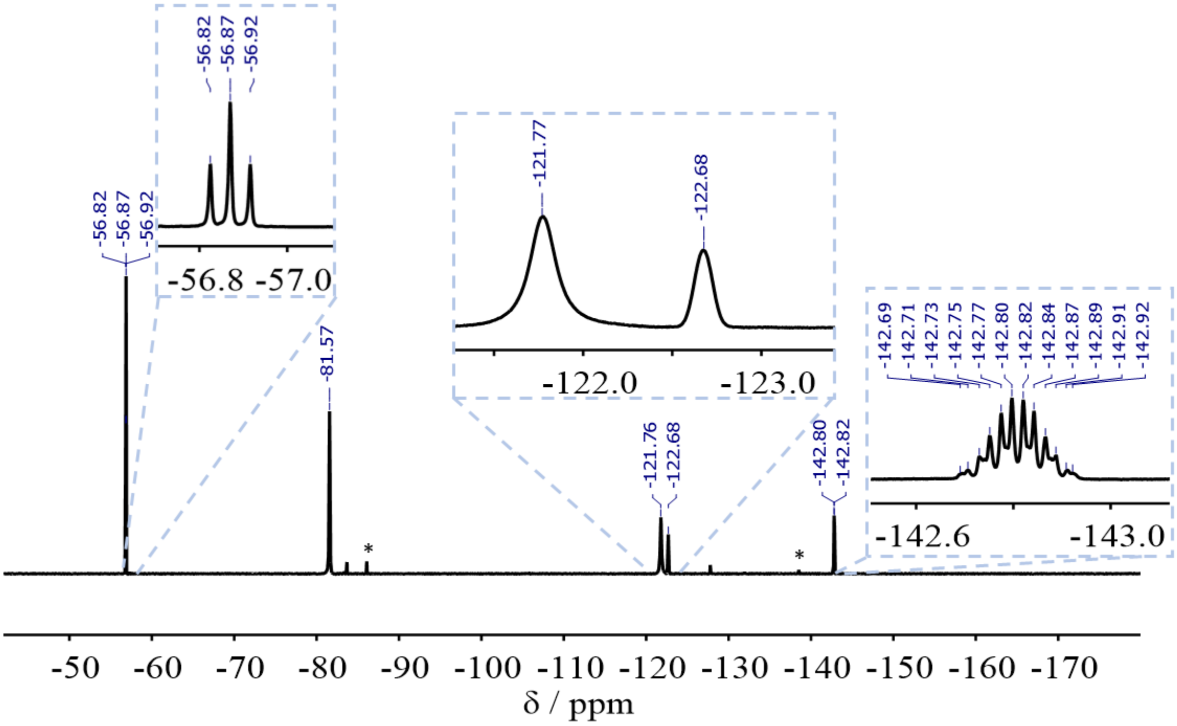

Figure S4. ^{19}F NMR spectrum of $[EtP_4H][Si(C_2F_5)_3(C_7F_7)F]$ (**1a**). *signals for HC_2F_5

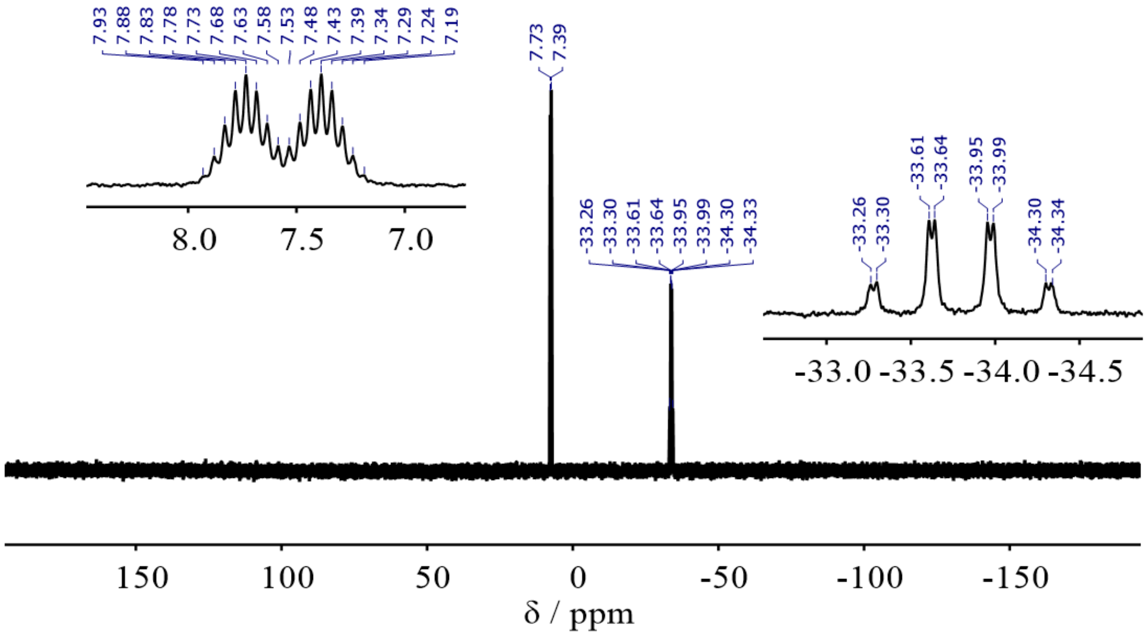

Figure S5. ^{31}P NMR spectrum of $[EtP_4H][Si(C_2F_5)_3(C_7F_7)F]$ (**1a**).

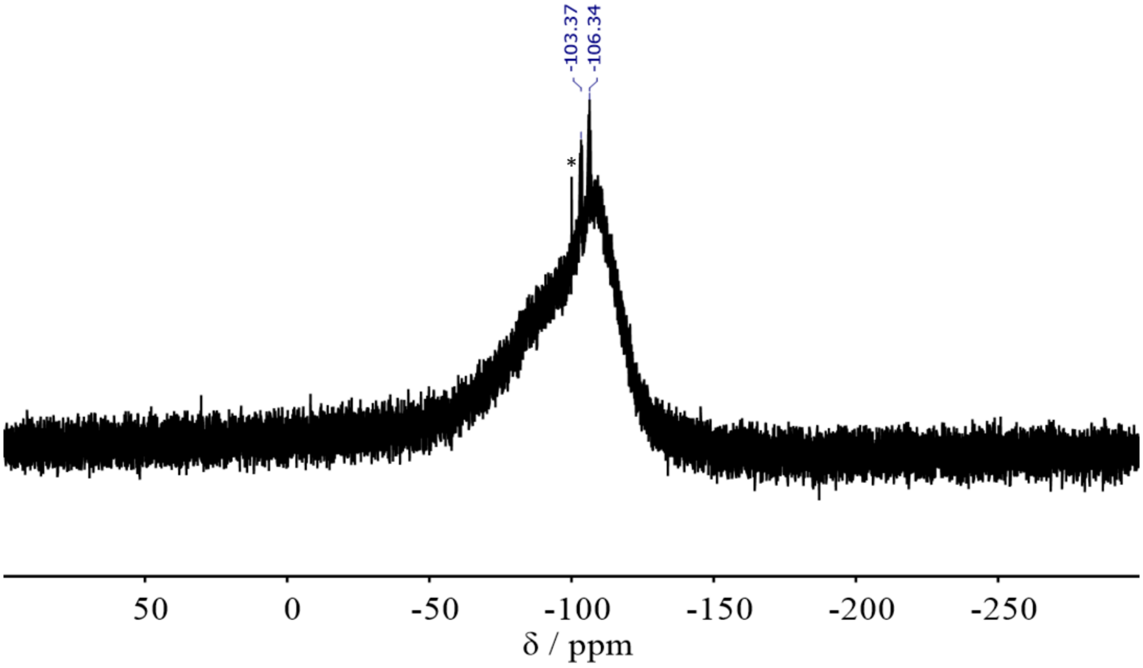

Figure S6. ^{29}Si{^{1}H} IG NMR spectrum of $[EtP_4H][Si(C_2F_5)_3(C_7F_7)F]$ (**1a**). * unidentified impurities

2.2 NMR spectra of [EtP$_4$H][Si(C_2F_5)$_3$(C_6F_5)F] (1b)

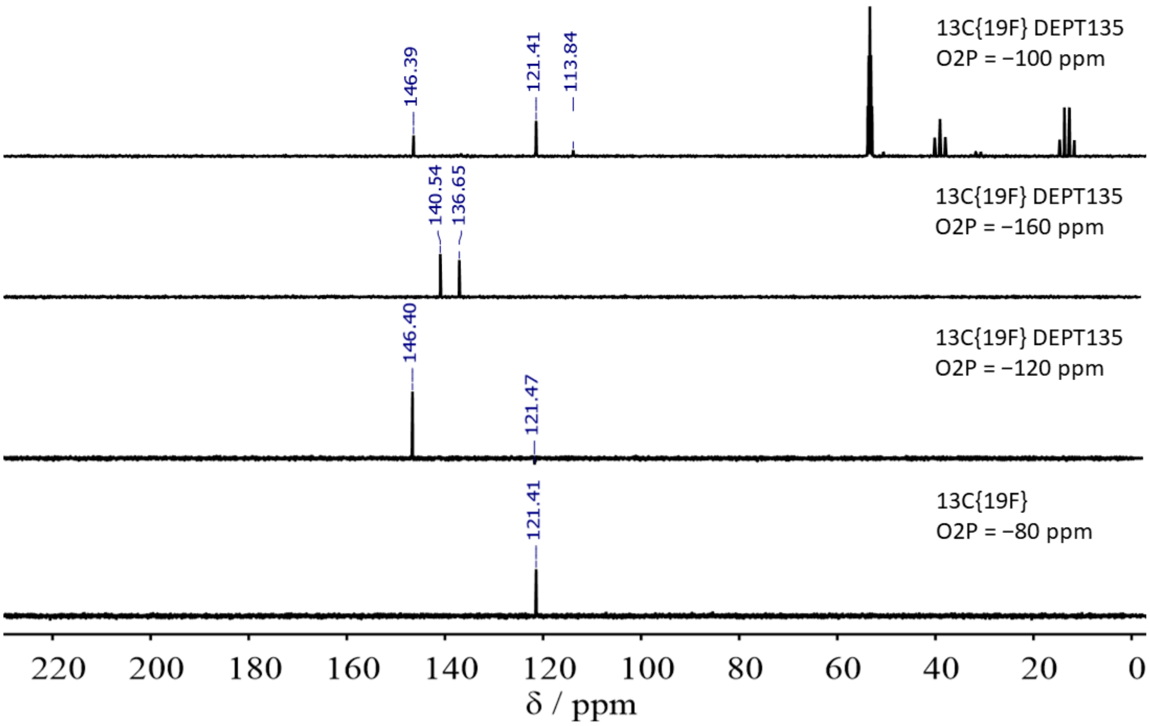

Figure S7. ^{13}C{^{19}F} NMR spectra of [EtP$_4$H][Si(C_2F_5)$_3$(C_6F_5)F] (**1b**) with different chemical shifts of the decoupled nucleus (O2P values).

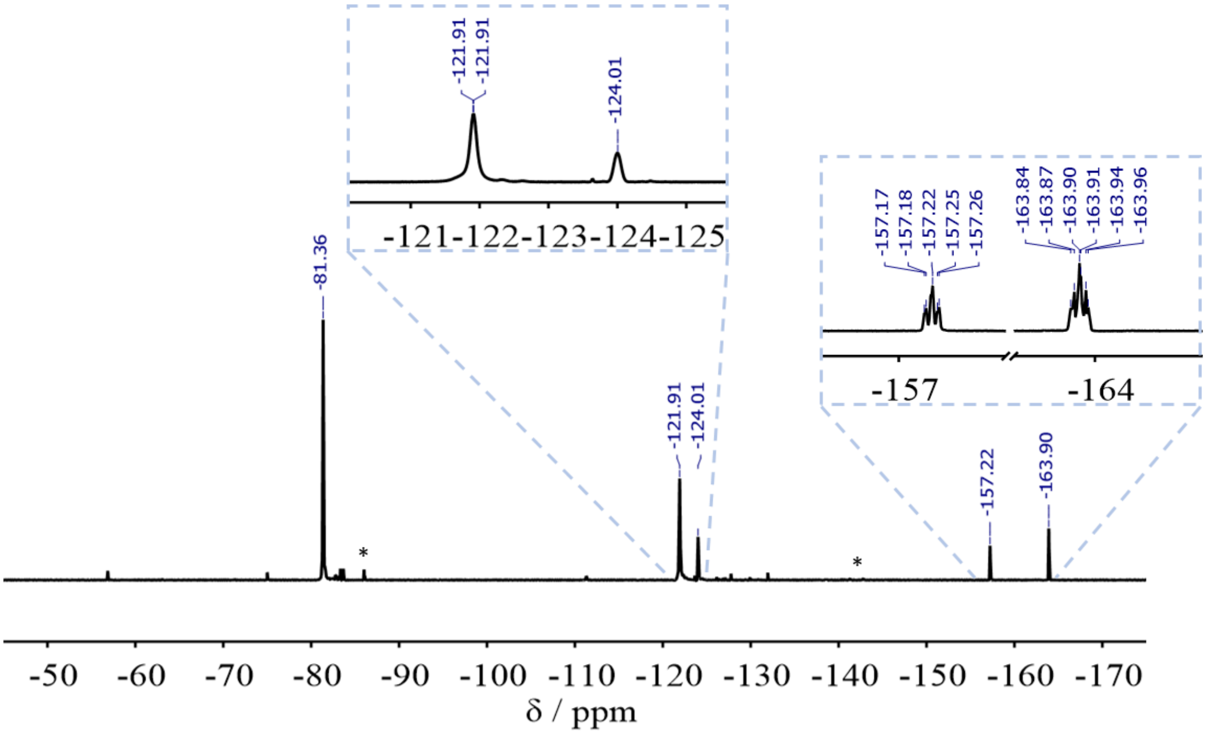

Figure S8. ^{19}F NMR spectrum of [EtP$_4$H][Si(C_2F_5)$_3$(C_6F_5)F] (**1a**). *signals for HC_2F_5

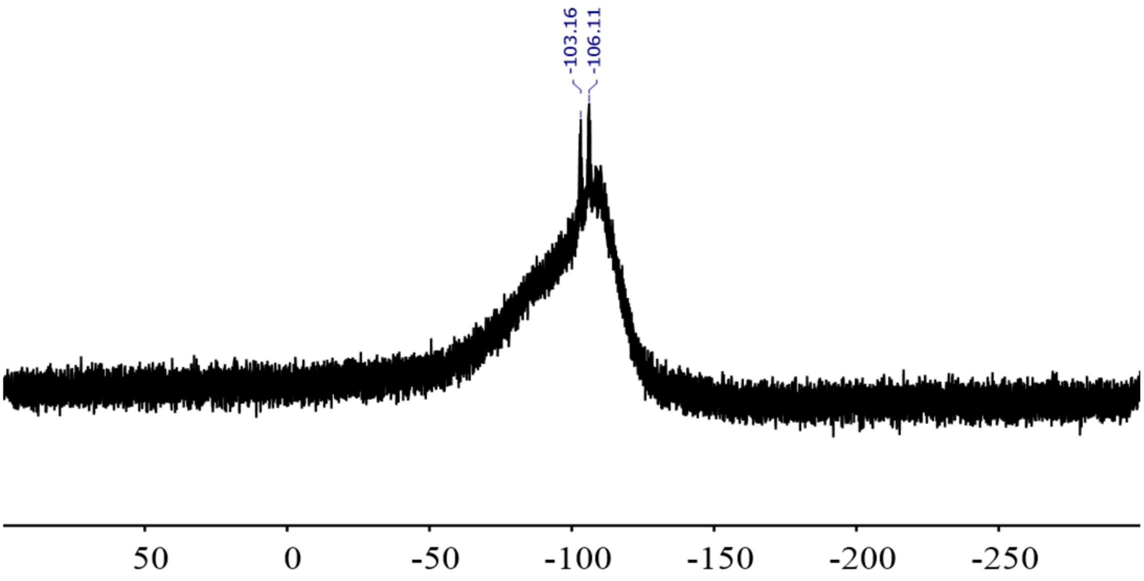

Figure S9. $^{29}Si\{^{1}H\}$ IG NMR spectrum of $[EtP_4H][Si(C_2F_5)_3(C_6F_5)F]$ (**1b**).

2.3 NMR spectra of $[EtP_4H][Si(C_2F_5)_3(C_{10}F_7)F]$ (1c)

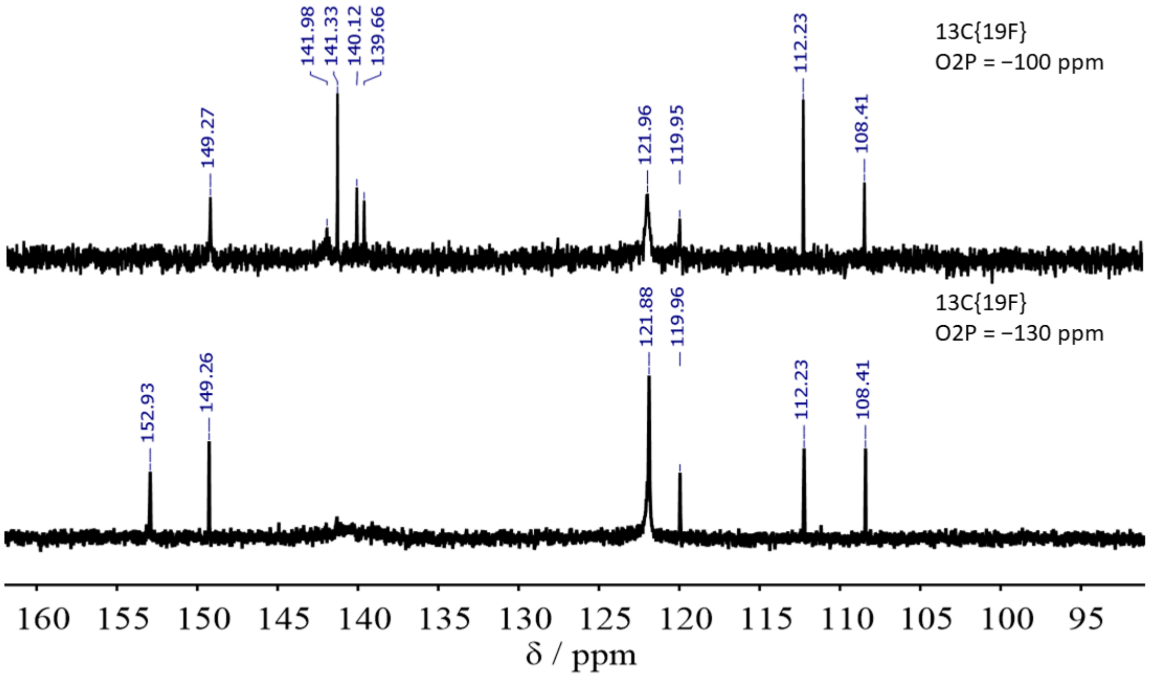

Figure S10. $^{13}C\{^{19}F\}$ NMR spectra of $[EtP_4H][Si(C_2F_5)_3(C_{10}F_7)F]$ (**1c**) with different chemical shifts of the decoupled nucleus (O2P values).

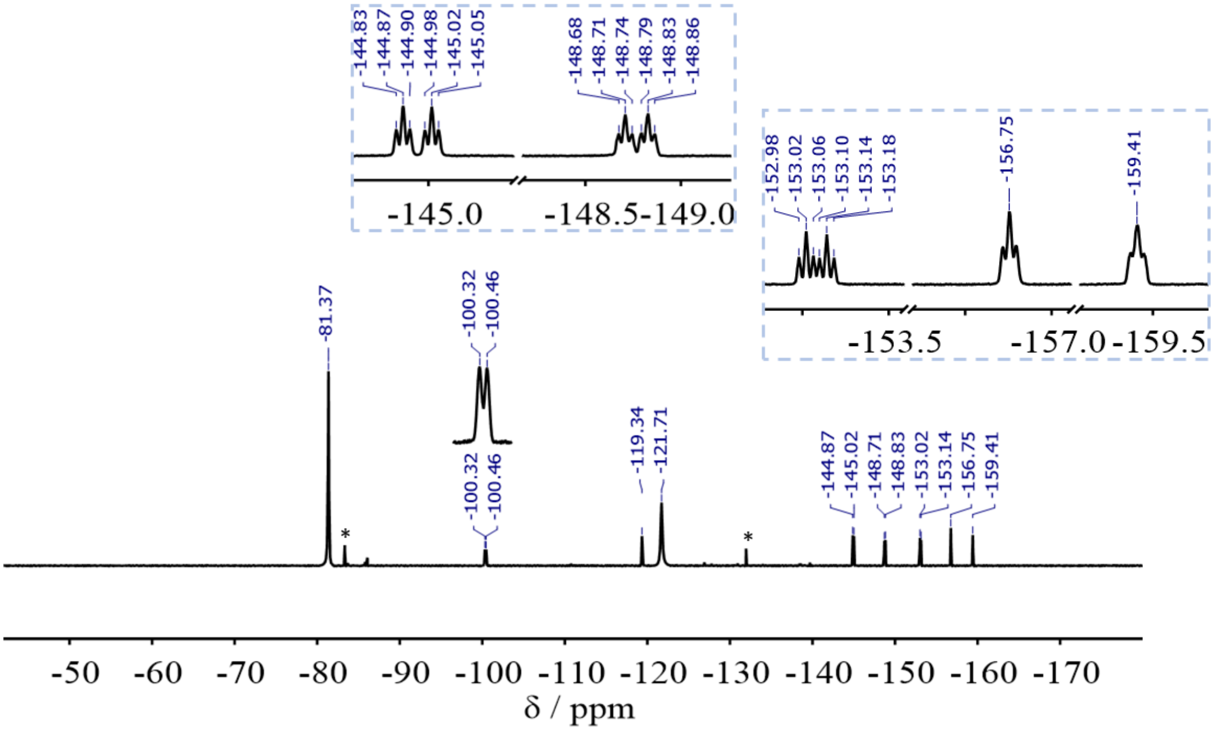

Figure S11. ^{19}F NMR spectrum of $[EtP_4H][Si(C_2F_5)_3(C_{10}F_7)F]$ (**1c**). *signals for HC_2F_5

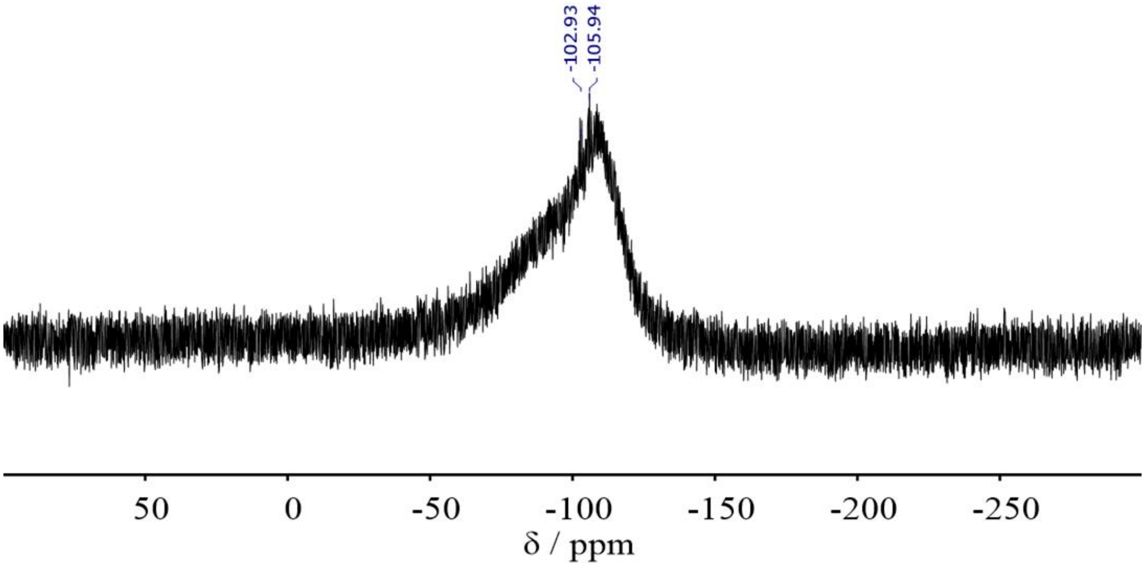

Figure S12. $^{29}Si\{^1H\}$ IG NMR spectrum of $[EtP_4H][Si(C_2F_5)_3(C_{10}F_7)F]$ (**1c**).

2.4 NMR spectra of [EtP$_4$H][Si(C$_2$F$_5$)$_3$(C$_5$F$_4$N)F] (1d)

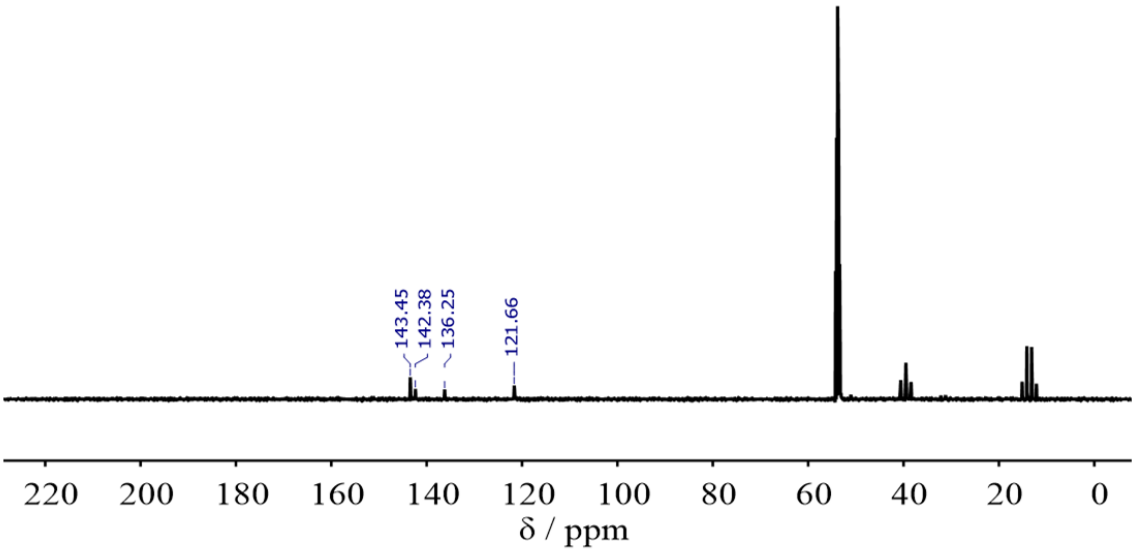

Figure S13. ^{13}C{^{19}F} NMR spectrum of [EtP$_4$H][Si(C$_2$F$_5$)$_3$(C$_5$F$_4$N)F] (**1d**).

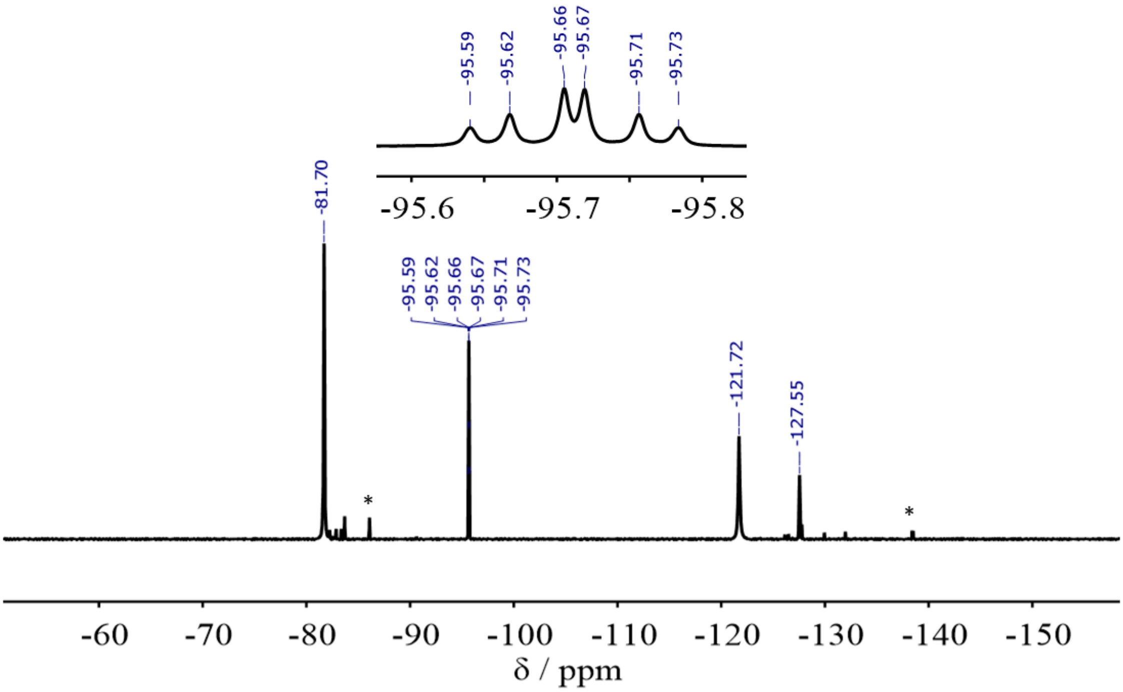

Figure S14. ^{19}F NMR spectrum of [EtP$_4$H][Si(C$_2$F$_5$)$_3$(C$_5$F$_4$N)F] (**1d**). *signals for HC$_2$F$_5$.

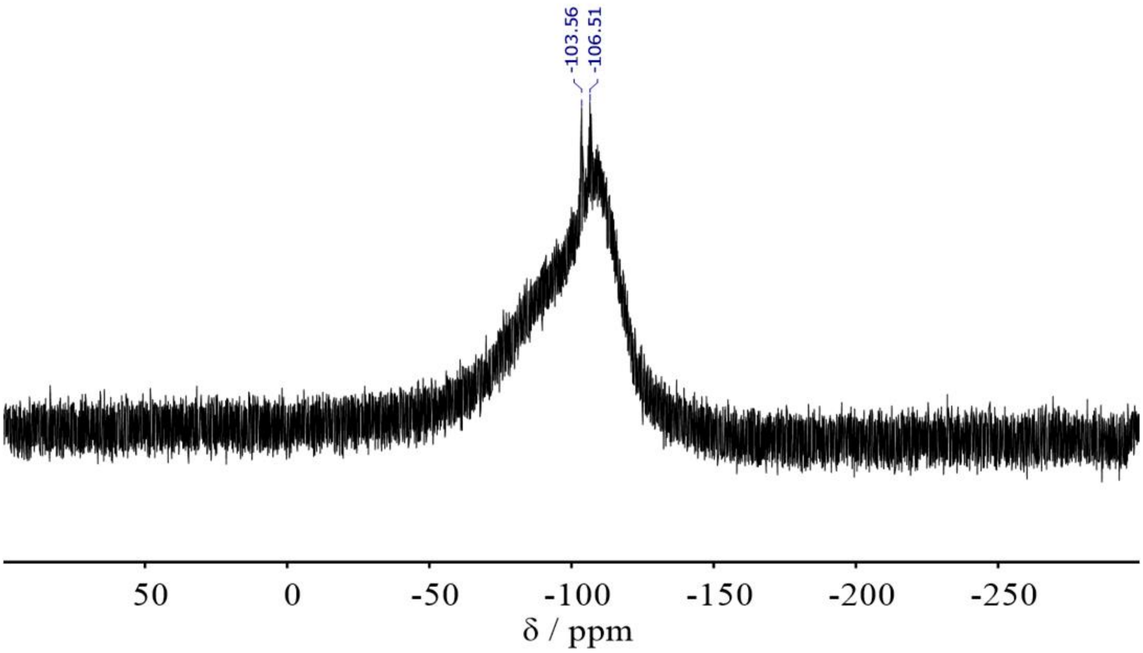

Figure S15. $^{29}Si\{^1H\}$ IG NMR spectrum of $[EtP_4H][Si(C_2F_5)_3(C_5F_4N)F]$ (**1c**).

2.5 NMR spectra of $[EtP_4H][Si(C_2F_5)_3(C_5F_7)F]$ (1e)

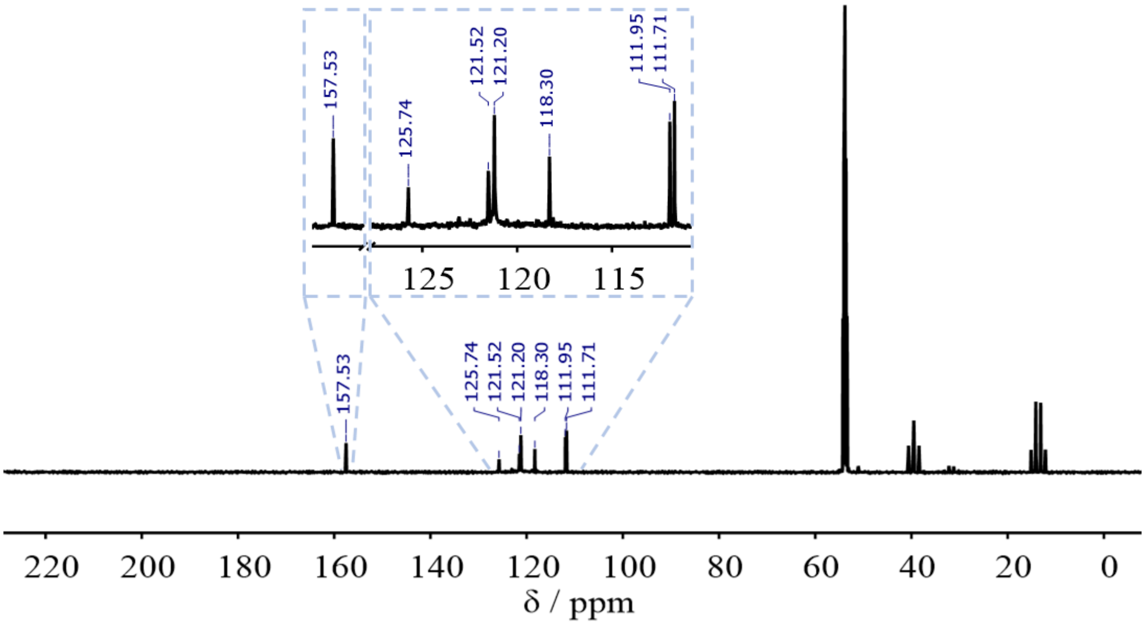

Figure S16. $^{13}C\{^{19}F\}$ NMR spectrum of $[EtP_4H][Si(C_2F_5)_3(C_5F_7)F]$ (**1e**).

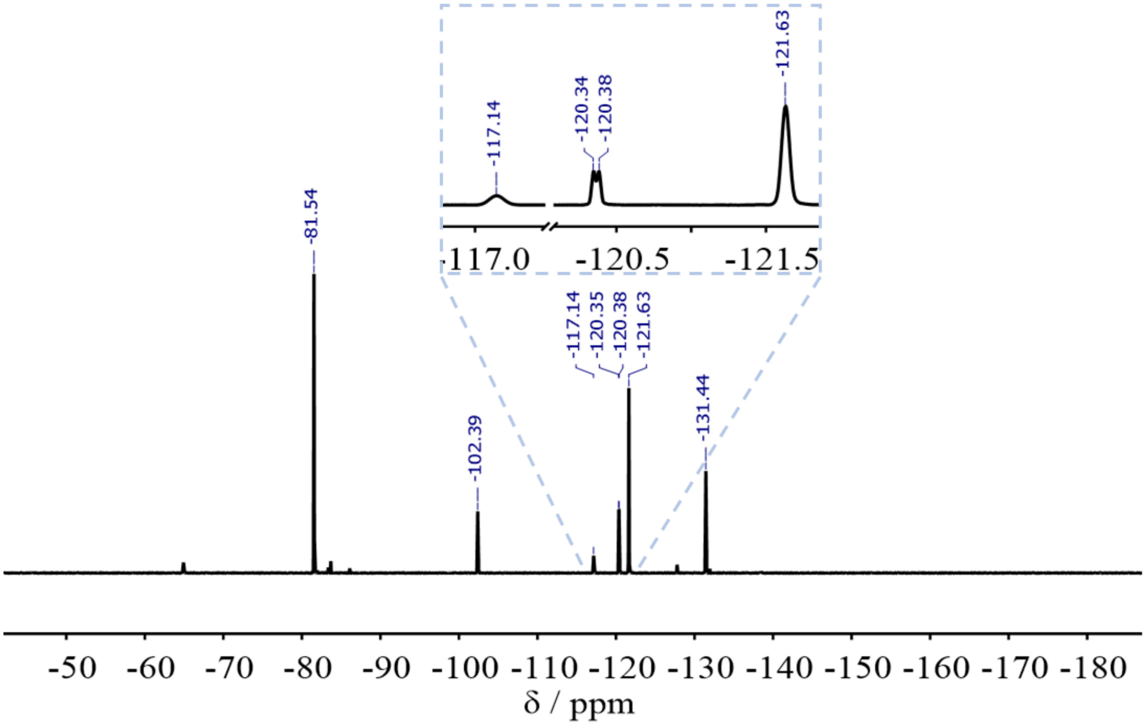

Figure S17. ^{19}F NMR spectrum of [EtP$_4$H][Si(C_2F_5)$_3$(C_5F_7)F] (**1e**).

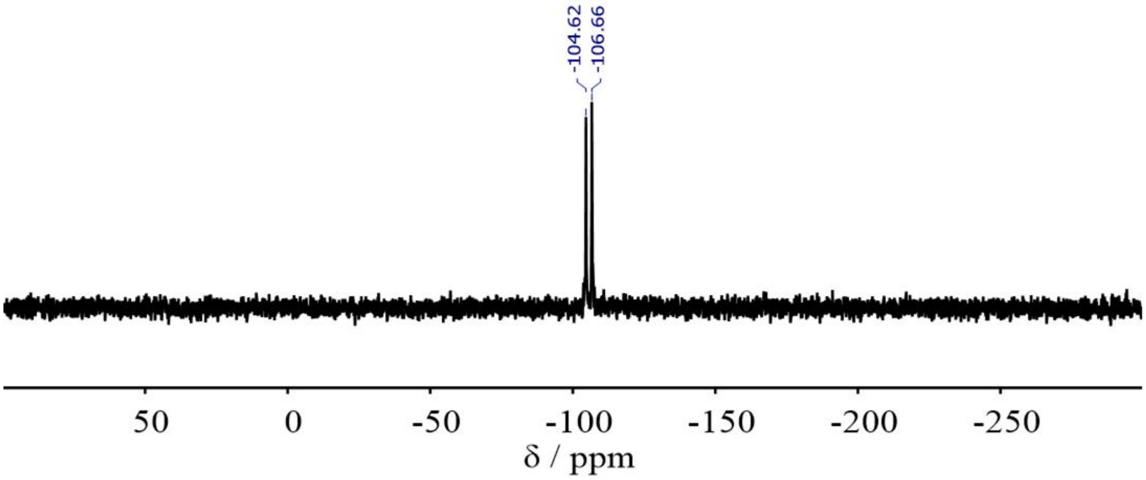

Figure S18. ^{29}Si{^{19}F} NMR spectrum of [EtP$_4$H][Si(C_2F_5)$_3$(C_5F_7)F] (**1e**).

2.6 NMR spectra of $[EtP_4H][Si(C_2F_5)_3(C_3F_5)F]$

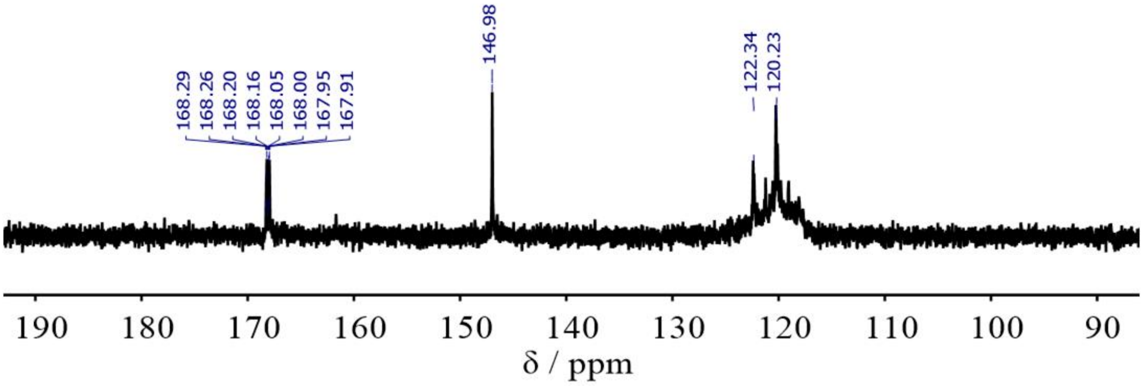

Figure S19. $^{13}C\{^{19}F\}$ NMR spectrum of $[EtP_4H][Si(C_2F_5)_3(C_3F_5)F]$ (**1f**).

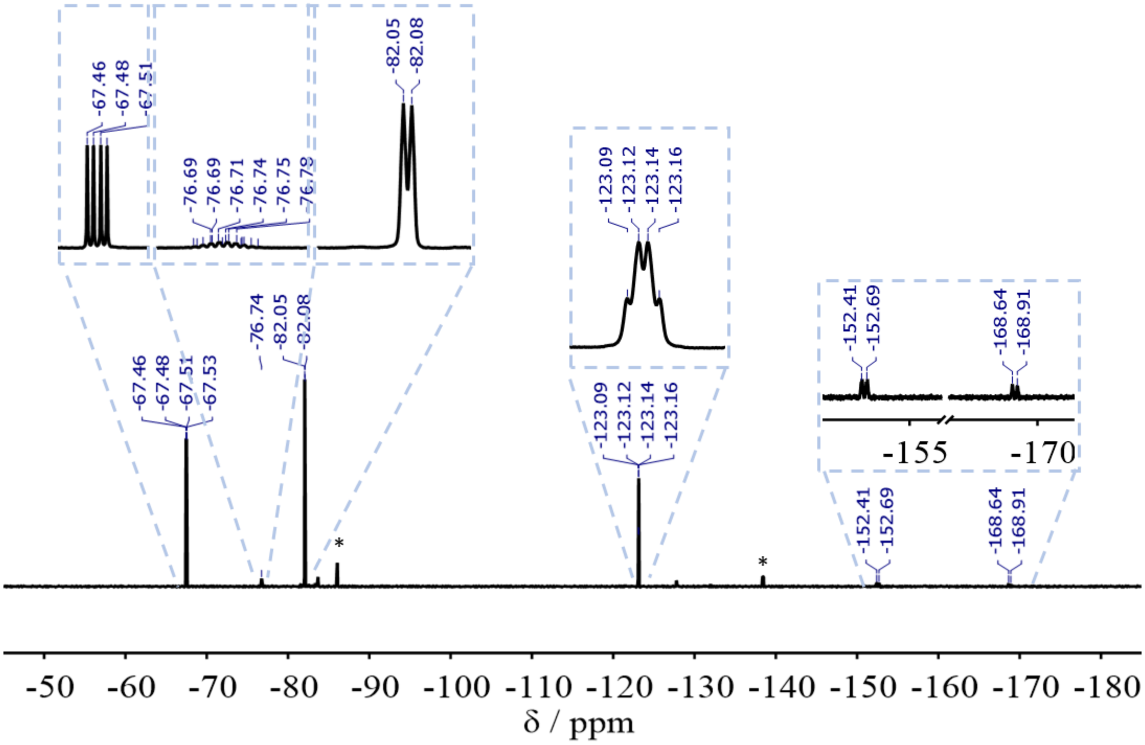

Figure S20. ^{19}F NMR spectrum of $[EtP_4H][Si(C_2F_5)_3(C_3F_5)F]$ (**1f**).

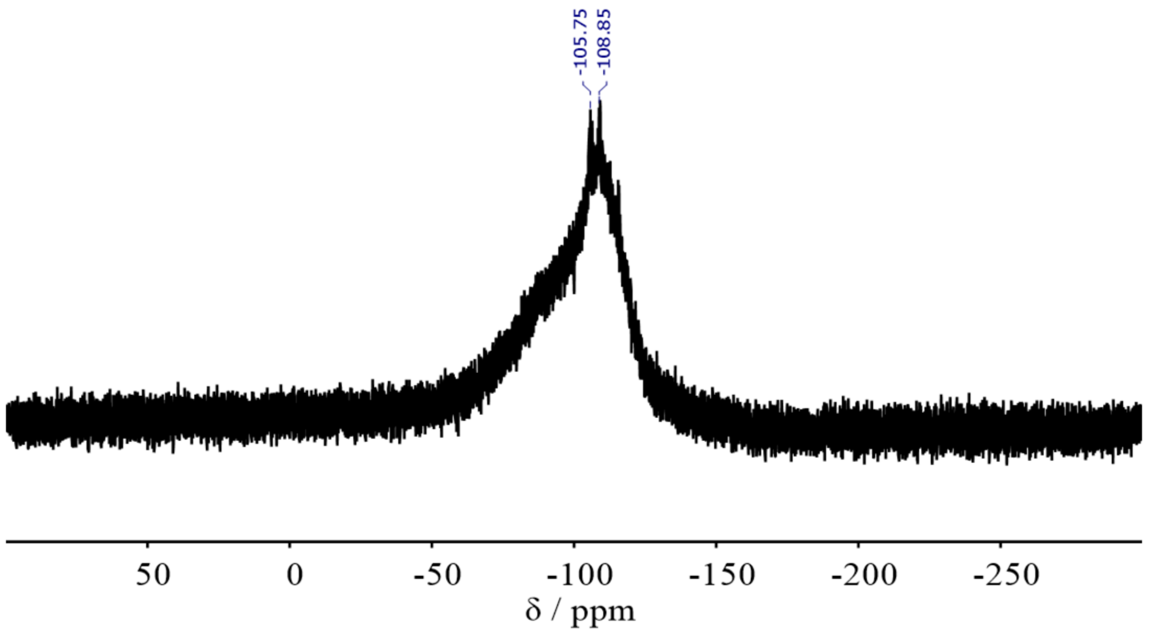

Figure S21. $^{29}Si\{^{19}F\}$ NMR spectrum of $[EtP_4H][Si(C_2F_5)_3(C_3F_5)F]$ (**1f**).

3 X-ray Data

X-ray structure determination

The crystal data were collected on a Rigaku Supernova diffractometer using Cu Kα (λ = 1.54184 Å) radiation. The crystals were kept at 100.0(1) K during data collection. Using Olex2[2], the structures were solved with the SHELXT[3] structure solution program using Intrinsic Phasing and refined with the SHELXL[4] refinement package using Least Squares minimization. In **1a** the CF_3 group of the perfluorotoluene substituent is disordered at one anion with a ratio of 87:13, at the other anion the CF_3 moiety of one C_2F_5 group is disordered with a ratio of 73:27. In **1c** two ethyl groups of the cation are disordered and the C_2F_5 group in a ratio of 89:11. In **1d** three ethyl groups are disordered with ratios of 90:10, 58:42, and 53:47. In **1e** the crystal appears as a racemic twin. Four ethyl groups of the cation and all groups bonded to the silicon of the anion are disordered over two sites with a ratio of 85:15. Suitable restraints and constraints were applied to disordered carbon and fluorine atoms.

Table S1. Structure refinement data **1a-e**.

	$[EtP_4H][Si(C_2F_5)_3(C_7F_7)F]$ (1a)	$[EtP_4H][Si(C_2F_5)_3(C_6F_5)F]$ (1b)	$[EtP_4H][Si(C_2F_5)_3(C_{10}F_7)F]$ (1c)	$[EtP_4H][Si(C_2F_5)_3(C_5F_4N)F]$ (1d)	$[EtP_4H][Si(C_2F_5)_3(C_5F_7)F]$ (1e)
**Empirical formula**	$C_{53}H_{100}F_{23}N_{13}P_4Si$	$C_{52}H_{100}F_{21}N_{13}P_4Si$	$C_{56}H_{100}F_{23}N_{13}P_4Si$	$C_{51}H_{100}F_{20}N_{14}P_4Si$	$C_{51}H_{100}F_{23}N_{13}P_4Si$
**Formula weight**	1508.42	1458.41	1544.45	1441.41	1484.40
**Crystal system**	triclinic	orthorhombic	triclinic	triclinic	monoclinic
**Space group**	$P\bar{1}$	$P2_12_12_1$	$P\bar{1}$	$P\bar{1}$	$P2_1$
**a/Å**	13.5000(2)	11.8798(2)	13.2244(3)	11.7988(3)	13.6150(5)
**b/Å**	22.1410(3)	16.3834(3)	15.0647(5)	16.5318(5)	15.8534(7)
**c/Å**	23.8194(4)	35.4911(7)	18.8002(6)	17.9047(5)	16.3232(7)
**α/°**	89.9961(12)	90	79.482(3)	88.296(2)	90
**β/°**	87.2836(12)	90	78.039(2)	83.625(2)	94.701(4)
**γ/°**	89.4024(11)	90	85.459(2)	86.104(2)	90
**Volume/Å^3**	7111.34(18)	6907.7(2)	3599.20(19)	3461.91(16)	3511.4(3)
**Z**	4	4	2	2	2
**ρ_{calc} /g cm^{-3}**	1.409	1.402	1.425	1.383	1.404
**μ/mm^{-1}**	2.082	2.081	2.072	2.052	2.098
**F(000)**	3160.0	3064.0	1616.0	1516.0	1556.0
**Crystal size/mm^3**	0.21 × 0.18 × 0.14	0.16 × 0.10 × 0.08	0.18 × 0.11 × 0.10	0.20 × 0.18 × 0.14	0.17 × 0.10 × 0.04
**2Θ range /°**	5.4 - 148.5	5.9 - 152.1	4.9 - 152.6	5.4 - 153.1	5.4 - 153.2
**Index ranges**	−16 ≤ h ≤ 16, −27 ≤ k ≤ 27, −29 ≤ l ≤ 29	−14 ≤ h ≤ 10, −17 ≤ k ≤ 20, −43 ≤ l ≤ 39	−12 ≤ h ≤ 16, −18 ≤ k ≤ 17, −23 ≤ l ≤ 23	−14 ≤ h ≤ 14, −20 ≤ k ≤ 19, −22 ≤ l ≤ 15	−16 ≤ h ≤ 15, −19 ≤ k ≤ 19, −20 ≤ l ≤ 20
**Reflections collected**	135222	26335	31742	30802	32907
**Independent reflections**	28494	14038	14772	14216	14367

	[EtP$_4$H][Si(C$_2$F$_5$)$_3$(C$_7$F$_7$)F] (1a)	[EtP$_4$H][Si(C$_2$F$_5$)$_3$(C$_6$F$_5$)F] (1b)	[EtP$_4$H][Si(C$_2$F$_5$)$_3$(C$_{10}$F$_7$)F] (1c)	[EtP$_4$H][Si(C$_2$F$_5$)$_3$(C$_5$F$_4$N)F] (1d)	[EtP$_4$H][Si(C$_2$F$_5$)$_3$(C$_5$F$_7$)F] (1e)
**R(int)**	0.0300	0.0392	0.0368	0.0263	0.0435
**R(sigma)**	0.0200	0.0627	0.0460	0.0330	0.0529
**Reflections with I > 2σ(I)**	26204	12094	12374	12558	12206
**Data/restraints/parameters**	28494/150/1817	14038/0/875	14772/44/991	14216/6/872	14367/561/1152
**Goodness-of-fit on F^2**	1.013	1.021	1.027	1.019	1.072
**Final R indexes [I > 2σ(I)]**	R_1 = 0.0332, wR_2 = 0.0867	R_1 = 0.0428, wR_2 = 0.0945	R_1 = 0.0431, wR_2 = 0.1033	R_1 = 0.0324, wR_2 = 0.0797	R_1 = 0.0543, wR_2 = 0.1429
**Final R indexes [all data]**	R_1 = 0.0361, wR_2 = 0.0892	R_1 = 0.0534, wR_2 = 0.0993	R_1 = 0.0529, wR_2 = 0.1096	R_1 = 0.0381, wR_2 = 0.0834	R_1 = 0.0649, wR_2 = 0.1585
**Max. diff. peak/hole / e Å^{-3}**	0.63 / −0.46	0.64 / −0.38	0.77 / −0.47	0.34 / −0.43	0.46 / −0.37
**Flack parameter**		−0.015(10)			0.38(3)
**CCDC number**	2112946	2112947	2112948	2112949	2112950

4 DFT calculations[5]

Geometry optimizations were performed on the B3LYP/6-31+G(3d,p) level of theory. Thermochemical values used for the calculation of proton affinities were calculated on the B3LYP/6-311+G(3d,2p) level of theory. For the reaction pathways of the oxidative additions solvation effects were considered by means of the default (SCRD) model of solvation with the solvent diethyl ether. Transition states were located using the QST2 method. All calculations were carried out with an ultrafine integration grid (99,590). Frequency calculations were conducted to characterize all stationary points.

4.1 Proton affinities

Table S2. Energy release by addition of a proton to the silanides $[Si(C_2F_5)_3]^-$, $[Si(CF_3)_3]^-$, $[Si(C_2H_5)_3]^-$, and $[Si(CH_3)_3]^-$.

	−ΔE / kcal·mol⁻¹	−ΔH / kcal·mol⁻¹	−ΔG / kcal·mol⁻¹
$[Si(C_2F_5)_3]^- + H^+ \rightarrow Si(C_2F_5)_3H$	320.2	320.1	320.6
$[Si(CF_3)_3]^- + H^+ \rightarrow Si(CF_3)_3H$	322.7	322.6	323.4
$[Si(C_2H_5)_3]^- + H^+ \rightarrow Si(C_2H_5)_3H$	382.2	382.2	382.3
$[Si(CH_3)_3]^- + H^+ \rightarrow Si(CH_3)_3H$	385.6	385.5	386.3

Table S3. Cartesian coordinates and calculated thermochemical values of $[SiR_3]^-$ and SiR_3H for R = C_2F_5, C_2H_5, CF_3, and CH_3.

Molecule	Coordinates				Thermochemical values / Hartree	
$[Si(C_2F_5)_3]^-$	Si	0.00630500	0.06286500	-1.29791700	Sum of electronic and zero-point Energies=	-2016.387299
	C	1.37228100	-1.15572300	-0.44210700	Sum of electronic and thermal Energies=	-2016.365430
	C	-1.62975100	-0.81440800	-0.49989500	Sum of electronic and thermal Enthalpies=	-2016.364486
	C	0.15126300	1.56521800	0.04569600	Sum of electronic and thermal Free Energies=	-2016.439659
	F	2.60186700	-0.64322400	-0.85856300		
	F	1.44398800	-1.19558300	0.93714200		
	F	-1.84385200	-1.93944200	-1.29763800		
	F	-1.55188100	-1.29558700	0.79291800		
	F	-0.79138800	2.50244900	-0.37966700		
	F	-0.16928600	1.30055800	1.36335700		
	C	1.47108800	2.37312900	0.11987000		
	C	1.42217200	-2.64143300	-0.87825800		
	C	-2.98070300	-0.05816900	-0.55435100		
	F	0.41361300	-3.34831700	-0.34717200		
	F	1.35393800	-2.75838900	-2.21870600		
	F	2.57300800	-3.23705400	-0.48627600		
	F	1.91040000	2.70272300	-1.11040000		
	F	2.44138100	1.68253800	0.73679000		
	F	1.30950900	3.52891700	0.80607400		
	F	-3.02963300	0.93531700	0.34543300		
	F	-3.19306100	0.47244600	-1.77440500		
	F	-4.02317300	-0.88139800	-0.29298400		
$[Si(C_2H_5)_3]^-$	Si	-0.00327900	0.01676000	-1.06598800	Sum of electronic and zero-point Energies=	-527.094085
	C	1.28128000	-1.24396800	-0.28091900	Sum of electronic and thermal Energies=	-527.083327
	C	-1.64326300	-0.75106500	-0.30685700	Sum of electronic and thermal Enthalpies=	-527.082383
	C	1.43281100	2.41020600	-0.04861400	Sum of electronic and thermal Free Energies=	-527.129503
	C	1.45483400	-2.57174500	-1.03557400		
	C	-2.95364800	-0.06581800	-0.72620700		
	H	1.36744100	2.85921300	-1.04838800		
	C	0.23336200	1.48595100	0.21516800		
	H	1.51599100	3.23125300	0.68159300		
	H	2.38000800	1.85488500	-0.01618000		
	H	-0.68824500	2.08890200	0.20371300		
	H	0.30915300	1.07173500	1.23816300		
	H	-2.96944800	0.98866200	-0.41891900		
	H	-3.06722600	-0.07515800	-1.81818900		
	H	-3.84877600	-0.54064900	-0.29316700		
	H	-1.68471400	-1.80978600	-0.60754700		
	H	-1.56946400	-0.75447100	0.79691600		

	H	2.25692500	-0.73559900	-0.22898400		
	H	0.99522300	-1.45379000	0.76692800		
	H	2.19813500	-3.23988900	-0.57153600		
	H	1.76888400	-2.39357700	-2.07241900		
	H	0.50975900	-3.12930600	-1.08624800		
$[Si(CF_3)_3]^-$	Si	-0.00150900	0.02234100	-1.09820700	Sum of electronic and zero-point Energies=	-1302.817928
	C	1.30956100	-1.21509400	-0.24420300	Sum of electronic and thermal Energies=	-1302.804401
	C	-1.62792300	-0.79964500	-0.28669600	Sum of electronic and thermal Enthalpies=	-1302.803457
	C	0.18638600	1.48742700	0.24258000	Sum of electronic and thermal Free Energies=	-1302.860126
	F	2.57859700	-0.68220800	-0.29657000		
	F	1.39269800	-2.39237100	-0.95480600		
	F	1.15020700	-1.61028600	1.05510400		
	F	-2.75840200	-0.11791200	-0.68059600		
	F	-1.80671800	-2.08067000	-0.76025100		
	F	-1.75005700	-0.90911200	1.07080000		
	F	1.29390900	2.25703900	-0.03825700		
	F	-0.87779100	2.35767400	0.15638900		
	F	0.29697100	1.20216900	1.57533300		
$[Si(CH_3)_3]^-$	Si	0.00000000	0.00000000	0.67294800	Sum of electronic and zero-point Energies=	-409.210769
	C	0.00000000	1.70695800	-0.28456900	Sum of electronic and thermal Energies=	-409.203837
	H	0.88270600	2.30942600	-0.02513700	Sum of electronic and thermal Enthalpies=	-409.202893
	H	0.00000000	1.57076800	-1.38273800	Sum of electronic and thermal Free Energies=	-409.239004
	H	-0.88270600	2.30942600	-0.02513700		
	C	-1.47826900	-0.85347900	-0.28456900		
	H	-1.36032500	-0.78538400	-1.38273800		
	H	-1.55866900	-1.91915900	-0.02513700		
	H	-2.44137400	-0.39026800	-0.02513700		
	C	1.47826900	-0.85347900	-0.28456900		
	H	2.44137400	-0.39026800	-0.02513700		
	H	1.55866900	-1.91915900	-0.02513700		
	H	1.36032500	-0.78538400	-1.38273800		
$Si(C_2F_5)_3H$	Si	0.00044600	-0.00037200	-0.53851000	Sum of electronic and zero-point Energies=	-2016.897521
	C	1.82754800	0.16773900	0.11887200	Sum of electronic and thermal Energies=	-2016.875472
	C	-0.76837500	-1.66669700	0.11815800	Sum of electronic and thermal Enthalpies=	-2016.874527
	C	-1.05937900	1.49819200	0.11719400	Sum of electronic and thermal Free Energies=	-2016.950621
	F	2.36094200	1.24250800	-0.55051800		
	F	1.84004000	0.45945700	1.45080300		
	F	-0.10569400	-2.66635100	-0.55204800		
	F	-0.52112400	-1.82409500	1.44988500		
	F	-2.25547400	1.42364500	-0.55472900		
	F	-1.32107900	1.36229300	1.44852400		
	H	0.00115800	-0.00066400	-2.00388300		
	C	-0.52228800	2.93600200	-0.10637600		
	C	2.80467700	-1.01543100	-0.10717000		
	C	-2.28204100	-1.91991600	-0.10656500		
	F	-0.16463400	3.08152900	-1.39532100		
	F	-1.45508300	3.84388100	0.18317700		
	F	0.54818900	3.15783600	0.66288100		
	F	2.75106500	-1.39588200	-1.39673900		
	F	2.46266000	-2.05482500	0.66062200		
	F	4.05734900	-0.66140100	0.18199100		
	F	-3.00970100	-1.10205600	0.66060200		
	F	-2.58540000	-1.68480600	-1.39621000		
	F	-2.60297300	-3.18100600	0.18467200		
$Si(C_2H_5)_3H$	Si	0.00032800	0.00002700	0.23600100	Sum of electronic and zero-point Energies=	-527.703231
	C	-1.27993000	-1.25835100	-0.35584300	Sum of electronic and thermal Energies=	-527.692412
	C	1.73020000	-0.47890300	-0.35635000	Sum of electronic and thermal Enthalpies=	-527.691468
	C	-0.44992700	1.73792600	-0.35560800	Sum of electronic and thermal Free Energies=	-527.738785
	H	0.00061700	-0.00025300	1.72871300		
	C	-1.78601500	2.29029200	0.17345100		
	C	-1.09181800	-2.69135300	0.17448600		

	C	2.87702300	0.40038100	0.17447800	
	H	-2.63399300	1.67294100	-0.14257200	
	H	-1.97301300	3.30760300	-0.18877900	
	H	-1.79895200	2.32524200	1.26857100	
	H	-0.45723400	1.73696000	-1.45527300	
	H	0.36461000	2.41753500	-0.07002500	
	H	1.73310600	-0.47008600	-1.45599100	
	H	1.91079000	-1.52474800	-0.07263300	
	H	2.91375500	0.39185900	1.26958300	
	H	3.85138500	0.05399600	-0.18847000	
	H	2.76678200	1.44411000	-0.13940100	
	H	-2.27565600	-0.89166800	-0.07128300	
	H	-1.27462400	-1.26501600	-1.45549500	
	H	-1.87957700	-3.36162400	-0.18783000	
	H	-1.11651200	-2.71911800	1.26961000	
	H	-0.13327300	-3.11806500	-0.14042800	
$Si(CF_3)_3H$	Si	-0.03872300	-0.06806700	-0.75973700	Sum of electronic and zero-point Energies= -1303.332228
	C	1.33373700	-1.29836900	-0.16703700	Sum of electronic and thermal Energies= -1303.318492
	C	-1.74423800	-0.98422400	-0.78396700	Sum of electronic and thermal Enthalpies= -1303.317548
	C	-0.13509900	1.41847700	0.47700800	Sum of electronic and thermal Free Energies= -1303.375449
	F	2.54236200	-0.68913200	-0.13696800	
	F	1.43399400	-2.34851800	-1.01558700	
	F	1.09851100	-1.79395800	1.06563100	
	F	-2.72855400	-0.15239800	-1.19876700	
	F	-1.71366900	-2.03039900	-1.64269900	
	F	-2.10083700	-1.46294700	0.42604000	
	F	1.04102000	2.08801600	0.51335500	
	F	-1.08777700	2.30017500	0.09248900	
	F	-0.42264600	1.03498400	1.73823200	
	H	0.28100900	0.42371500	-2.10465400	
$Si(CH_3)_3H$	Si	0.00000000	0.00002500	0.32200300	Sum of electronic and zero-point Energies= -409.825189
	C	0.00000000	1.78299700	-0.27659400	Sum of electronic and thermal Energies= -409.818241
	H	0.88394900	2.32444500	0.07929400	Sum of electronic and thermal Enthalpies= -409.817297
	H	0.00000000	1.83374600	-1.37209100	Sum of electronic and thermal Free Energies= -409.854553
	H	-0.88394900	2.32444500	0.07929400	
	C	-1.54411100	-0.89149600	-0.27652700	
	H	-1.58815200	-0.91675900	-1.37202100	
	H	-1.57095200	-1.92778400	0.07925000	
	H	-2.45501600	-0.39682700	0.07948500	
	C	1.54411100	-0.89149600	-0.27652700	
	H	2.45501600	-0.39682700	0.07948500	
	H	1.57095200	-1.92778400	0.07925000	
	H	1.58815200	-0.91675900	-1.37202100	
	H	0.00000000	0.00007100	1.81087500	

4.2 Oxidative addition - reaction pathway

Table S4. Cartesian coordinates and calculated thermochemical values of $[SiR_3]^-$ and SiR_3H for R = C_2F_5, C_2H_5, CF_3, and CH_3.

Molecule	Coordinates				Thermochemical values / Hartree
$C_6F_5(CF_3)$ (**a**-F)	C	-1.48602400	1.17486200	0.00001400	Sum of electronic and zero-point Energies= -1065.446584
	C	-0.09399200	1.22632500	-0.00001600	Sum of electronic and thermal Energies= -1065.434259
	C	0.67329900	0.05831500	-0.00012800	Sum of electronic and thermal Enthalpies= -1065.433315
	C	-0.01031000	-1.16311700	-0.00011400	Sum of electronic and thermal Free Energies= -1065.486181
	C	-1.39615700	-1.22994500	-0.00006300	
	C	-2.13760400	-0.05187100	-0.00002600	
	C	2.18665800	0.03841400	-0.00002200	
	F	-3.46610100	-0.10175800	-0.00001300	
	F	-2.19534600	2.30453200	0.00001400	
	F	0.46444400	2.43774900	0.00000900	

	F	-2.01716600	-2.40977200	0.00006800	
	F	0.67316200	-2.31117600	-0.00007700	
	F	2.66192000	-0.60875000	1.08669300	
	F	2.72621800	1.26395800	-0.00081700	
	F	2.66228900	-0.61010500	-1.08564000	
C_6F_6 (**b**-F)	C	0.00000000	1.39184600	0.00000000	Sum of electronic and zero-point Energies= -827.631541
	C	1.20535600	0.69594100	0.00000000	Sum of electronic and thermal Energies= -827.621932
	C	1.20535800	-0.69594300	0.00000000	Sum of electronic and thermal Enthalpies= -827.620988
	C	0.00000000	-1.39184600	0.00000000	Sum of electronic and thermal Free Energies= -827.665988
	C	-1.20535600	-0.69594100	0.00000000	
	C	-1.20535800	0.69594300	0.00000000	
	F	-2.36109500	1.36320600	0.00000000	
	F	0.00000000	2.72637600	0.00000000	
	F	2.36109200	1.36320800	0.00000000	
	F	-2.36109200	-1.36320800	0.00000000	
	F	0.00000000	-2.72637600	0.00000000	
	F	2.36109500	-1.36320600	0.00000000	
$C_{10}F_8$ (**c**-F)	C	2.43120400	-0.70391400	0.00004100	Sum of electronic and zero-point Energies= -1179.730527
	C	1.24632800	-1.39550500	0.00021600	Sum of electronic and thermal Energies= -1179.716456
	C	0.00000000	-0.72027200	0.00013700	Sum of electronic and thermal Enthalpies= -1179.715512
	C	0.00000100	0.72027000	-0.00013500	Sum of electronic and thermal Free Energies= -1179.771445
	C	1.24632700	1.39550400	-0.00031000	
	C	2.43120400	0.70391500	-0.00022600	
	C	-1.24633000	-1.39550500	0.00031200	
	C	-1.24632800	1.39550300	-0.00021500	
	C	-2.43120300	0.70391600	-0.00004100	
	C	-2.43120500	-0.70391600	0.00022700	
	F	-1.29879800	-2.73309800	0.00056600	
	F	-3.59808700	-1.35000500	0.00039200	
	F	-3.59808500	1.35000800	-0.00011900	
	F	-1.29879300	2.73309700	-0.00046200	
	F	1.29879400	2.73309600	-0.00056600	
	F	3.59808400	1.35000900	-0.00039400	
	F	3.59808500	-1.35000800	0.00011700	
	F	1.29880000	-2.73309700	0.00046100	
C_5F_5N (**d**-F)	C	0.00000000	1.12876300	-0.96592200	Sum of electronic and zero-point Energies= -744.460937
	C	0.00000000	-1.12876300	-0.96592200	Sum of electronic and thermal Energies= -744.452587
	C	0.00000000	1.20204300	0.42164200	Sum of electronic and thermal Enthalpies= -744.451643
	C	0.00000000	0.00000000	1.12490400	Sum of electronic and thermal Free Energies= -744.494017
	C	0.00000000	-1.20204300	0.42164200	
	F	0.00000000	-2.26094700	-1.67025100	
	F	0.00000000	-2.36733000	1.06927900	
	F	0.00000000	0.00000000	2.45053000	
	F	0.00000000	2.36733000	1.06927900	
	F	0.00000000	2.26094700	-1.67025100	
	N	0.00000000	0.00000000	-1.63647600	
C_5F_8 (**e**-F)	C	-1.10412700	0.00011500	0.00000000	Sum of electronic and zero-point Energies= -989.229440
	C	-0.15188100	-0.00007100	1.25723500	Sum of electronic and thermal Energies= -989.218711
	C	1.22288500	0.00001000	0.66649800	Sum of electronic and thermal Enthalpies= -989.217767
	C	1.22288500	0.00001000	-0.66649800	Sum of electronic and thermal Free Energies= -989.266837
	C	-0.15188100	-0.00007100	-1.25723500	
	F	2.28067800	0.00005300	1.45453300	
	F	2.28067800	0.00005300	-1.45453300	
	F	-0.36899800	1.09009200	-2.03456900	
	F	-0.36902500	-1.09039100	-2.03428500	
	F	-1.88883100	-1.09139100	0.00000000	
	F	-1.88839900	1.09188700	0.00000000	
	F	-0.36902500	-1.09039100	2.03428500	
	F	-0.36899800	1.09009200	2.03456900	
C_3F_6 (**f**-F)	C	-0.24572700	-0.47197500	0.00000700	Sum of electronic and zero-point Energies= -713.342300
	C	-1.41710700	0.16193300	0.00000300	Sum of electronic and thermal Energies= -713.334345

	C	1.12537100	0.13463700	0.00005200	Sum of electronic and thermal Enthalpies=	-713.333401
	F	-1.57668100	1.46225300	0.00001500	Sum of electronic and thermal Free Energies=	-713.376359
	F	-2.56320600	-0.47763600	-0.00007200		
	F	-0.23217500	-1.81651100	-0.00009200		
	F	1.82505800	-0.26226300	1.08311700		
	F	1.08017300	1.47340300	0.00000100		
	F	1.82513900	-0.26230800	-1.08301100		
$[Si(CF_3)_3]^-$	Si	-0.04042200	0.01117600	-1.07066900	Sum of electronic and zero-point Energies=	-1302.564128
	C	1.10960500	-0.88527100	0.28399300	Sum of electronic and thermal Energies=	-1302.550761
	C	-1.76482600	-0.72597200	-0.40364300	Sum of electronic and thermal Enthalpies=	-1302.549817
	C	-0.17341100	1.74796500	-0.10787400	Sum of electronic and thermal Free Energies=	-1302.605381
	F	2.32207800	-0.24323900	0.38648500		
	F	1.41841900	-2.16545600	-0.11368900		
	F	0.67582600	-1.02324000	1.57458800		
	F	-2.80855000	-0.29154800	-1.18771800		
	F	-1.78124100	-2.09672400	-0.51853800		
	F	-2.17379700	-0.47665000	0.87847700		
	F	0.91276700	2.54224000	-0.39375200		
	F	-1.26254700	2.46318800	-0.54950200		
	F	-0.26635000	1.77467000	1.25733800		
$[Si(CF_3)_3\{C_6F_4(CF_3)\}F]^-$	C	1.01587400	1.18372600	-0.24400000	Sum of electronic and zero-point Energies=	-2368.112329
[**a**-$Si(CF_3)_3F]^-$	C	2.40506900	1.23222100	-0.18389400	Sum of electronic and thermal Energies=	-2368.085358
F and CF_3 axial	C	3.14951100	0.05426800	-0.09268400	Sum of electronic and thermal Enthalpies=	-2368.084414
	C	2.43148100	-1.14461500	-0.06619000	Sum of electronic and thermal Free Energies=	-2368.171483
	C	1.04505800	-1.15908200	-0.12353700		
	C	0.28701500	0.00303600	-0.21247700		
	C	4.65648100	0.00266900	-0.02270800		
	F	0.36603600	2.36266000	-0.33600100		
	F	2.99299500	2.43590300	-0.21762100		
	F	0.44068000	-2.36210400	-0.09847000		
	F	3.08894000	-2.31243100	0.01530300		
	F	5.07539700	-0.60081000	1.11412600		
	F	5.22999700	1.21475100	-0.05705900		
	F	5.17187700	-0.70788100	-1.05314200		
	Si	-1.64659800	0.02762300	-0.34959100		
	C	-2.75989400	-1.57675100	-0.71377600		
	C	-2.69018300	1.71500600	-0.26332600		
	C	-1.75999000	-0.20287400	1.67095100		
	F	-1.50346600	0.19286400	-2.02502400		
	F	-2.07908400	-2.51603600	-1.43278800		
	F	-3.83979400	-1.23974500	-1.48080700		
	F	-3.27317700	-2.23769900	0.35713800		
	F	-4.03118400	1.42864000	-0.24712700		
	F	-2.50638000	2.54113900	-1.32441500		
	F	-2.46952400	2.48520800	0.83740000		
	F	-0.99267900	0.71287800	2.35187400		
	F	-3.00996900	-0.09181300	2.21429000		
	F	-1.28623900	-1.42023100	2.09389200		
$[Si(CF_3)_3\{C_6F_4(CF_3)\}F]^-$	C	0.73233400	0.25189800	-1.06708900	Sum of electronic and zero-point Energies=	-2368.114680
[**a**-$Si(CF_3)_3F]^-$	C	2.11217900	0.29291000	-1.24572400	Sum of electronic and thermal Energies=	-2368.087522
CF_3 and CF_3 axial	C	2.97188700	0.04171600	-0.17438800	Sum of electronic and thermal Enthalpies=	-2368.086578
	C	2.37931400	-0.24746000	1.05864000	Sum of electronic and thermal Free Energies=	-2368.174543
	C	1.00036100	-0.28017200	1.20433700		
	C	0.12879800	-0.03183800	0.14902700		
	C	4.47896400	0.06283400	-0.27032800		
	F	-0.03132500	0.50652700	-2.14856100		
	F	2.57751600	0.57945000	-2.46897000		
	F	0.51040400	-0.56841000	2.42541000		
	F	3.15226800	-0.49937300	2.12661900		
	F	5.01116000	0.97977000	0.57077700		
	F	4.92723500	0.35251800	-1.50066400		

	F	5.00870000	-1.13439800	0.07193200		
	Si	-1.79412000	-0.09111600	0.39732100		
	C	-2.97195400	0.26310300	-1.15960800		
	C	-1.79297000	1.84508700	0.97036300		
	F	-4.27254200	0.16526000	-0.73588500		
	F	-2.85493900	-0.61114900	-2.19324400		
	F	-2.85569500	1.49950000	-1.71115900		
	F	-1.12809200	2.69653900	0.12372500		
	F	-3.03599000	2.39557200	1.13160900		
	F	-1.16844700	2.00935100	2.18311100		
	C	-1.78791000	-2.08361400	0.07098600		
	F	-3.03056200	-2.64924100	-0.02845600		
	F	-1.16775400	-2.75791900	1.09545500		
	F	-1.11873000	-2.48329200	-1.05900000		
	F	-2.56635700	-0.41527700	1.80843700		
$[Si(CF_3)_3(C_6F_5)F]^-$	C	1.64908600	1.16935500	-0.24716900	Sum of electronic and zero-point Energies=	-2130.296984
[**b**-$Si(CF_3)_3F]^-$	C	3.03693400	1.20437400	-0.18491200	Sum of electronic and thermal Energies=	-2130.272779
	C	3.73978000	0.00916600	-0.09055200	Sum of electronic and thermal Enthalpies=	-2130.271835
	C	3.04460800	-1.19301700	-0.05955200	Sum of electronic and thermal Free Energies=	-2130.351896
	C	1.65445900	-1.17936800	-0.11993100		
	C	0.91034100	-0.00846200	-0.21321100		
	F	1.00991200	2.35458700	-0.34261800		
	F	3.70196700	2.36876200	-0.21530500		
	F	1.03476000	-2.37610200	-0.09425200		
	F	3.71892200	-2.34945900	0.02597400		
	F	5.07713000	0.01660100	-0.03117500		
	Si	-1.01749600	0.03426400	-0.35028800		
	F	-0.89262900	0.21300800	-2.02757600		
	C	-2.13840200	-1.56438800	-0.72286700		
	C	-2.05077500	1.72961600	-0.25301900		
	C	-1.14298400	-0.20507900	1.67098200		
	F	-1.86206300	2.56231200	-1.30891100		
	F	-3.39410700	1.45219000	-0.24015300		
	F	-1.82776800	2.49330100	0.85214500		
	F	-2.39595600	-0.09581200	2.21098600		
	F	-0.37903400	0.70777600	2.36023300		
	F	-0.67193200	-1.42384700	2.09397600		
	F	-1.47274800	-2.49077300	-1.47283400		
	F	-2.63232000	-2.24693500	0.34414100		
	F	-3.23339300	-1.21370700	-1.46292000		
$[Si(CF_3)_3(C_{10}F_7)F]^-$	C	0.13054300	0.30026600	-0.22401300	Sum of electronic and zero-point Energies=	-2482.394098
[**c**-$Si(CF_3)_3F]^-$	C	-0.81017400	-0.69622900	-0.15710300	Sum of electronic and thermal Energies=	-2482.365324
	C	-2.21349000	-0.48207500	-0.10298000	Sum of electronic and thermal Enthalpies=	-2482.364379
	C	-2.68318300	0.87799800	-0.12298400	Sum of electronic and thermal Free Energies=	-2482.455399
	C	-1.71625100	1.90688300	-0.19876600		
	C	-0.37838600	1.61071800	-0.24787700		
	C	-3.17477200	-1.52306500	-0.02750500		
	C	-4.07879100	1.11594600	-0.06690600		
	C	-4.97423100	0.08009900	0.00631100		
	C	-4.51843300	-1.25127400	0.02647200		
	F	-2.80275700	-2.80996200	-0.00484000		
	F	-5.41617000	-2.24080000	0.09972200		
	F	-6.28941500	0.31500700	0.05931200		
	F	-4.56467000	2.36675900	-0.08422800		
	F	-2.09646500	3.19720700	-0.22244600		
	F	0.49013800	2.63867600	-0.32001600		
	F	-0.37978700	-1.97677600	-0.14895100		
	Si	2.03143500	-0.02244300	-0.34870900		
	C	2.09179500	-0.30848200	1.67078400		
	C	2.81960300	-1.80105800	-0.74864900		
	C	3.37926200	1.43440300	-0.20891600		

	F	1.95765600	0.21656300	-2.02183800		
	F	3.40856300	2.27406300	-1.27556600		
	F	4.63905900	0.89870200	-0.12913700		
	F	3.25969500	2.24234800	0.88120700		
	F	2.04027600	-2.53071400	-1.59792300		
	F	4.01734000	-1.64851600	-1.39243300		
	F	3.07983300	-2.62245200	0.30278600		
	F	1.38501200	-1.40826900	2.08716300		
	F	1.52309700	0.73653900	2.36289200		
	F	3.33959100	-0.45621600	2.21482100		
$[Si(CF_3)_3(C_5F_4N)F]^-$	C	-3.25950700	-1.11413600	-0.07396400	Sum of electronic and zero-point Energies=	-2047.126042
[**d**-$Si(CF_3)_3F]^-$	C	-3.25084000	1.12405900	-0.18584400	Sum of electronic and thermal Energies=	-2047.103057
	C	-1.87121600	-1.17386900	-0.12393400	Sum of electronic and thermal Enthalpies=	-2047.102113
	C	-1.11586800	-0.00964400	-0.21226100	Sum of electronic and thermal Free Energies=	-2047.179677
	C	-1.86418700	1.16240900	-0.24502300		
	F	-3.94754300	2.26943800	-0.21526800		
	F	-1.24992600	2.35882000	-0.33533900		
	F	-1.27783700	-2.38140300	-0.09295600		
	F	-3.96562200	-2.25140900	0.00674300		
	N	-3.93901100	0.00851000	-0.10233800		
	Si	0.81847400	0.02972000	-0.34771200		
	F	0.66557600	0.18302300	-2.02279800		
	C	1.84107200	1.72880700	-0.27080400		
	C	1.94554200	-1.56572500	-0.70451800		
	C	0.92995500	-0.19102000	1.67237000		
	F	0.14888200	0.71678200	2.34780600		
	F	0.47185100	-1.41314700	2.09800600		
	F	2.17742000	-0.06090500	2.21624100		
	F	2.47293600	-2.21057100	0.36903100		
	F	3.01585300	-1.22536200	-1.48281600		
	F	1.26760400	-2.51814900	-1.40875600		
	F	1.61186300	2.49981300	0.82743400		
	F	3.18503500	1.45853700	-0.25597700		
	F	1.64443200	2.54776100	-1.33488600		
$[Si(CF_3)_3(C_5F_7)F]^-$	C	3.14189000	-0.60375200	-0.19006600	Sum of electronic and zero-point Energies=	-2291.896685
[**e**-$Si(CF_3)_3F]^-$	C	2.98798500	0.93081800	0.06287400	Sum of electronic and thermal Energies=	-2291.871316
	C	1.50646700	1.12641800	-0.02359800	Sum of electronic and thermal Enthalpies=	-2291.870372
	C	0.75110600	0.03313000	-0.12830700	Sum of electronic and thermal Free Energies=	-2291.954057
	C	1.67004400	-1.16060300	-0.14734000		
	F	1.06607700	2.38362000	0.01607500		
	F	1.51309300	-1.93704200	0.96584800		
	F	1.45668500	-1.97553400	-1.21460200		
	F	3.69148900	-0.81893100	-1.40519300		
	F	3.93343000	-1.18617200	0.73300000		
	F	3.67029600	1.66460700	-0.85502900		
	F	3.48380000	1.27793200	1.28421100		
	Si	-1.16476300	-0.02645800	-0.33878100		
	C	-1.40775400	0.28434500	1.65482900		
	C	-2.18736300	-1.72832500	-0.26106700		
	C	-2.14141900	1.62655100	-0.81756000		
	F	-0.94353700	-0.32604200	-1.98559900		
	F	-1.58193200	2.28437900	-1.86904200		
	F	-3.41474300	1.30493800	-1.21012500		
	F	-2.28296200	2.56094500	0.15814400		
	F	-0.68480200	1.34906100	2.13285800		
	F	-2.68957500	0.51285700	2.07099400		
	F	-0.97010200	-0.78803900	2.39386300		
	F	-3.00444300	-1.90500600	0.81049100		
	F	-1.32728100	-2.79451900	-0.24911800		
	F	-2.98427700	-1.90495300	-1.34962600		
$[Si(CF_3)_3(C_3F_5)F]^-$	C	-2.25759700	-1.02405200	-0.14433500	Sum of electronic and zero-point Energies=	-2015.995336

[**f**-Si(CF_3)$_3$F]$^-$	C	-0.93724900	-1.20814000	-0.17447800	Sum of electronic and thermal Energies= -2015.972645
	C	-2.98625600	0.27769800	-0.20915800	Sum of electronic and thermal Enthalpies= -2015.971701
	F	-0.54906500	-2.51310700	-0.09805300	Sum of electronic and thermal Free Energies= -2016.050076
	F	-3.11631200	-2.06587300	-0.04494100	
	F	-3.79899600	0.34535300	-1.28845800	
	F	-2.13372400	1.31475600	-0.27410100	
	F	-3.77633700	0.46121600	0.87309900	
	Si	0.59206600	-0.01938300	-0.34082800	
	C	0.87872900	-0.21849400	1.66765000	
	C	2.29050000	-0.91988200	-0.80926400	
	F	-0.21618600	0.18998000	2.39209000	
	F	2.06256600	-2.12744600	-1.40744700	
	F	3.15502000	-1.17160900	0.20848100	
	F	1.07647600	-1.52108300	2.05279900	
	F	3.00169600	-0.18835600	-1.71533200	
	F	1.93222900	0.47530300	2.19465000	
	C	0.78893500	1.95520500	-0.19228100	
	F	0.26452300	2.65979900	-1.22542800	
	F	0.26576400	2.51703600	0.93167600	
	F	2.12495000	2.26763600	-0.17449400	
	F	0.26836300	0.14498900	-1.98645300	

Table S5. Cartesian coordinates and calculated thermochemical values of the transition states.

Molecule	Coordinates				Thermochemical values / Hartree
[Si(CF_3)$_3$]$^-$ + $C_6F_5(CF_3)$	Si	1.71492900	-0.00696400	0.12219300	Sum of electronic and zero-point Energies= -2367.994378
(**a**-F)	C	1.25720500	-0.72190600	-1.65150900	Sum of electronic and thermal Energies= -2367.967110
	C	3.33990200	-1.04976900	0.51706800	Sum of electronic and thermal Enthalpies= -2367.966166
	C	2.52489100	1.70675600	-0.40993200	Sum of electronic and thermal Free Energies= -2368.055923
	F	0.07269100	-0.19578600	-2.08809300	
	F	1.06739000	-2.07419800	-1.59696700	
	F	2.14474900	-0.51132500	-2.66173000	
	F	4.09182500	-0.43080800	1.47517000	
	F	3.02274600	-2.27699900	1.02237300	
	F	4.18883100	-1.29164400	-0.52207500	
	F	1.63642000	2.46009800	-1.12443300	
	F	2.86257500	2.45479000	0.67989500	
	F	3.65353200	1.64297800	-1.17196000	
	C	-1.08473900	1.24154100	1.16991500	
	C	-2.28939600	1.24225000	0.49863200	
	C	-2.95513700	0.05233000	0.16137900	
	C	-2.30051700	-1.13361000	0.53946400	
	C	-1.09954600	-1.14130100	1.20721600	
	C	-0.37965000	0.05139900	1.47766000	
	F	0.35252500	0.06687700	2.65870900	
	F	-0.47797700	2.41790900	1.43952700	
	F	-2.81256200	2.44121700	0.19040400	
	F	-0.50501300	-2.31617300	1.50359600	
	F	-2.87052200	-2.31885400	0.25171800	
	C	-4.28522700	-0.02454800	-0.51493200	
	F	-5.21833600	-0.65034500	0.25719900	
	F	-4.23378500	-0.73621000	-1.67280200	
	F	-4.79461300	1.18054600	-0.82724800	
[Si(CF_3)$_3$]$^-$ + C_6F_6 (**b**-F)	Si	1.12064100	-0.00837900	0.16157900	Sum of electronic and zero-point Energies= -2130.170797
	C	0.78769400	0.48329300	-1.71073000	Sum of electronic and thermal Energies= -2130.146278
	C	2.11627000	-1.68858200	-0.09373200	Sum of electronic and thermal Enthalpies= -2130.145333
	C	2.60875700	1.20931400	0.59560600	Sum of electronic and thermal Free Energies= -2130.226948
	F	0.49191400	1.81319300	-1.81744900	
	F	-0.30205800	-0.18272000	-2.19765200	
	F	1.78818700	0.25055900	-2.60322700	
	F	2.35077100	-2.31028700	1.09684800	

F	1.40302800	-2.57285000	-0.85018700
F	3.33529500	-1.58426900	-0.69656700
F	2.15455800	2.44180300	0.95777100
F	3.32076900	0.74011800	1.66220500
F	3.52205500	1.43327700	-0.39259200
C	-1.71489100	1.18528600	0.85264000
C	-2.88660600	1.19356200	0.11865500
C	-3.50845200	0.00251700	-0.24516600
C	-2.90591900	-1.19323900	0.13571300
C	-1.73387200	-1.19428400	0.86918300
C	-1.02210300	-0.00787300	1.19394300
F	-0.42831700	-0.00531400	2.48904600
F	-1.10353700	2.36477800	1.12027900
F	-3.44518300	2.36674700	-0.23218500
F	-1.14629100	-2.38095100	1.15803000
F	-3.48463300	-2.36194500	-0.19753200
F	-4.69369800	0.00756700	-0.89221200

References

[1] N. Tiessen, M. Keßler, B. Neumann, H.-G. Stammler, B. Hoge, N. Tiessen, M. Keßler, B. Neumann, H.-G. Stammler, B. Hoge, *Angew. Chem. Int. Ed.* **2021**, *60*, 12231–12238; Angew. Chem. 2021, 133,12231 –12238.

[2] O. V. Dolomanov, L. J. Bourhis, R. J. Gildea, J. A. K. Howard, H. Puschmann, O. V. Dolomanov, L. J. Bourhis, R. J. Gildea, J. A. K. Howard, H. Puschmann, *J. Appl. Cryst.* **2009**, *42*, 339.

[3] G. M. Sheldrick, G. M. Sheldrick, *Acta Cryst.* **2015**, *A71*, 3.

[4] G. M. Sheldrick, G. M. Sheldrick, *Acta Cryst.* **2015**, *C71*, 3.

[5] M. J. Frisch, G. W. Trucks, H. B. Schlegel, G. E. Scuseria, M. A. Robb, J. R. Cheeseman, G. Scalmani, V. Barone, B. Mennucci, G. A. Petersson, H. Nakatsuji, M. Caricato, X. Li, H. P. Hratchian, A. F. Izmaylov, J. Bloino, G. Zheng, J. L. Sonnenberg, M. Hada, M. Ehara, K. Toyota, R. Fukuda, J. Hasegawa, M. Ishida, T. Nakajima, Y. Honda, O. Kitao, H. Nakai, T. Vreven, J. A. Montgomery, Jr., J. E. Peralta, F. Ogliaro, M. Bearpark, J. J. Heyd, E. Brothers, K. N. Kudin, V. N. Staroverov, T. Keith, R. Kobayashi, J. Normand, K. Raghavachari, A. Rendell, J. C. Burant, S. S. Iyengar, J. Tomasi, M. Cossi, N. Rega, J. M. Millam, M. Klene, J. E. Knox, J. B. Cross, V. Bakken, C. Adamo, J. Jaramillo, R. Gomperts, R. E. Stratmann, O. Yazyev, A. J. Austin, R. Cammi, C. Pomelli, J. W. Ochterski, R. L. Martin, K. Morokuma, V. G. Zakrzewski, G. A. Voth, P. Salvador, J. J. Dannenberg, S. Dapprich, A. D. Daniels, O. Farkas, J. B. Foresman, J. V. Ortiz, J. Cioslowski, D. J. Fox, *Gaussian 09, Revision D.01*, Gaussian, Inc., Willingford CT, **2013**.
